# Mapping genetic modifiers of epimutation rates identifies *VIM2*/*4* as dosage-sensitive negative regulators of CG methylation maintenance

**DOI:** 10.1038/s41477-026-02396-4

**Published:** 2026-09-03

**Authors:** Zhilin Zhang, Wilma Wanney, Yangyang Xu, Johan Zicola, Angela M. Hancock, Robert J. Schmitz, Frank Johannes

**Affiliations:** 1 Plant Epigenomics, Technical University of Munich, Freising, Germany; 2 Department of Genetics, University of Georgia, Athens, GA USA; 3 Department of Plant Developmental Biology, Max Planck Institute for Plant Breeding Research, Cologne, Germany

**Keywords:** Epigenomics, Plant evolution, Plant genetics

## Abstract

Spontaneous epimutations are stochastic gains and losses of cytosine methylation that arise from imperfect maintenance across cell divisions. At CG sites, such epimutations can be inherited across generations in plants and constitute a major source of CG methylation (mCG) diversity. However, why the fidelity of mCG inheritance varies among genotypes, and how this variation relates to steady-state mCG levels, remains poorly understood. Here we tracked DNA methylation over 10 generations in ~400 mutation-accumulation lines derived from ~70 *Arabidopsis thaliana* Ler × Cvi recombinant inbred founders. By treating methylation gain and loss rates as quantitative molecular traits, we mapped a major-effect locus to a Cvi-derived deletion between VARIANT IN METHYLATION (*VIM*)*2* and *VIM4*, two key components of the METHYLTRANSFERASE 1-dependent mCG maintenance pathway. Lines carrying this deletion showed elevated *VIM2*/*4* (*VIM2* and *VIM4*) expression, a rapid shift of genome-wide mCG towards a lower steady state and reduced fidelity of methylation inheritance across generations. Complementary overexpression and loss-of-function experiments identify *VIM2*/*4* as dosage-sensitive negative regulators of mCG maintenance, in contrast to the canonical positive role of VIM-family proteins in mCG. Together, our results support a punctuated-equilibrium model of DNA methylome evolution, in which naturally segregating modifiers of mCG homeostasis can produce abrupt shifts in methylation state and alter the rate at which heritable epigenetic variation accumulates in plant genomes.

## Abstract (web summary)

This study shows that epimutation rates are heritable quantitative traits and identifies *VIM2* and *VIM4* as dosage-sensitive negative regulators of CG methylation levels and inheritance fidelity.

## Main

DNA cytosine methylation is a central epigenetic modification in plants, where it contributes to transposable element (TE) silencing, chromatin organization and gene regulation^1^. Natural populations harbour extensive methylome variation^2,3^, and in some cases, this variation is associated with stable heritable phenotypic differences^4–6^. In plants, methylation occurs in three sequence contexts (CG, CHG and CHH, where H = A, T, C), which are maintained by partly distinct pathways^1,7^. Among these, CG methylation (mCG) is particularly well defined at the mechanistic level. During DNA replication, mCG is copied by METHYLTRANSFERASE 1 (*MET1*, AT5G49160, the plant homologue of mammalian *DNMT1*) together with VARIANT IN METHYLATION (*VIM*, homologues of mammalian *UHRF1*) proteins, which recognize hemimethylated CG sites and promote restoration of full methylation on the newly synthesized strand^1,8–16^. Thus, the *MET1*/*VIM* system lies at the molecular core of mCG inheritance.

Despite the fidelity of this maintenance machinery, mCG states are not transmitted perfectly across cell divisions and generations. Multigenerational studies show that stochastic gains and losses of mCG can arise without underlying DNA sequence change^17–23^. These events, termed ‘spontaneous epimutations’, are thought to reflect imperfect copying by the mCG maintenance system^24^. They occur at rates far higher than DNA sequence mutation^19–22^ and accumulate in a largely clock-like manner over evolutionary timescales^17,25^. Genome-wide analyses further show that epimutation rates vary strongly across genomic features^17,18,22,26^ and that these biases help explain standing patterns of mCG diversity among natural accessions^22,23^. Together, these findings suggest that natural mCG variation should be viewed not simply as a static molecular trait but as the outcome of an ongoing gain–loss process^27,28^.

This dynamic view is important because the steady-state level of mCG and the stability of that level are not the same property. The relative balance of methylation gain and loss determines the equilibrium mCG level observed in a given plant genome, whereas the overall rate of turnover determines how rapidly epigenetic drift accumulates around that equilibrium^22,27^. As a result, two genotypes can show similar methylation levels while differing markedly in maintenance fidelity or conversely differ in methylation level without large differences in ongoing turnover. Previous methylation quantitative trait locus (meQTL) mapping studies in natural populations have relied on cross-sectional methylome data and therefore captured methylation states at one timepoint^2,3,29,30^. Such data are powerful for mapping genetic loci that affect steady-state methylation differences among genotypes, but they cannot reveal whether a genetic variant primarily shifts the equilibrium, alters the fidelity of transgenerational copying or couples both effects.

Because MET1 and VIM proteins form the core mCG maintenance machinery, natural variation in mCG dynamics could, in principle, arise from altered activity, dosage or stoichiometric balance within this complex. However, changes in CG inheritance can also arise indirectly through perturbations outside the canonical *MET1*/*VIM* pathway. Previous work showed, for example, that mutants in CHROMOMETHYLASE 3 (*CMT3*, AT1G69770) gene, which encodes a DNA methyltransferase primarily responsible for maintaining CHG methylation^31^, can display increased accumulation of spontaneous CG epimutations despite *CMT3* not being a canonical component of the *MET1*/*VIM* copying machinery^18,32^. Thus, a key challenge is to determine whether natural variation in mCG stability reflects direct perturbation of the core maintenance machinery or secondary effects from other epigenetic pathways.

Natural methylation outliers provide an attractive entry point to address this problem. Such genotypes may carry alleles that perturb mCG maintenance strongly enough to generate pronounced methylation phenotypes yet subtly enough to remain compatible with long-term viability in natural populations. We reasoned that the *Arabidopsis thaliana* accession Cvi-0 is particularly informative in this regard. Cvi-0 is a striking natural outlier with globally reduced mCG levels^3^, but the molecular basis of this phenotype, and its relationship to spontaneous epimutation dynamics, are unknown. By combining genetic mapping of steady-state methylation with direct estimates of transgenerational mCG gain and loss rates, this system provides an opportunity to genetically dissect whether natural variation in mCG arises primarily through direct effects on the core *MET1*/*VIM* maintenance complex or through indirect perturbations elsewhere in the epigenetic network. More generally, it allows us to determine whether the Cvi phenotype reflects a simple shift in methylation equilibrium or a broader change in maintenance fidelity.

To address this, we generated a large mutation accumulation mapping population derived from Ler × Cvi recombinant inbred lines (RILs) as founders to dissect the genetic and mechanistic basis of natural variation in mCG maintenance. Treating both steady-state mCG levels and spontaneous epimutation rates as quantitative molecular traits, we identify a major-effect locus on chromosome 1 that influences both properties. We show that the causal variant is a Cvi-specific structural deletion between *VIM2* (AT1G66050) and *VIM4* (AT1G66040) that elevates *VIM2*/*4* (*VIM2* and *VIM4*) expression. Unexpectedly, an increased VIM2/4 dosage does not enhance CG maintenance but instead lowers the mCG steady state and reduces the fidelity of methylation inheritance across generations. These findings identify the mechanistic basis of a natural mCG outlier, reveal previously unrecognized functions of *VIM2*/*4* and show how naturally segregating variants can jointly reshape both the level and long-term stability of DNA methylation in plants.

## Results

### Steady-state mCG levels and epimutation rates are genotype dependent

**Fig. 1:**
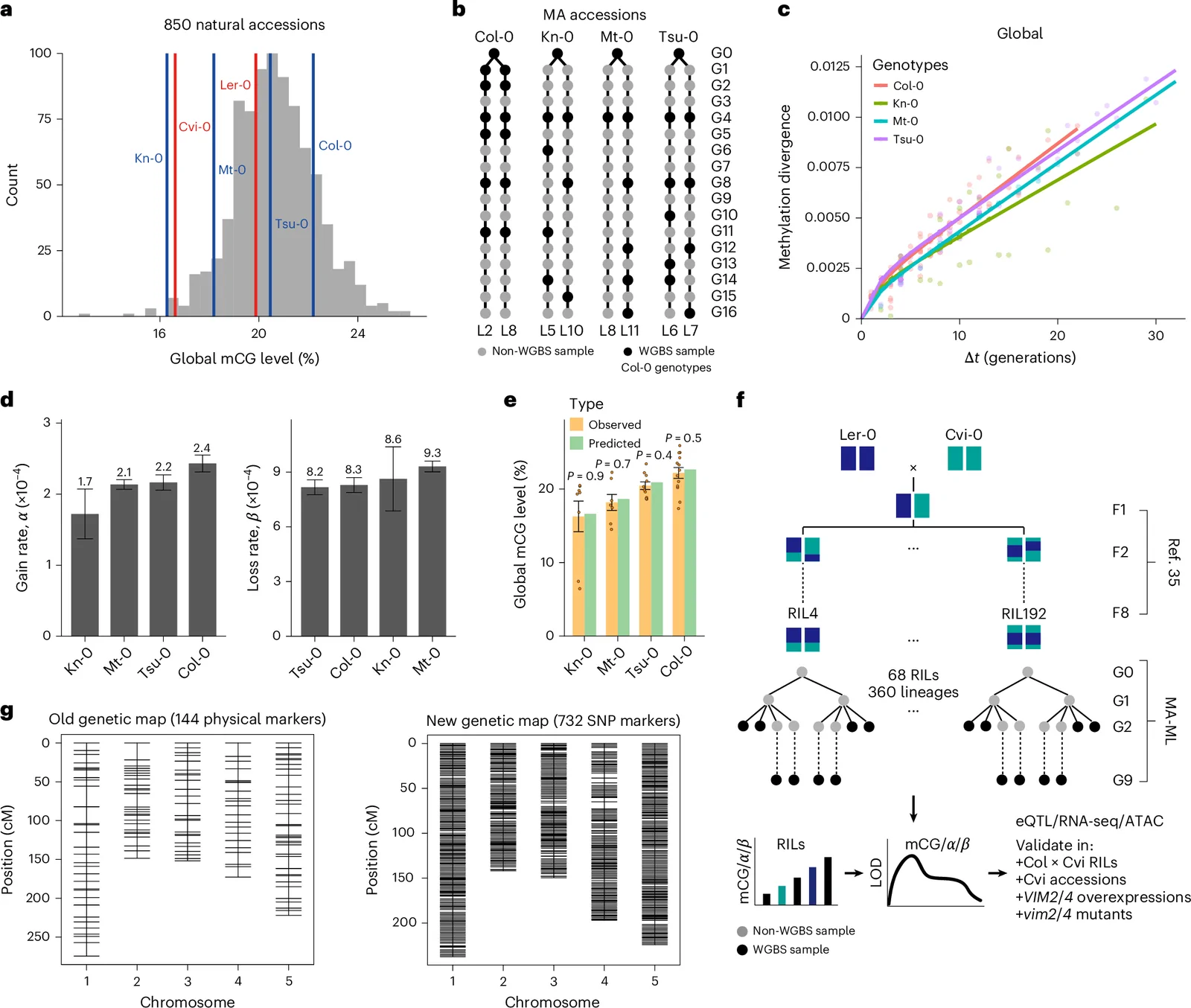
Genotype-dependent methylation variation and the establishment of an MA-ML. **a**, Distribution of genome-wide mCG levels across 850 natural *A. thaliana* accessions. Blue lines represent accessions selected for MA experiments (Col-0, Kn-0, Mt-0 and Tsu-0), and red lines indicate the Ler-0 and Cvi-0 lines used in the MA-ML population. **b**, Schematic of MA pedigrees established for 4 selected accessions spanning the natural mCG range, propagated for up to 16 generations by single-seed descent. Black circles indicate samples sequenced by WGBS. G, generation; L, lineage. **c**, Global methylation divergence as a function of generational time (Δ*t*, generations) in four MA lines, illustrating genotype-specific differences in mCG stability. Curves represent model fits for each genotype. **d**, Comparison of genome-wide spontaneous mCG gain rates (*α*, left) and loss rates (*β*, right) among the four genotypes. Bars represent point estimates of mathematical model parameters for epimutation rates, and error bars denote the standard error (s.e.) of these estimates. Estimates are based on the following numbers of biologically independent WGBS samples derived from individual plants: *n* = 8 (Kn-0), 7 (Mt-0), 10 (Tsu-0) and 13 (Col-0). The sample sizes used in epimutation rate estimations are 1/2 × *n* × (*n* − 1), where *n* is the number of sequenced samples in each MA pedigree. **e**, Observed versus predicted genome-wide mCG levels based on estimated epimutation rates, indicating that genotypes are at methylation equilibrium. Bars show mean values; error bars represent standard error of the mean (s.e.m.); *P* values from two-sided one-sample *t*-tests comparing observed and predicted values are indicated above bars. The predicted values are single theoretical equilibrium estimates without variance. Overlaid dots represent biologically independent WGBS samples derived from individual plants (*n* = 8, 7, 10 and 13 for Kn-0, Mt-0, Tsu-0 and Col-0, respectively). **f**, Overview of the experimental framework used to dissect the genetic basis of methylation dynamics. The MA-ML were constructed from Ler × Cvi RILs. Each RIL was used to initiate 8 independent MA lineages, propagated for 9 generations (single-seed descent), resulting in 68 MA-ML pedigrees. Additional MA lines were also derived from the Ler-0 and Cvi-0 parental accessions. WGBS datasets were generated from early (G2) and late (G9) generations to quantify mCG changes over time, enabling subsequent QTL analyses. These data were integrated with previously published eQTL resources derived from Ler × Cvi RILs, along with newly generated RNA-seq and chromatin accessibility profiles from ATAC-seq across generations, to support multilayered trait dissection. The resulting findings were further validated using independent Col × Cvi RILs, natural Cvi accessions, *VIM2*/*4* overexpression lines and *vim2*/*4* mutants. F, generation of RIL; G, generation of MA-ML. **g**, Comparison of the original genetic map (144 physical markers, left) used in previous studies versus the newly constructed genetic map (732 SNP markers, right) generated from SNPs called from WGBS data in this study, substantially enhancing mapping resolution. Source data

To quantify natural variation in mCG, we re-analysed published DNA methylomes from 850 *A. thaliana* accessions^3^. Genome-wide mCG levels were ~2.5-fold more variable among accessions than non-mCG (Fig. 1a and Supplementary Table 1), emphasizing the relevance of this cytosine context for natural variation. To assess genotype-specific differences in mCG stability across generations, we previously established mutation accumulation (MA) pedigrees for four selected accessions (Col-0, Kn-0, Mt-0 and Tsu-0) that span the natural range of mCG levels and propagated them for up to 16 selfings^17^ (Fig. 1a,b). Epimutation analysis revealed up to 1.4-fold differences in the combined mCG gain (*α*) and loss (*β*) rates, indicating that some genotypes are more error prone at maintaining mCG states transgenerationally (Fig. 1c,d). Predicted mCG levels based on these rates closely matched empirical observations in each accession (Fig. 1e), implying that all genotypes are at methylation equilibrium^22,27^. These findings demonstrate that steady-state mCG levels and epimutation rates are determined, in part, by genetic background.

### Construction of MA-mapping line population

To identify genetic factors contributing to variation in mCG maintenance dynamics, we constructed a population of MA mapping lines (MA-ML) using Landsberg erecta (Ler-0) × Cape Verde Islands (Cvi-0) RILs as founder genotypes^33^. Each RIL was used to initiate eight independent MA lineages, propagated by single-seed descent for nine generations (Fig. 1f). The Ler × Cvi RILs were chosen because their parental accessions have highly contrasting steady-state mCG levels, with Cvi-0 scoring in the first percentile of the natural distribution and Ler-0 ranking substantially higher (Fig. 1a). We expected that this wide cross maximizes our ability to detect genetic variations underlying mCG differences. Moreover, the Ler × Cvi RILs have been extensively characterized in prior quantitative trait locus (QTL) studies of both classical and molecular phenotypes, including transcript, metabolite and protein abundance^34,35^, thus providing a rich framework for integrative analysis. To capture methylation dynamics across generations, we performed whole-genome bisulfite sequencing (WGBS) on early- and late-generation individuals from 68 MA-ML pedigrees as well as from MA lines derived from the Ler-0 and Cvi-0 parental accessions, yielding 371 WGBS datasets in total (Fig. 1f and Supplementary Table 2). Using these data, we saturated the existing genetic map with additional single-nucleotide polymorphisms (SNPs) called from the WGBS reads (Methods). This resulted in a genetic map comprising 732 SNP markers, with an average spacing of 1.3 centimorgans (cM) and a maximum interval of 8.0 cM, substantially improving on the previously published map (Fig. 1g, Supplementary Fig. 1, and Supplementary Tables 3 and 4).

### Mapping genetic mediators of mCG steady-state levels

**Fig. 2:**
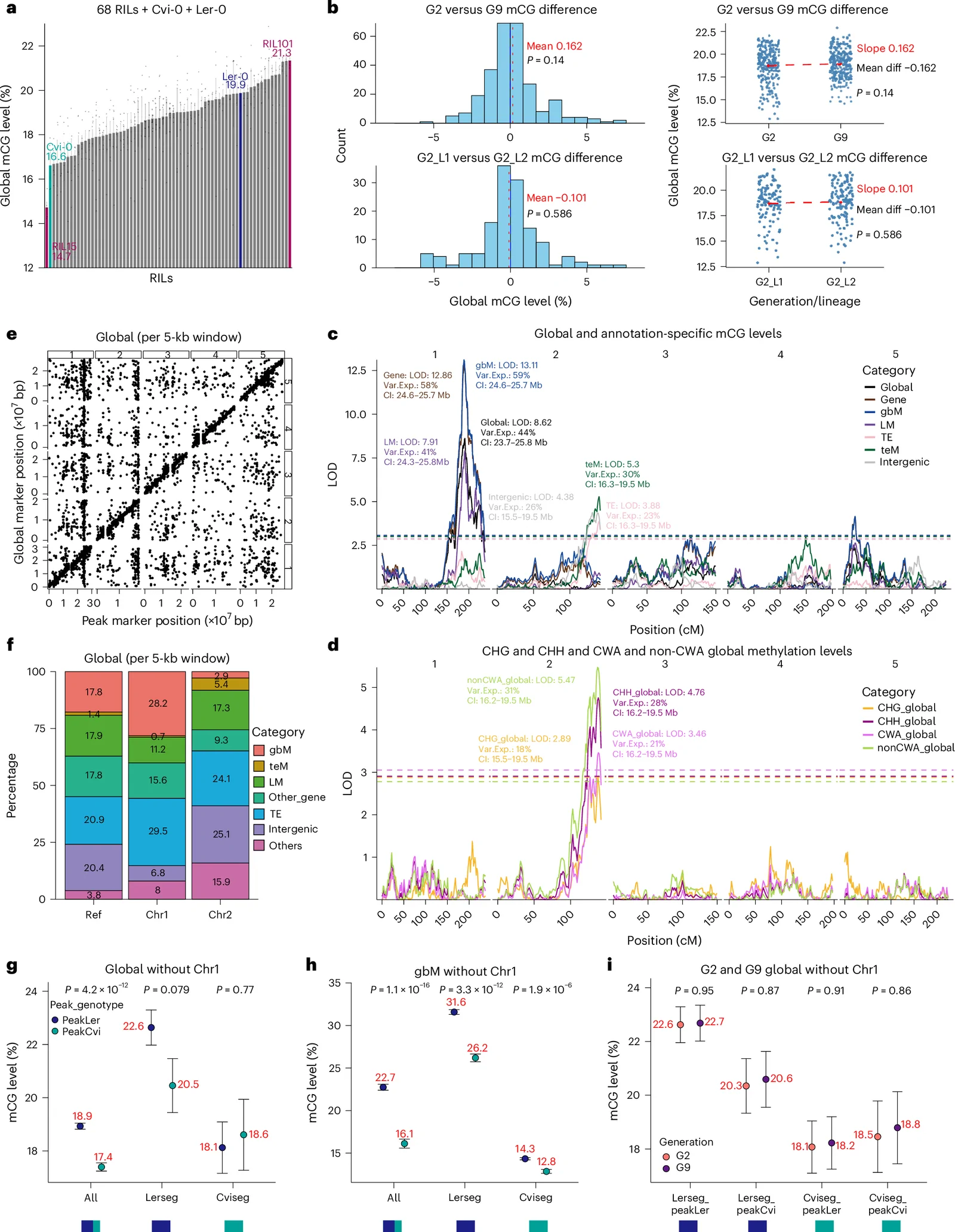
Mapping genetic mediators of methylation levels. **a**, Genome-wide mCG levels across 68 RILs derived from Ler × Cvi and their parental lines. Lines are ranked by the mean mCG level averaged across all samples for each RIL. Dark magenta labels indicate RILs with extreme mCG levels. The parental lines, Ler-0 and Cvi-0, are highlighted in blue and teal, respectively. Bars show mean values, and error bars represent s.e.m. Overlaid dots represent biologically independent WGBS samples derived from individual plants. The exact number of WGBS samples (*n*) for each RIL and parental line is presented in Supplementary Table 2, which lists all 371 biologically independent WGBS samples. **b**, Stability of mCG levels within RILs across generations and lineages. Top: histograms (left) and paired plots (right) show negligible differences between early (G2) and late (G9) generations within each RIL, indicating steady-state methylation levels. Bottom: comparisons between two independent lineages of the same RIL show similarly high concordance in mCG levels. Two-sided paired *t*-tests were used for both comparisons; *P* values are indicated. **c**, meQTL mapping results using global and annotation-specific mCG levels as quantitative traits. A major-effect QTL on chromosome 1 significantly affects global, gene and gbM methylation levels, while an additional locus on chromosome 2 specifically influences methylation in TE, teM and intergenic regions. Peak LOD scores (‘LOD’), explained variance (‘Var.Exp.’) and CIs (‘CI’) are indicated. Horizontal dashed lines represent significance thresholds determined by permutation testing (*α* = 0.05, 1,000 permutations). Position is given in cM. The numbers 1–5 above the graphs indicate chromosomes 1–5, respectively. **d**, QTL mapping for global non-mCG identified a major-effect locus for CHG, CHH, CWA and non-CWA methylation on chromosome 2, colocalizing with the mCG meQTL associated with TE, teM and intergenic regions shown in **c**. **e**, Genome-wide *cis*–*trans* analysis of mCG in 5-kb windows reveals 2 major *trans*-acting QTL bands colocalizing with chromosome 1 and chromosome 2 meQTL. **f**, Genomic feature enrichment within *trans* bands highlights distinct target preferences of chromosome 1 (enriched for gbM genes) and chromosome 2 (enriched for TEs, teM and intergenic regions) meQTL. **g**,**h**, Haplotype-specific and directional effects of the chromosome 1 meQTL on global (**g**) and gbM-specific (**h**) mCG. The Cvi allele at the peak of the chromosome 1 meQTL (PeakCvi) significantly reduces methylation levels in Ler-derived genomic segments (Lerseg), with little effect on Cvi-derived segments (Cviseg). Points show mean values, and error bars represent the s.e.m. *n* = 46 (PeakLer) and *n* = 22 (PeakCvi) independent RILs in each genomic segment (all, Lerseg, Cviseg). *P* values are from two-sided two-sample *t*-tests comparing PeakLer versus PeakCvi, with *P* < 0.05 considered significant. **i**, Global mCG levels in G2 and G9 generations across genotypes stratified by chromosome 1 meQTL haplotype, showing that the genetic effect is established early and remains stable across generations. Points show mean values, and error bars represent the s.e.m. In each generation (G2 and G9), *n* = 46 independent RILs for the PeakLer groups and *n* = 22 for the PeakCvi groups, in both genomic segments (Lerseg and Cviseg). *P* values are from two-sided *t*-tests comparing G2 versus G9, with *P* < 0.05 considered significant. No adjustment for multiple comparisons was applied; the four G2–G9 comparisons were prespecified within-group contrasts and are reported individually. Source data

Genome-wide DNA methylation profiling of MA-ML revealed a 1.4-fold variation in global mCG levels across genotypes, ranging from 14.7% to 21.3%. The parental accessions Ler-0 and Cvi-0 scored towards the extremes of this distribution (Fig. 2a and Supplementary Table 5), consistent with limited transgressive segregation^36^. Comparisons of mCG levels between G2 individuals and their G9 descendants for each genotype revealed no significant differences (Fig. 2b), which suggests that each lineage had reached mCG steady state already at the onset of MA-ML construction. This stability contrasts with the progressive methylation loss typically observed in many DNA methylation mutants^37–39^. On this basis, we pooled genome-wide mCG data from all samples within each pedigree. Treating the mCG steady-state level of each genotype as a quantitative molecular trait, we performed linkage-based meQTL mapping. This analysis identified a single major-effect meQTL on chromosome 1 that accounted for 44% of the variance in global mCG (logarithm of the odds (LOD), 8.62; CI, 23.7–25.8 Mb) (Fig. 2c and Supplementary Tables 5 and 6). To explore whether additional annotation-specific effects were masked in the global analysis, we performed stratified mapping across distinct genomic features. The chromosome 1 meQTL exhibited its strongest effect on mCG levels within gene body-methylated (gbM) genes, explaining approximately 59% of the variance (LOD, 13.11; CI, 24.6–25.7 Mb), and also revealed two minor-effect loci on chromosomes 3 and 5. In addition, a distinct, strong-effect meQTL was detected at the distal end of chromosome 2, specifically influencing mCG levels in TEs, TE-like methylated (teM) genes and intergenic regions, accounting for 23%, 30% and 26% of the variance in these features, respectively (Fig. 2c and Supplementary Tables 5 and 6).

**Extended Data Fig. 1:**
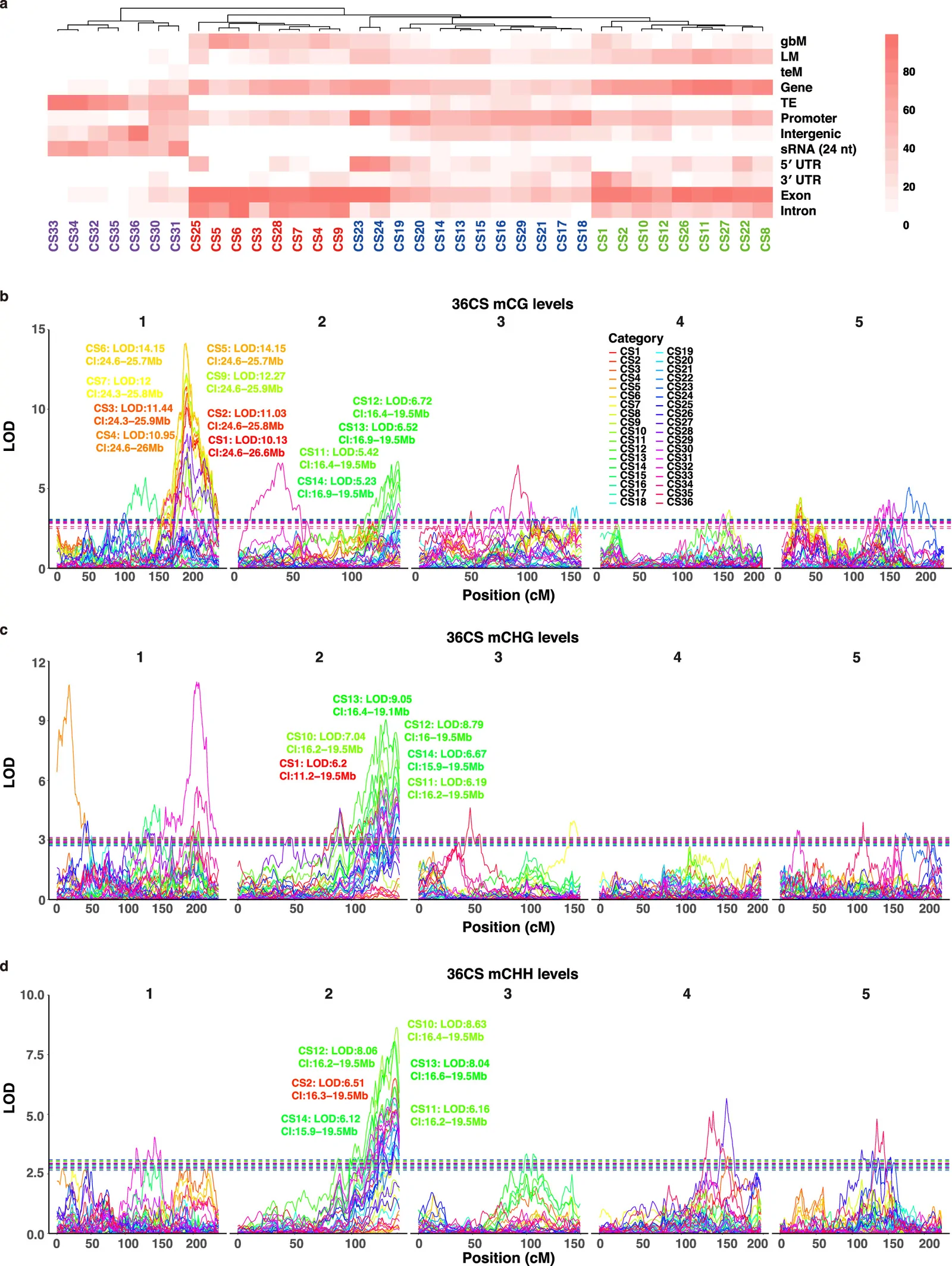
Methylation QTL (meQTL) mapping for 36 chromatin states (CSs) reveals context-specific effects. **a**, Hierarchical clustering of 36 chromatin states (CSs) according to their enrichment for known genomic annotations in *Arabidopsis thaliana*, identifies four main clusters (purple, red, blue, and green). Annotation categories include gene body methylated (gbM), lowly methylated (LM), TE-like methylated (teM), gene, transposable element (TE), promoter, intergenic region, 24-nucleotide (nt) small RNA (sRNA) target, untranslated region (5′ UTR, 3′ UTR), exon, and intron. Color intensity indicates the enrichment within each CS. **b-d**, Genome-wide meQTL mapping for (**b**) CG, (**c**) CHG, and (**d**) CHH methylation levels, across the 36 CSs. Major-effect QTL on chromosome 1 predominantly affect mCG in gbM-associated states, while the chromosome 2 locus specifically influences methylation of TE-associated and intergenic regions across mCG, mCHG, and mCHH contexts. Peak logarithm of the odds (LOD) scores (“LOD”), Confidence Intervals (“CI”), and states exhibiting the strongest associations are highlighted. Horizontal dashed lines represent significance thresholds determined by 1000 permutations (α = 0.05). Position is given in centiMorgans (cM).

Given the frequent coregulation of mCG with non-mCG (mCHG and mCHH) in these later annotation contexts through shared epigenetic pathways^1,40^, we hypothesized that the chromosome 2 meQTL might also affect non-mCG. Supporting this, mapping of global mCHG and mCHH levels identified a major-effect locus that colocalized with the chromosome 2 mCG meQTL. The strongest association was observed for CHH methylation, particularly at non-CWA (CWA refers to CAA or CTA) contexts (Fig. 2d and Supplementary Tables 5 and 6), hinting at an involvement of the RNA-directed DNA methylation (RdDM) pathway. Interestingly, *FBX5* (AT2G44900) emerged as a significant *cis*-eQTL (where, eQTL is expression QTL) target located within the QTL CI in our analysis (Supplementary Table 7), which is consistent with its recent identification by Zicola et al. as a novel modifier of DNA methylation in natural Cvi accessions^41^. The target specificity of the two major meQTL was further supported by repeating the analysis using chromatin state (CS) annotations of the *A. thaliana* genome^18,42^. The chromosome 2 locus was specifically associated with four CSs enriched in intergenic and TE regions, while the chromosome 1 locus was predominantly associated with mCG levels in CS characteristic of gbM subregions^18^ (Extended Data Fig. 1 and Supplementary Table 8).

**Extended Data Fig. 2:**
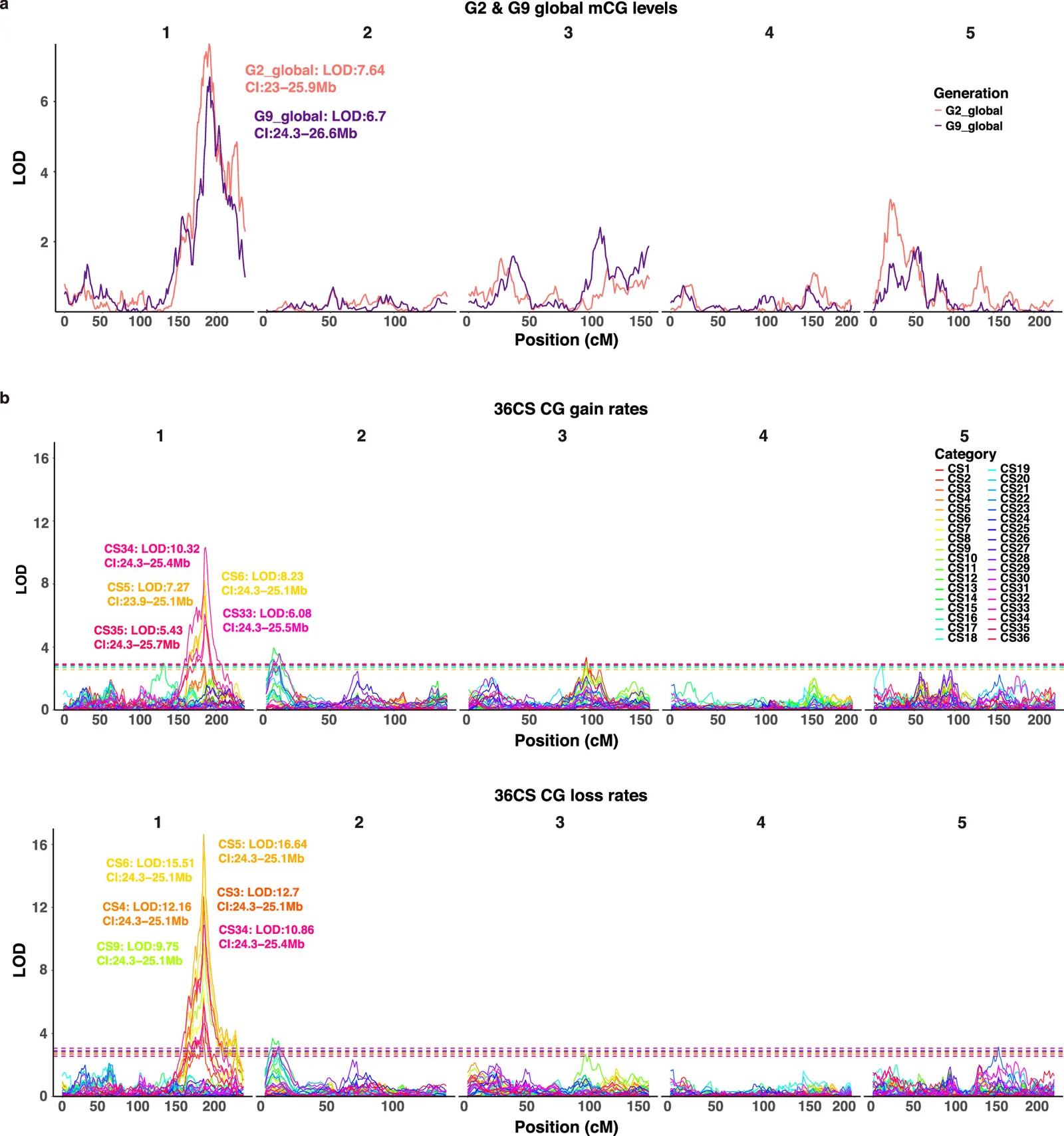
QTL mapping of mCG levels in G2 and G9 generations and epimutation rates across 36 CSs. **a**, meQTL mapping for global mCG levels in generation 2 (G2) and generation 9 (G9) of mutation accumulation pedigrees. Both analyses identify a major-effect QTL on chromosome 1 with similar genomic intervals, indicating early establishment and long-term stability of the underlying genetic effects. **b**, QTL mapping of epimutation rates (gain and loss) across 36 CSs. Top panel illustrates CG methylation gain rates, bottom panel illustrates CG methylation loss rates. A major-effect QTL on chromosome 1 significantly influences both CG gain and loss rates across multiple chromatin states, consistent with the action of a shared genetic modifier. Peak LOD scores and confidence intervals are highlighted for states exhibiting the strongest associations.

The strong effects of the chromosome 1 and chromosome 2 meQTL on both global and feature-specific mCG levels suggested that they act primarily in *trans* to modulate methylation at distal targets. To test this directly, we conducted a genome-wide *cis*–*trans* analysis by segmenting the genome into 5-kb windows and treated regional mCG levels as quantitative traits. This revealed two prominent *trans* bands colocalizing with chromosomes 1 and 2 meQTL (Fig. 2e). As expected, the chromosome 1 *trans* band was enriched for gbM, whereas the chromosome 2 *trans* band was enriched for TEs, teM genes and intergenic regions (Fig. 2f), consistent with prior annotation-stratified results (Fig. 2c and Extended Data Fig. 1). We examined the directionality and haplotype specificity of these *trans*-acting effects in detail. We found that the Cvi allele at the chromosome 1 locus was associated with reduced steady-state mCG at distal targets to levels approximating those of the Cvi-0 parent but only within Ler-derived genomic segments (Fig. 2g,h and Supplementary Table 5). This suggests that the Cvi allele induces hypomethylation primarily at premethylated Ler-inherited loci. To confirm the transgenerational stability of genetic effects on mCG steady-state levels, we independently performed meQTL mapping in the G2 and G9 generations. Both analyses revealed the same genetic architecture underlying the hypomethylation phenotypes, indicating that these effects are established early and persist across generations (Fig. 2i and Extended Data Fig. 2a).

Together, these findings demonstrate that the meQTL elicits rapid and stable transitions to new methylation steady states in MA-ML genomes, primarily by acting in *trans* to reduce methylation on Ler-derived genomic segments.

### A major QTL modulates the spontaneous CG epimutation rates

**Fig. 3:**
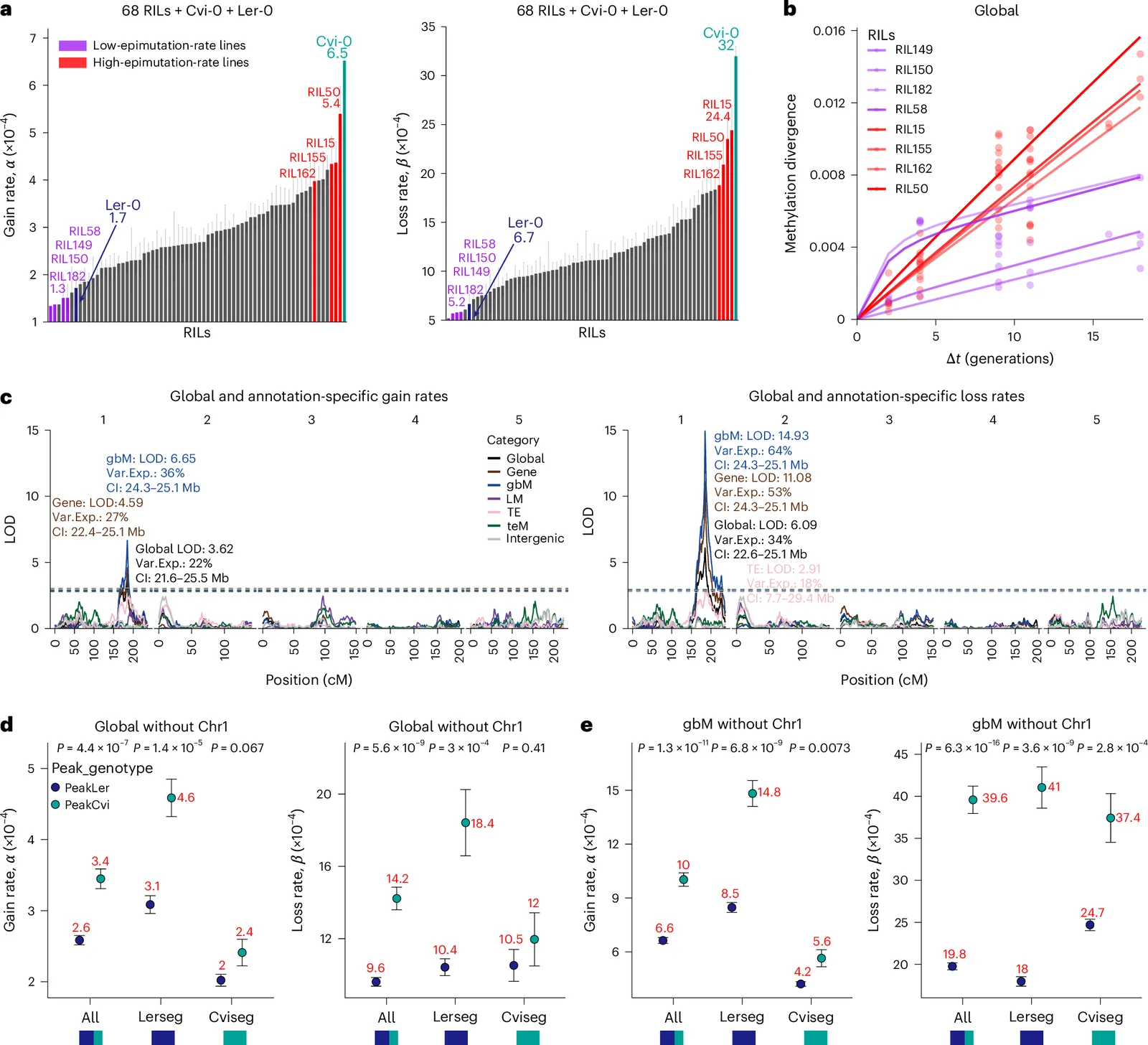
Mapping genetic modifiers of CG epimutation rates. **a**, Distribution of genome-wide estimates of spontaneous CG epimutation gain (left) and loss (right) rates across 68 RILs and their parental lines (Ler-0 and Cvi-0). Lines are ranked by rate; RILs with extreme epimutation rates and parental lines are colour highlighted. Bars represent point estimates of mathematical model parameters for epimutation rates, and error bars denote the s.e. of these estimates. Estimates are based on biologically independent WGBS samples derived from individual plants. The exact number of WGBS samples (*n*) for each RIL and parental line is presented in Supplementary Table 2, which lists all 371 biologically independent WGBS samples. The sample sizes used in epimutation rate estimations are 1/2 × *n* × (*n* *−* 1), where *n* is the number of sequenced samples in each MA pedigree. **b**, Global methylation divergence over multiple generations for selected RILs with extreme epimutation rates, illustrating faster divergence in hyperepimutable lines (red) compared with stable lines (purple). **c**, QTL mapping results for global and annotation-specific spontaneous mCG gain (left) and loss (right) rates. A major-effect locus on chromosome 1 significantly modulates both gain and loss rates, with a notably stronger effect on loss rates, particularly within gbM genes. This locus colocalizes with the previously identified meQTL for steady-state mCG levels. meQTL for methylation levels identified on chromosomes 2, 3 and 5 were not associated with epimutation rate variation. Peak LOD scores (‘LOD’), explained variance (‘Var.Exp.’) and CIs (‘CI’) are indicated. Horizontal dashed lines represent significance thresholds determined by permutation testing (*α* = 0.05, 1,000 permutations). Position is given in cM. The numbers 1–5 above the graphs indicate chromosomes 1–5, respectively. **d**,**e**, Haplotype-specific effects of the chromosome 1 QTL on global (**d**) and gbM-specific (**e**) epimutation rates. The Cvi allele (PeakCvi) increases both gain and loss rates predominantly in Ler-derived genomic regions (Lerseg), with minimal effects in Cvi-derived regions (Cviseg). Points show mean values and error bars represent the s.e.m. Sample sizes are *n* = 46 independent RILs for the PeakLer groups and *n* = 22 independent RILs for the PeakCvi groups in each genomic segment (all, Lerseg and Cviseg). *P* values are from two-sided two-sample *t*-tests comparing PeakLer versus PeakCvi, with *P* < 0.05 considered significant.

We asked whether the genetically induced shifts in steady-state mCG are accompanied by secondary changes in the transgenerational dynamics of CG maintenance; that is, whether perturbations that reset the equilibrium also increase maintenance flux and reduce transmission fidelity across generations. Prior work in *cmt3* mutants showed that even modest perturbations of genome-wide methylation can destabilize epigenetic inheritance, increasing the accumulation of spontaneous epimutations over successive generations despite apparently robust steady-state levels^18^ (Methods). To test this in the MA-ML population, we estimated genotype-specific epimutation rates across all pedigrees using established tools (Methods). Rates varied widely among RILs, with the most hyperepimutable line reaching 14.5 × 10^−4^ and the most stable line 3.3 × 10^−4^ per site per haploid genome per generation, a 4.4-fold difference in epigenomic fidelity (Fig. 3a and Supplementary Table 9). These differences were mirrored in the rate of mCG divergence over generations, with hyperepimutable lines showing markedly faster epigenetic drift (Fig. 3b). These results align with findings from our MA lines in natural accessions (Fig. 1c,d) and confirm that the fidelity of mCG maintenance is inherently genotype dependent.

To dissect the genetic basis of this variability, we treated genotype-specific epimutation rates as molecular traits and performed QTL mapping. This analysis identified a single major-effect locus on chromosome 1 that influenced both gain and loss rates. The QTL explained 22% of the variance in global CG gain rates and 34% in loss rates, with particularly pronounced effects in gbM genes, where it accounted for 36% and 64% of the variance, respectively (Fig. 3c, Extended Data Fig. 2b and Supplementary Tables 9–11). This locus precisely colocalized with the previously mapped QTL for steady-state mCG levels. By contrast, the minor-effect QTL on chromosomes 3 and 5, as well as the major chromosome 2 locus associated with non-mCG (CHG/CHH) and TE/intergenic mCG, was not associated with variation in epimutation rates (Figs. 2c–f and 3c, Extended Data Figs. 1 and 2, and Supplementary Tables 6 and 8–11). We previously showed that CG sites within or proximal to regions targeted by RdDM are less prone to accumulate epimutations^18,22^, likely owing to periodic reinforcement during gametogenesis and early embryogenesis^43^. Thus, QTLs that co-segregate with mCG levels do not necessarily influence transgenerational maintenance fidelity, indicating that these are distinct molecular phenotypes with partly non-overlapping genetic architectures.

**Extended Data Fig. 3:**
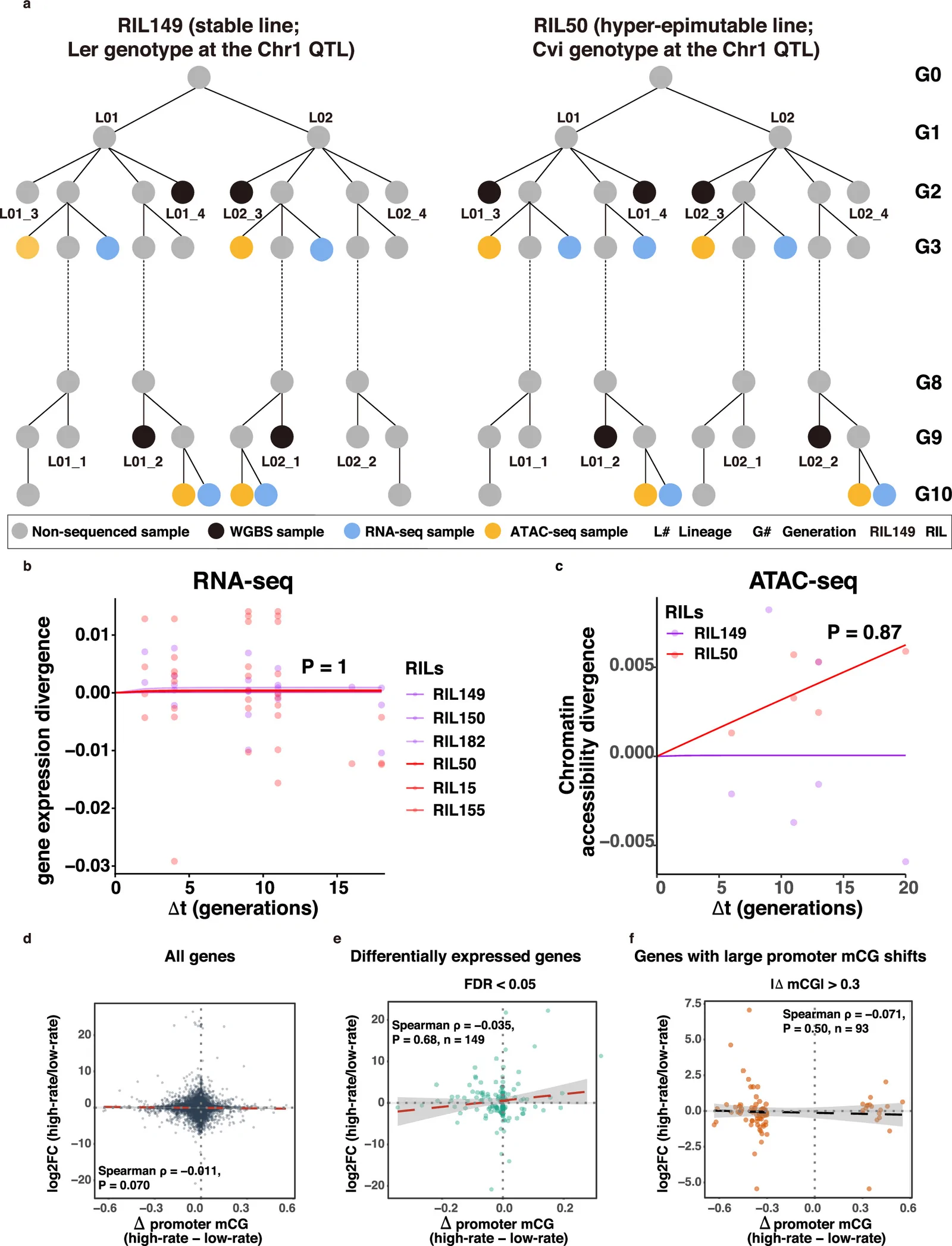
Enhanced epigenetic drift and promoter methylation variation are not associated with measurable regulatory divergence. **a**, Pedigrees and sample structure for RNA-seq and ATAC-seq data. MA pedigrees for two RILs with contrasting epimutation rates—RIL50 (hyper-epimutable, Cvi genotype at Chr1 QTL) and RIL149 (stable, Ler genotype). Blue circles indicate RNA-seq samples and orange circles indicate ATAC-seq samples, both collected from the immediate progeny of sibling plants used for WGBS (black circles), ensuring temporal and genetic continuity between gene expression, chromatin accessibility, and methylation measurements. RNA-seq data were generated for six RILs with contrasting epimutation rates; pedigrees for RIL149 and RIL50 are shown as representative examples. ATAC-seq data were generated for RIL149 and RIL50. **b**, Gene expression divergence (measured via RNA-seq) as a function of generational time (Δt, generations) across six Ler × Cvi RILs with contrasting epimutation rates. Neither high-rate nor low-rate lines showed significant accumulation of gene expression over generations (two-sided F-test, *P* = 1). Lines represent model fits for each RIL. **c**, Chromatin accessibility divergence (measured via ATAC-seq) across generations for RIL149 and RIL50. Similar to gene expression, no significant accumulation of chromatin accessibility divergence was detected over time (two-sided F-test, *P* = 0.87). Lines represent model fits. **d–f**, Targeted promoter-level analysis comparing expression changes (log2 fold change between high-rate and low-rate lines) against differences in promoter mCG levels (Δpromoter mCG between high-rate and low-rate lines). Scatter plots are shown for all genes (**d**), differentially expressed genes (FDR < 0.05) (**e**), and genes exhibiting large promoter methylation shifts (|ΔmCG| > 0.3) (**f**). The dashed lines represent the fitted linear regression (predicted mean values), and the grey shaded areas indicate the 95% confidence intervals. Spearman correlation (ρ) and *P* values from a two-sided Spearman rank correlation test indicate that variation in promoter methylation is not correlated with transcriptome variation across genes. Each point represents one gene. For (**e**) and (**f**), “n” represents the number of genes.

To explore the haplotype specificity of these QTL effects, we examined epimutation patterns in Ler- and Cvi-derived chromosomal segments in more detail. We found that the elevated loss rates associated with the Cvi allele at the chromosome 1 QTL were largely restricted to Ler-derived genomic regions (Fig. 3d,e and Supplementary Table 9), consistent with earlier findings that the Cvi allele drives hypomethylation of these segments (Fig. 2g,h and Supplementary Table 5). Interestingly, however, Ler segments also displayed a higher propensity for methylation gain in the presence of the Cvi allele (Fig. 3d,e and Supplementary Table 9), pointing to a more general destabilization of mCG maintenance in these segments across generations. We reasoned that increased epigenetic drift might also promote downstream functional divergence over time. To test this directly, we generated RNA sequencing (RNA-seq) and assay for transposase-accessible chromatin using sequencing (ATAC-seq) data across multiple generations of MA-ML pedigrees with contrasting spontaneous CG epimutation rates (Extended Data Fig. 3a, Supplementary Tables 12 and 13, and Methods). Across gene expression and chromatin accessibility, neither the high-rate lines carrying the Cvi genotype at the chromosome 1 QTL nor the low-rate lines showed significant functional divergence over generations (RNA-seq: *P* = 1; *F*-test; ATAC-seq: *P* = 0.87) (Extended Data Fig. 3b,c and Methods). This conclusion was further supported by a more targeted promoter-level analysis, which revealed that variation in promoter methylation was not correlated with transcriptome variation across samples (Extended Data Fig. 3d–f). Thus, the enhanced methylation drift does not translate into measurable regulatory divergence in these data (Extended Data Fig. 3). This observation is in line with previous work showing that spontaneous CG epimutations accumulate in a selectively neutral manner^17,19,22^. It is also consistent with evidence that natural and experimental perturbations of gbM, the primary genomic target of the QTL, often have limited^44^ or no detectable^45^ functional consequences.

Taken together, our findings support a model in which the Cvi-derived chromosome 1 haplotype not only shifts mCG to a lower steady state but also renders this state intrinsically less stable across generations. Over the timescale assayed here, the cumulative gains and losses of mCG remain largely buffered from measurable changes in gene expression and chromatin accessibility at the genome-wide scale.

### A chromosome 1 QTL harbours a large deletion associated with *VIM2*/*4* overexpression

**Fig. 4:**
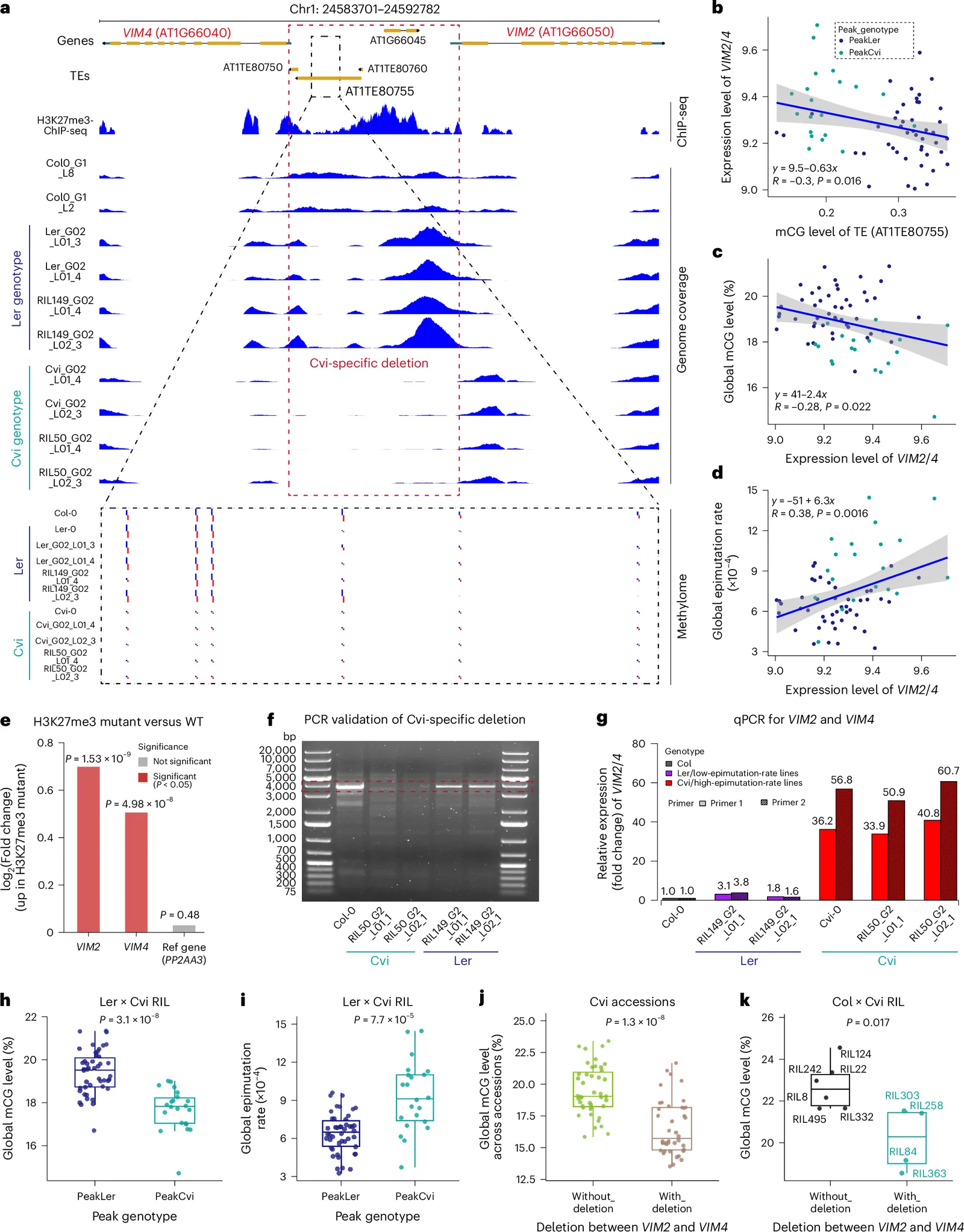
A Cvi-specific deletion between *VIM2* and *VIM4* reduces mCG levels and elevates epimutation rates through enhanced *VIM2*/*4* expression. **a**, Genomic view of the *VIM2*/*4* region. The top track shows H3K27me3 ChIP-seq enrichment in Col-0. The middle and bottom tracks show genome coverage and DNA methylation, respectively, for the Col-0, Ler-0 and Cvi-0 accessions and for two Ler × Cvi RILs with contrasting epimutation rates: RIL50, a hyperepimutable line carrying the Cvi genotype at the chromosome 1 QTL, and RIL149, an epigenetically stable line carrying the Ler genotype at the same locus. For each RIL, two biological replicates are shown for both genome coverage and DNA methylation. An approximately 2.7-kb Cvi-specific deletion (dashed red box) between *VIM2* and *VIM4* removes 3 TEs (AT1TE80750, AT1TE80755 and AT1TE80760), including a strongly methylated ATREP3 element (AT1TE80755; dashed black box) in Col-0 and the Ler haplotype. **b**–**d**, Relationships between combined *VIM2* and *VIM4* (that is, *VIM2*/*4*) expression, TE methylation level, global mCG level and epimutation rates across RILs. *VIM2*/*4* expression is significantly negatively correlated with mCG level at AT1TE80755 (**b**) and with global mCG levels (**c**) and positively correlated with global CG epimutation rates (calculated as 0.5 × (*α* + *β*), where *α* is the gain rate and *β* is the loss rate) (**d**). Solid blue lines show the linear regression fit (the centre of the band, representing the predicted mean), and shaded areas represent the 95% CI for the predicted mean. Statistics are Pearson’s correlation coefficient (*R*) and the *P* value from a two-sided Pearson correlation test. Two extreme *VIM2*/*4* expression data points were outliers and removed for linear regression fitting. **e**, Expression changes of *VIM2*, *VIM4* and reference housekeeping gene *PP2AA3* (AT1G13320) in an H3K27me3-pathway mutant relative to wild type. Bars show log_2_ fold changes; significant changes are indicated in red and non-significant changes in grey. *P* values are from two-sided Wald tests implemented in DESeq2. **f**, PCR validation of the Cvi-specific deletion in selected RILs confirms genotype-specific segregation. This experiment was repeated independently four times with similar results. **g**, qPCR-based quantification of *VIM2* and *VIM4* expression in selected RILs shows elevated expression in lines carrying the Cvi-specific deletion. Primer 1 amplifies *VIM2* only, while primer 2 amplifies *VIM2* and *VIM4*. The difference between primer 2 and primer 1 therefore reflects *VIM4* expression, indicating that both *VIM2* and *VIM4* are individually upregulated in deletion-carrying lines. Expression is normalized to Col-0 as a reference. Lines carrying the Cvi-specific deletion (red; high epimutation rate) show obvious elevated expression levels compared with Ler genotype lines (purple; low epimutation rate) and Col-0 reference (grey). **h**,**i**, Global mCG levels (**h**) and global CG epimutation rates (**i**) in Ler × Cvi RILs grouped by genotype at the chromosome 1 QTL peak (PeakLer versus PeakCvi). *n* = 46 (PeakLer) and *n* = 22 (PeakCvi) independent RILs. **j**, Global mCG levels across natural Cvi accessions stratified by the presence or absence of the deletion between *VIM2* and *VIM4*. *n* = 43 (without_deletion) and *n* = 40 (with_deletion) independent accessions. **k**, Genome-wide mCG levels in an independent Col × Cvi RIL population stratified by the presence or absence of the *VIM2*/*4* deletion. Individual RIL identifiers are labelled. *n* = 6 (without_deletion) and *n* = 4 (with_deletion) independent RILs. For box plots (**h**–**k**), centre lines indicate the median, box limits represent the upper and lower quartiles, whiskers extend to 1.5× the interquartile range, and individual dots represent data points. Significance was calculated by two-sided *t*-tests with *P* values indicated. Source data

To identify the mechanistic basis of the chromosome 1 QTL, we searched for candidate genes by performing eQTL mapping using previously generated transcriptome data from the Ler × Cvi RIL population^34,35^. This analysis identified *VIM2*/*4* (combined expression of *VIM2* and *VIM4*) as a significant *cis*-eQTL target within the QTL CI (Fig. 4a and Supplementary Tables 14 and 15). The *VIM* gene family encodes proteins that recruit MET1 to hemimethylated DNA templates during replication, thereby promoting restoration of mCG on the newly synthesized strand^1,8–10^. Public expression atlases demonstrate that *VIM2*/*4* are robustly expressed across diverse tissues and developmental stages^46,47^ (Supplementary Fig. 2), consistent with a systemic role in methylation maintenance.

**Extended Data Fig. 4:**
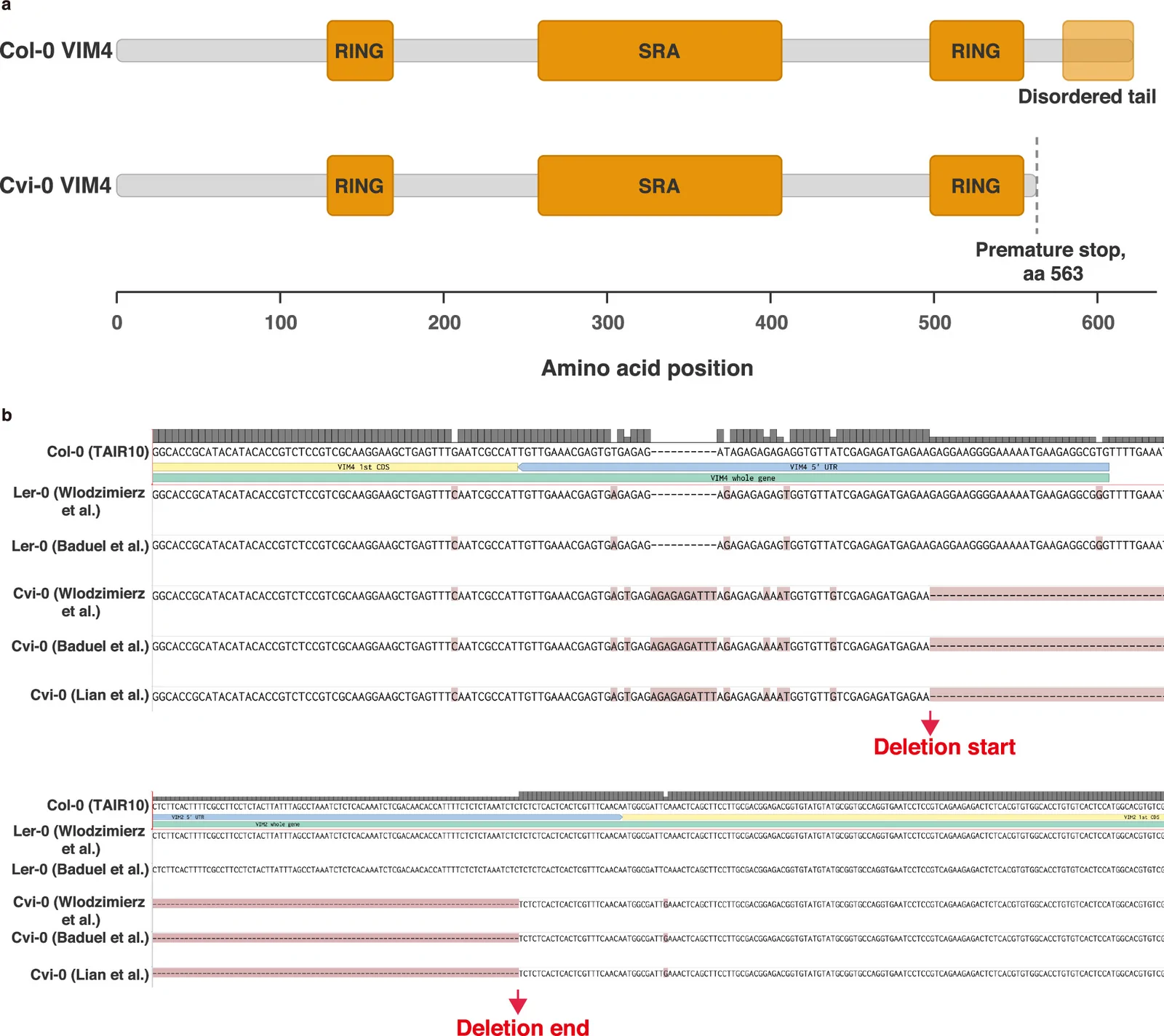
Domain architecture of *VIM4* and validation of the Cvi-specific deletion using long-read assemblies. **a**, Predicted domain architecture of *VIM4* and position of the Cvi-specific premature stop codon. Schematic representation of the *Arabidopsis VIM4* protein in Col-0 and Cvi-0, showing the canonical N-terminal RING domain (129-169 amino acids (aa)), the central SRA domain (258-407 aa), the C-terminal RING domain (498-555 aa), and the disordered C-terminal tail (579-622 aa), according to UniProt/InterPro annotation (UniProtKB A0A7G2E6T4). In Cvi-0, a premature stop codon at residue 563 lies downstream of the C-terminal RING domain and truncates only the predicted disordered C-terminal tail, while all annotated domains remain intact. Because the stop codon is located approximately 9 nucleotides upstream of the last exon-exon junction, within the canonical nonsense-mediated mRNA decay (NMD) escape window, the transcript is predicted to evade NMD and produce a nearly full-length protein retaining hemimethylated CG recognition through its SRA domain. **b**, Alignments of genomic sequences across multiple independent long-read assemblies for Col-0 (TAIR10), Ler-0 (Wlodzimierz et al.^49^; Baduel et al.^48^), and Cvi-0 (Wlodzimierz et al.^49^; Baduel et al.^48^; Lian et al.^50^) define the precise start and end breakpoints of the approximately 2.7-kb Cvi-specific deletion between *VIM2* and *VIM4*. Red arrows mark the precise deletion start and end positions of the Cvi-specific deletion. Annotated sequence features of *VIM2* and *VIM4* from the Col-0 reference (TAIR10) are shown above the alignment. All three independent Cvi-0 assemblies consistently confirm the presence of a ~2.7 kb deletion in the intergenic region between *VIM2* and *VIM4*, whereas Col-0 and Ler-0 assemblies retain the full-length sequence.

*VIM4* lies approximately 2.6-kb downstream of *VIM2* (Fig. 4a) and, as a paralogue, shares strong sequence and structural similarity with *VIM2*, suggesting possible coregulation. Re-analysis of published long-read assemblies for Ler-0 and Cvi-0^48–50^, together with the Col-0 reference, showed that the coding and protein sequences of *VIM2* and *VIM4* are >99% identical across all three accessions (Supplementary Tables 16 and 17). In Col-0, the two paralogues differ by only five amino acid residues and in Ler-0 by four residues. In Cvi-0, the VIM4 protein is identical to VIM2 except for a 59-amino-acid C-terminal truncation caused by a premature stop codon at position 563 (ref. ^41^) (Extended Data Fig. 4a and Supplementary Tables 16 and 18). As this stop codon lies only approximately nine nucleotides upstream of the final exon–exon junction, within the canonical escape window for nonsense-mediated decay (NMD), the transcript is likely to evade degradation. Moreover, domain mapping (Extended Data Fig. 4a) indicated that the truncation leaves all major functional domains intact, including the N-terminal RING, SRA and C-terminal RING domains. The truncated Cvi VIM4 protein is therefore predicted to retain mCG-binding activity through its SRA domain, suggesting that it remains functional.

A recent genome-wide association studies of natural Cvi accessions from the Cape Verde Islands likewise identified a strong association with mCG variation at this locus^41^. Our read-coverage analysis revealed a Cvi-specific deletion of approximately 2.7 kb between *VIM2* and *VIM4*, which segregates both in the MA-ML population (Fig. 4a) and among natural Cvi accessions^41^. By leveraging published long-read assemblies of Cvi-0 from multiple independent sources^48–50^, we confirmed the presence of this deletion in the Cvi-0 accession (Extended Data Fig. 4b). A broader survey of published long-read assemblies across *Arabidopsis* accessions did not detect this deletion outside the Cvi lineage^50–52^, including in accessions phylogenetically or geographically close to Cvi-0 (Can-0, Elh-2 and Toufl-1)^50,53^, consistent with a relatively recent, Cvi-lineage-specific polymorphism.

The deleted interval spans three small TEs: AT1TE80750 (~100 base pairs (bp); ATREP15), AT1TE80755 (~1 kb; ATREP3) and AT1TE80760 (~20 bp; ATREP15). All three belong to the RC/Helitron family. Among them, AT1TE80755 is strongly CG methylated in the reference accessions Col-0 and Ler-0 (Fig. 4a,b), consistent with a repressive chromatin environment at this locus. RILs carrying the Cvi haplotype, which lacks these elements, showed significantly elevated *VIM2*/*4* expression relative to lines carrying the intact Ler haplotype (Fig. 4a–d and Supplementary Table 14). Published chromatin data further showed strong Polycomb-associated H3K27me3 enrichment in the same deleted interval, proximal to or overlapping *VIM2*/*4*^54^ (Fig. 4a). Re-analysis of H3K27me3-pathway mutants supports a functional contribution of this mark, as reduction of H3K27me3 in the region is accompanied by significantly elevated *VIM2* and *VIM4* expression^55^ (Fig. 4e). Together, these results point to a local repressive module within the deleted interval. Both the methylated Helitron AT1TE80755 and the associated H3K27me3 domain may contribute to repression of *VIM2* and *VIM4*. The Cvi deletion therefore likely derepresses both genes by removing both features at once.

**Extended Data Fig. 5:**
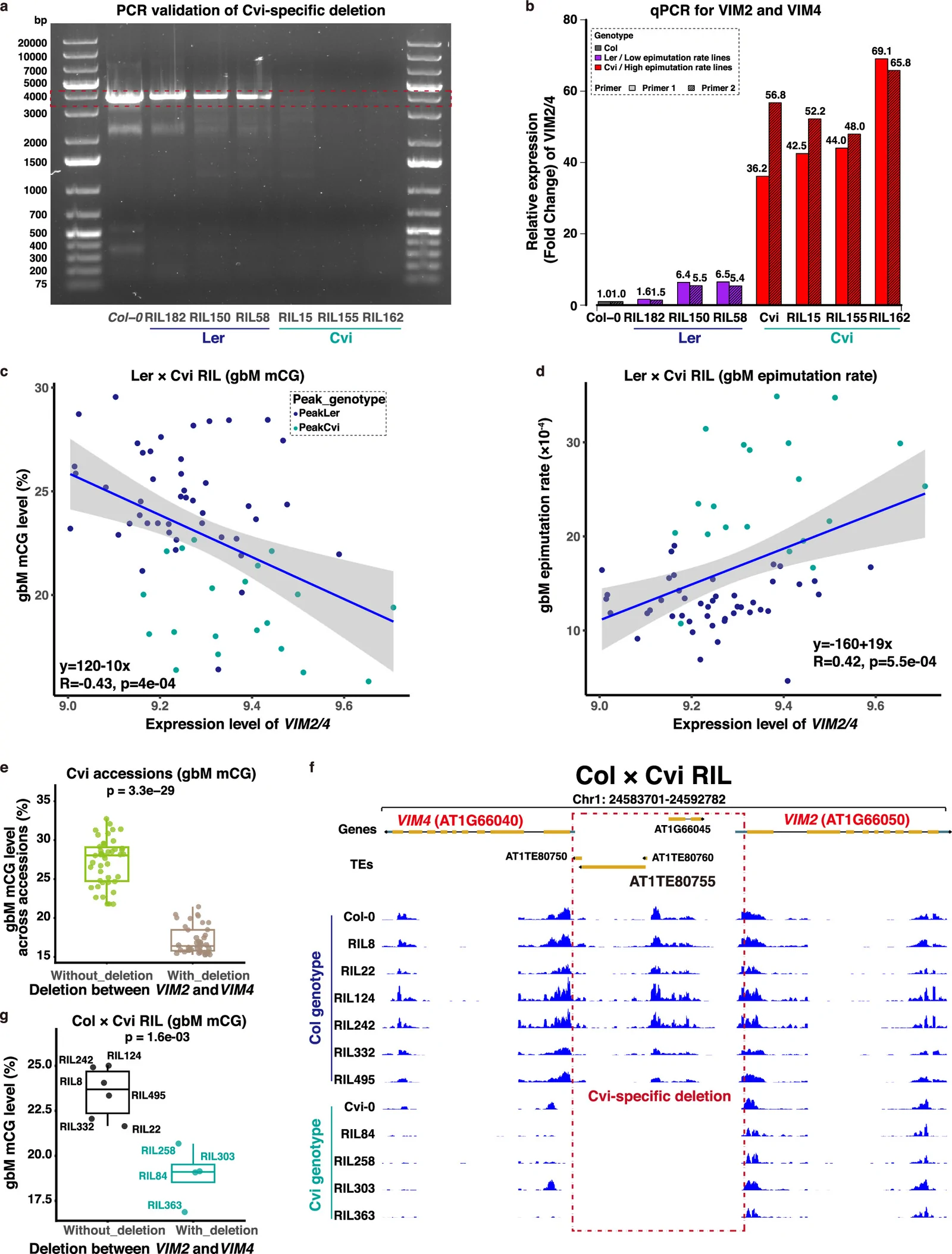
The Cvi-specific deletion elevates *VIM2/4* expression, reducing mCG levels across multiple genetic backgrounds, and increasing epimutation rates in Ler × Cvi RILs. **a**, PCR validation confirming genotype-specific segregation of the Cvi-specific deletion in representative RILs. This experiment was repeated independently four times with similar results. **b**, qPCR-based quantification of *VIM2* and *VIM4* expression in selected RILs shows elevated expression in lines carrying the Cvi-specific deletion. Primer 1 amplifies *VIM2* only, while Primer 2 amplifies *VIM2* and *VIM4*. The difference between Primer 2 and Primer 1 therefore reflects *VIM4* expression, indicating that both *VIM2* and *VIM4* are individually upregulated in deletion-carrying lines. Expression is normalized to Col-0 as a reference. **c,d**, Relationships between *VIM2/4* expression and gbM mCG levels (**c**) and gbM CG epimutation rates (calculated as 0.5×(α+β); α, gain rate; β, loss rate) (**d**) across Ler × Cvi RILs. Solid blue lines show the linear regression fit (predicted mean), and shaded areas represent the 95% confidence interval. Statistics are Pearson’s correlation coefficient (R) and the P value from a two-sided Pearson correlation test. Two extreme *VIM2/4* expression data points were outliers and removed for linear regression fitting. **e**, gbM mCG levels across natural Cvi accessions stratified by presence or absence of the deletion between *VIM2* and *VIM4*. n = 43 (without deletion) and 40 (with deletion) independent accessions. **f**, Read-coverage visualization confirming the segregation of the Cvi-specific deletion between *VIM2* and *VIM4* in an independent Col × Cvi RIL population. **g**, gbM mCG levels in an independent Col × Cvi RIL population stratified by the presence or absence of the *VIM2/4* deletion. Individual RIL identifiers are labeled. n = 6 (without deletion) and 4 (with deletion) independent RILs. For box plots (**e, g**), center lines indicate the median, box limits represent the upper and lower quartiles, whiskers extend to 1.5× the interquartile range, and individual dots represent data points. P values were calculated by two-sided t-tests. Source data

To validate this structural variant directly in the MA-ML population, we performed PCR genotyping in selected RILs and confirmed genotype-specific segregation of the deletion (Fig. 4f and Extended Data Fig. 5a). We then quantified the individual expression levels of *VIM2* and *VIM4* by quantitative PCR (qPCR) and observed consistent upregulation of both genes in deletion-containing lines (Fig. 4g, Extended Data Fig. 5b and Methods). Notably, *VIM2*/*4* expression was highly predictive of both mCG levels and spontaneous epimutation rates in the genome as a whole and within gbM regions (Fig. 4c,d, Extended Data Fig. 5c,d, and Supplementary Tables 5, 9 and 14), indicating a strong functional impact on epigenomic stability. On average, the Cvi-specific deletion reduced global mCG levels by 1.7 percentage points (~9% relative reduction) and gbM mCG levels by 5.5 percentage points (~22% relative reduction) and was associated with an approximately 1.5-fold increase in genome-wide epimutation rates and an approximately 1.9-fold increase within gbM regions (Fig. 4c,d,h,i, Extended Data Fig. 5c,d, and Supplementary Tables 5, 9 and 14).

To test whether this effect extends beyond the Ler × Cvi RIL, we re-analysed published methylome data from natural Cvi accessions^41^ and an independent Col × Cvi RIL population^56^. The Cvi deletion was again associated with reduced global DNA methylation in these systems (Fig. 4j,k, Extended Data Fig. 5e–g, Supplementary Fig. 3 and Supplementary Table 19), demonstrating that deletion-driven *VIM2*/*4* overexpression produces hypomethylation regardless of the genetic background.

### *VIM2*/*4* act as dosage-sensitive negative regulators of mCG maintenance

As VIM proteins are canonical regulators of mCG maintenance that recognize hemimethylated DNA and recruit MET1 during replication, elevated *VIM2*/*4* expression in the Cvi background would be expected to enhance maintenance fidelity and promote CG hypermethylation. However, our haplotype-specific QTL effects suggested the opposite. To test directly whether increased *VIM2*/*4* expression instead causes global CG hypomethylation, we generated transgenic lines overexpressing *VIM2*, *VIM4* or *VIM24* (both genes together) in Col-0 (Supplementary Table 20 and Methods). To distinguish dosage-dependent from allele-specific effects, we generated constructs carrying either the Col-0 or Cvi-0 allele of *VIM2*/*4*.

**Fig. 5:**
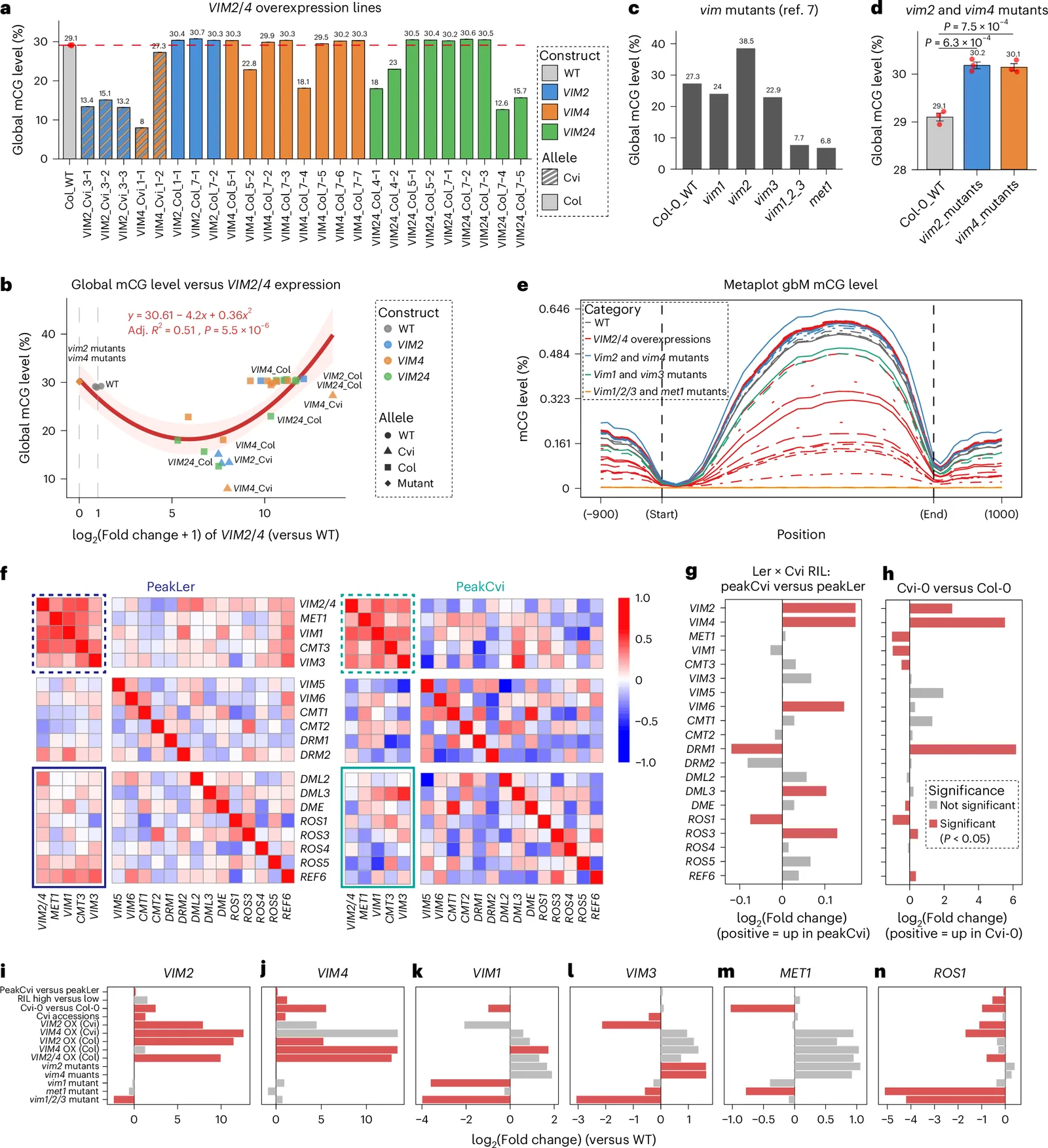
*VIM2*/*4* act as dosage-sensitive negative regulators of mCG maintenance and their dysregulation perturbs DNA methylation pathways. **a**, Genome-wide mCG levels in independent *VIM2*/*4* overexpression lines. Transgenic lines overexpressing *VIM2*, *VIM4* or both paralogues (*VIM24*) were generated in the Col-0 background using Col-0 or Cvi-0 alleles. Bars represent individual transgenic lines. Colours denote construct type. Fill patterns indicate allelic origin. Dashed red lines indicate the mean methylation level of the untransformed Col-0 wild-type control grown under the same conditions, denoted as ‘Col_WT’. **b**, Relationship between global mCG level and *VIM2*/*4* expression across wild type, *VIM2*/*4* overexpression lines (**a**) and *vim2* and *vim4* single mutants (**d**). *VIM2*/*4* expression is shown as log_2_(fold change + 1) relative to wild type. The fitted quadratic regression reveals a nonlinear dosage relationship between global mCG level and *VIM2*/*4* expression: moderate overexpression produces the strongest CG hypomethylation, whereas mCG is retained at extremely high expression levels (>1,000-fold increased relative to wild type). Each point represents 1 independent transgenic line, mutant line or wild-type control (*n* = 34 in total), coloured by construct type and shaped by allele or mutant background. The solid line shows the fitted quadratic regression, and the shaded band represents its 95% CI. Statistical significance of the regression was assessed by a two-sided *F*-test of overall model significance (*F*(2, 31) = 18.37, *P* = 5.47 × 10^−6^); the effect size is given by the adjusted *R*^2^ = 0.51. No adjustment for multiple comparisons was applied because only one overall regression test was performed. Vertical dashed grey lines indicate the mutant and wild-type reference values at *x* = 0 and *x* = 1, respectively. **c**, Genome-wide mCG levels in published *vim* and *met1* mutant methylomes from Stroud et al.^7^. **d**, Genome-wide mCG levels in newly generated *vim2* and *vim4* loss-of-function mutants. Dots represent biological replicates (*n* = 3 per genotype). Bars show mean values and error bars represent s.e.m. *P* values were calculated using two-sided *t*-tests relative to Col-0 wild type. **e**, Metaplot of mCG levels across gbM loci for wild type, *VIM2*/*4* overexpression lines, *vim2* and *vim4* single mutants, *vim1* and *vim3* single mutants, and *vim1/2/3* triple and *met1* mutants. **f**, Gene co-expression correlation network for key methylation pathway genes, comparing RILs with Ler-derived (PeakLer, left) and Cvi-derived (PeakCvi, right) alleles at the chromosome 1 QTL. The dashed box highlights strong co-expression between *VIM2*/*4* and core components of the mCG maintenance machinery, including *MET1*, *VIM1* and *VIM3*. The solid box indicates altered co-expression between methylation maintenance genes and DNA demethylases, reflecting network-level dysregulation between methylation and demethylation pathways in the Cvi genotype. Heat map values represent Pearson’s correlation coefficients. **g**,**h**, Expression changes of DNA methylation regulators in two independent systems. Log_2_ fold changes between Ler × Cvi RILs carrying Cvi-derived (PeakCvi) versus Ler-derived (PeakLer) alleles at the chromosome 1 QTL (**g**); log_2_ fold changes between Cvi-0 and Col-0 accessions based on published transcriptome data (**h**). In both panels, positive values indicate higher expression in the Cvi-associated genotype, and genes with significant differences are highlighted in red (*P* < 0.05). *P* values are from two-sided Wald tests implemented in DESeq2. In **g**, *VIM2* and *VIM4* values reflect the same combined expression estimate, as the two paralogues cannot be reliably distinguished owing to high sequence similarity in the RIL eQTL dataset (Methods). **i**–**n**, Per-gene expression changes of six key methylation regulators across multiple experimental contexts: *VIM2* (**i**), *VIM4* (**j**), *VIM1* (**k**), *VIM3* (**l**), *MET1* (**m**) and *ROS1* (**n**). Bars show log_2_ fold changes relative to wild type (for overexpression and mutant lines) or between indicated contrasts (for RIL and accession comparisons). Datasets include Ler × Cvi RILs (PeakCvi versus PeakLer), RILs with high versus low epimutation rates, Cvi-0 versus Col-0, natural Cvi accessions stratified by the presence versus absence of the Cvi-specific deletion, *VIM2*/*4* overexpression (OX) lines (Cvi and Col alleles), and *vim2*, *vim4*, *vim1*, *met1* and *vim1/2/3* loss-of-function mutants. Significant differences (*P* < 0.05, two-sided Wald tests via DESeq2) are highlighted in red. WT, wild type. Source data

**Extended Data Fig. 6:**
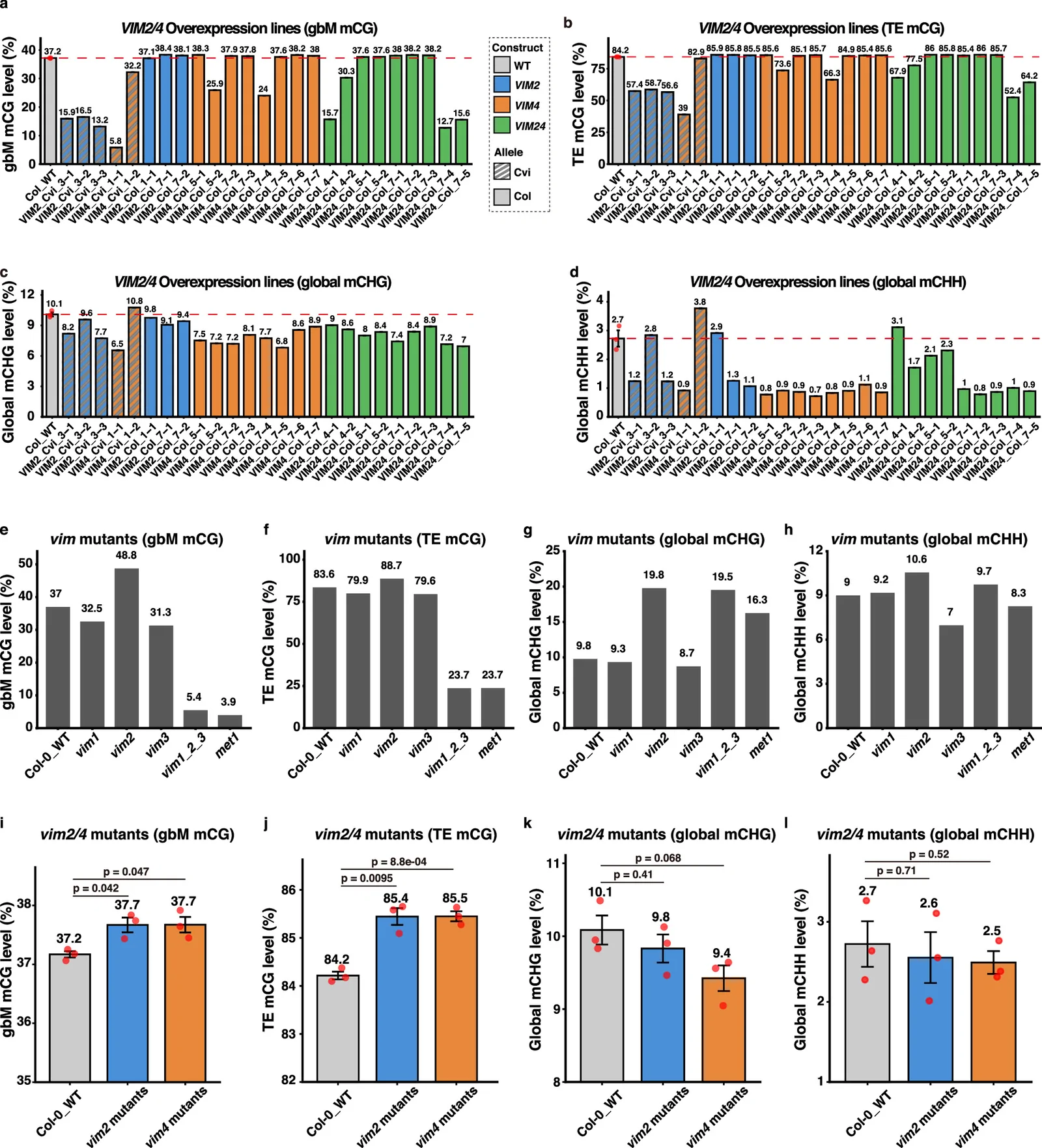
VIM2/4 dosage perturbation has reciprocal effects on CG methylation in overexpression and loss-of-function backgrounds. **a–d**, Annotation-specific and context-specific methylation levels in independent *VIM2/4* overexpression lines generated in the Col-0 background. Transgenic lines overexpressing *VIM2*, *VIM4*, or both paralogues (*VIM24*) were generated in the Col-0 background using Col-0 or Cvi-0 alleles. Methylation levels are shown for gbM mCG (**a**), TE mCG (**b**), global mCHG (**c**) and global mCHH (**d**). Bars represent individual transgenic lines. Colors denote construct type. Fill patterns indicate allelic origin. Dashed red lines indicate the mean methylation level of the untransformed Col-0 wild-type control grown under the same conditions, denoted as “Col_WT”. **e–h**, DNA methylation levels in published *vim* mutant backgrounds (*vim1*, *vim2*, *vim3*, *vim1/2/3*) and the *met1* mutant compared with Col-0 wild type, based on data from Stroud et al.^7^. Methylation levels are shown for gbM mCG (**e**), TE mCG (**f**), global mCHG (**g**), and global mCHH (**h**). **i-l**, DNA methylation levels in newly generated *vim2* and *vim4* single mutants compared to Col-0 wild type, for gbM mCG (**i**), TE mCG (**j**), global mCHG (**k**), and global mCHH (**l**). Dots represent biological replicates (n = 3 per genotype) and bars indicate mean values. P values were calculated using two-sided t-tests relative to Col-0 wild type; error bars represent standard errors. Source data

Consistent with the RIL mapping results, overexpression of *VIM2*, *VIM4* or *VIM24* caused global CG hypomethylation, reducing global mCG levels from 29.1% in wild type to as low as 8.0% in the strongest hypomethylated overexpression line (~72% relative reduction), irrespective of whether the transgenes carried the Col-0 or Cvi-0 alleles (Fig. 5a and Extended Data Fig. 6a,b). CHG and CHH methylation were comparatively less affected (Extended Data Fig. 6c,d). Consistent with our QTL mapping (Figs. 2c and 3c), elevated VIM2/4 dosage preferentially disrupted gbM regions compared with TEs (causing up to an 84% versus 54% relative reduction, respectively), confirming the preferential sensitivity of gbM loci to *VIM2*/*4* perturbation (Extended Data Fig. 6a,b). These results show that increased *VIM2*/*4* expression, rather than allelic variation per se, is the primary cause of the observed methylation defects. They also indicate that *VIM2* and *VIM4* are functionally similar in their ability to perturb mCG maintenance and that the Cvi-0 *VIM4* allele remains active despite the premature stop codon described above. This is in line with our structural prediction that the truncated Cvi VIM4 retains functionality (Extended Data Fig. 4a). Notably, however, global mCG levels did not decrease monotonically with *VIM2*/*4* expression. Moderate overexpression reproducibly caused CG hypomethylation, whereas a subset of lines with extremely high *VIM2*/*4* expression (>1,000-fold increased relative to wild type) retained near-wild-type or slightly elevated mCG levels (Fig. 5a,b). Thus, *VIM2*/*4* expression levels appear to affect mCG in a strongly nonlinear manner, consistent with a dosage window in which increased *VIM2*/*4* expression is the most disruptive to methylation maintenance (Fig. 5b and Supplementary Fig. 4).

**Extended Data Fig. 7:**
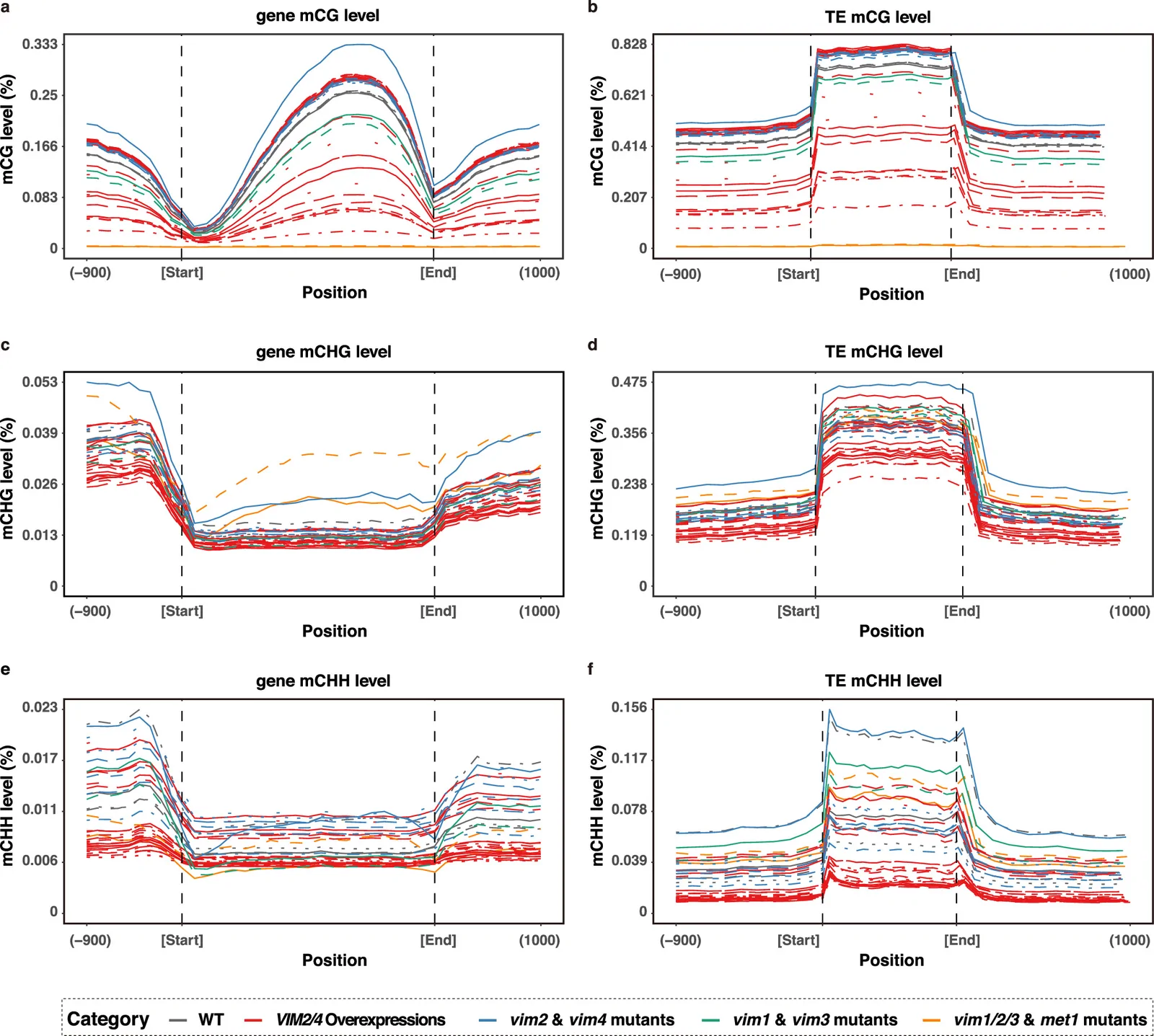
Metaplot profiles of DNA methylation across genes and TEs show context-specific effects of VIM2/4 dosage perturbation. **a–f**, Metaplots of DNA methylation levels across genes and transposable elements (TEs) in wild type (WT), *VIM2/4* overexpression lines, *vim2* and *vim4* single mutants, *vim1* and *vim3* single mutants, and *vim1/2/3* and *met1* mutants. Profiles are shown for gene mCG (**a**), TE mCG (**b**), gene mCHG (**c**), TE mCHG (**d**), gene mCHH (**e**) and TE mCHH (**f**). Feature bodies are scaled from start to end positions, with 900 bp upstream and 1,000 bp downstream flanking regions shown. Consistent with global methylation analyses, many *VIM2/4* overexpression lines show pronounced CG hypomethylation across genes and TEs, with the strongest relative reduction across gene bodies, whereas *vim2* and *vim4* mutants show reciprocal CG hypermethylation. Non-CG methylation profiles are comparatively less affected. Line colours indicate genotype categories, and line types within each colour distinguish individual samples.

If elevated *VIM2*/*4* expression reduces mCG, then disrupted *VIM2*/*4* activity should be expected to exert the opposite effect. To test this prediction, we re-analysed published WGBS data from *vim1*, *vim2* and *vim3* single mutants and the *vim1/2/3* triple mutant^7^. We found that *vim2* exhibits pronounced global CG hypermethylation, whereas *vim1* and *vim3* show only mild hypomethylation (Fig. 5c and Extended Data Fig. 6e–h). To validate this inverse dosage effect experimentally, we grew independent *vim2* and *vim4* T-DNA insertion mutants and generated WGBS data from multiple biological replicates (Supplementary Table 20 and Methods). These new data confirmed that mutations in either *VIM2* or *VIM4* cause statistically significant global CG hypermethylation (Fig. 5d and Extended Data Fig. 6i,j), in sharp contrast to the hypomethylation associated with *vim1* and *vim3* (Fig. 5c and Extended Data Fig. 6e,f). Non-mCG levels were largely unaffected in *vim2* or *vim4* (Extended Data Fig. 6k,l), mirroring the overexpression lines and indicating that *VIM2*/*4* expression primarily affects mCG maintenance (Fig. 5a–e and Extended Data Figs. 6 and 7).

Together with the overexpression results, these findings establish a novel role of *VIM2*/*4* as dosage-dependent negative regulators of mCG maintenance (Fig. 5a–e and Extended Data Figs. 6 and 7). These findings challenge the prevailing view that *VIM1* (AT1G57820), *VIM2* and *VIM3* (AT5G39550) are largely redundant^7,9,57–59^. Instead, there may be a functional division within the *Arabidopsis* VIM family, with *VIM2*/*4* and *VIM1*/*3* (*VIM1* and *VIM3*) forming two distinct subgroups that exert opposing effects on mCG.

### Dysregulation of *VIM2*/*4* perturbs DNA methylation pathways

**Extended Data Fig. 8:**
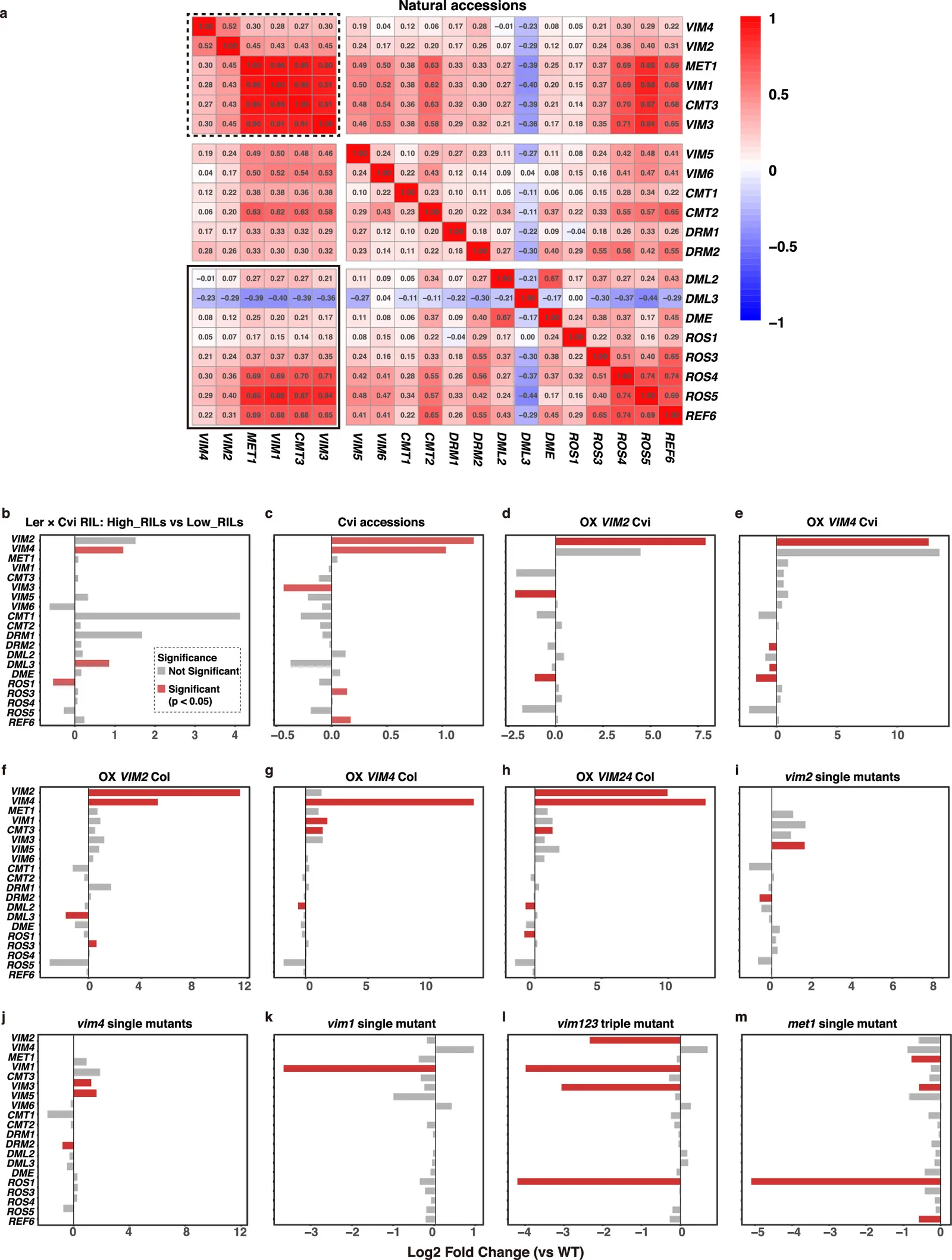
*VIM2/4* are embedded in the DNA methylation transcriptional network and their dysregulation perturbs methylation regulator expression across multiple genetic backgrounds. **a**, Gene co-expression correlation network for DNA methylation pathway genes across natural *A. thaliana* accessions. *VIM2* and *VIM4* show strong co-expression with each other and with core components of the CG methylation machinery, including *MET1*, *VIM1*, and *VIM3*, as well as the CHG methyltransferase *CMT3*, forming a co-expression module highlighted by the dashed box, illustrating integrated methylation maintenance pathways. The solid box indicates co-expression patterns between methylation maintenance genes and DNA demethylases. Colour scale indicates Pearson’s correlation coefficients ranging from -1 (blue) to 1 (red). **b-m**, Expression changes of 20 DNA methylation regulators across 12 comparisons in multiple independent experimental systems. Bars show log2 fold changes for high- versus low-epimutation-rate Ler × Cvi RILs (**b**), Cvi accessions stratified by presence versus absence of the Cvi-specific *VIM2/4* deletion (**c**), transgenic overexpression lines (OX) with the Cvi allele of *VIM2* (OX VIM2 Cvi) (**d**), *VIM4* (OX *VIM4* Cvi) (**e**), and the Col-0 allele of *VIM2* (OX *VIM2* Col) (**f**), *VIM4* (OX *VIM4* Col) (**g**), and both *VIM2* and *VIM4* simultaneously (OX *VIM24* Col) (**h**), *vim2* single mutants (**i**), *vim4* single mutants (**j**), *vim1* single mutant (**k**), *vim1/2/3* triple mutant (**l**) and *met1* single mutant (**m**). For **b** and **c**, positive values indicate higher expression in high-epimutation-rate RILs and deletion-carrying Cvi accessions, respectively. For **d–m**, positive values indicate higher expression relative to their respective wild-type controls. Significant differences (P < 0.05, two-sided Wald tests via DESeq2) are highlighted in red.

To assess whether VIM2/4 dosage affects the broader epigenetic regulatory network beyond CG maintenance, we examined transcriptional relationships among core methylation-pathway components. In both the MA-ML population and natural accessions (1001 transcriptome data^3^), *VIM2* and *VIM4* were strongly co-expressed with each other and with central components of the DNA methylation machinery, including *MET1*, *VIM1*, *VIM3*, *CMT3* and the DNA demethylase ROS1 (AT2G36490) (Fig. 5f, Extended Data Fig. 8a and Supplementary Table 14). This transcriptional association places *VIM2*/*4* within the central DNA methylation network, consistent with protein functional association analysis from the STRING database^60^ (Supplementary Fig. 5). This network relationship was disrupted in MA-ML lines carrying the Cvi-derived *VIM2*/*4* haplotype. Despite elevated *VIM2*/*4* expression, these lines exhibited reduced transcriptional connectivity with other methylation-related genes and *ROS1* downregulation (Fig. 5f,g). The downregulation of these components was also evident in independent transcriptome comparisons between Cvi-0 and Col-0^56^ (Fig. 5h), and *ROS1* downregulation was further confirmed in new RNA-seq data from RILs with contrasting epimutation rates (Extended Data Fig. 8b). Thus, the Cvi *VIM2*/*4* haplotype is associated not only with altered mCG maintenance but also with transcriptional rewiring of the broader methylation network (Fig. 5f–h and Extended Data Fig. 8a–c).

To test whether this rewiring is driven by VIM2/4 dosage itself, we generated RNA-seq data from *VIM2*/*4* overexpression and loss-of-function lines (Supplementary Table 21). The hypomethylated *VIM2*/*4* overexpression lines recapitulated key features of the Cvi deletion background, including frequent downregulation of *VIM1*, *VIM3* and *ROS1* (Fig. 5a,g–n, Extended Data Fig. 8d–h, and Supplementary Figs. 6 and 7). The resulting hypomethylation could not be explained by transgene-induced post-transcriptional gene silencing (PTGS) of *VIM1/3* because some overexpression lines maintained or even increased *VIM1/3* expression while still showing mCG maintenance defects and there was no PTGS evidence for increased CG and non-mCG in these genes (Fig. 5a,k,l, Extended Data Fig. 8d,e,h, and Supplementary Figs. 7 and 8). Conversely, *vim2*/*4* mutants showed reciprocal upregulation of these same components (Fig. 5k–n, Extended Data Fig. 8i,j and Supplementary Figs. 6–8). Analogous feedback was not observed in *vim1* (Fig. 5i–n and Extended Data Fig. 8k–m), indicating that *VIM2*/*4* have a distinct regulatory role within the maintenance network. Together, these results show that altered VIM2/4 dosage is sufficient to perturb the stoichiometric balance of DNA methylation components and to trigger secondary transcriptional changes in interacting pathways.

**Extended Data Fig. 9:**
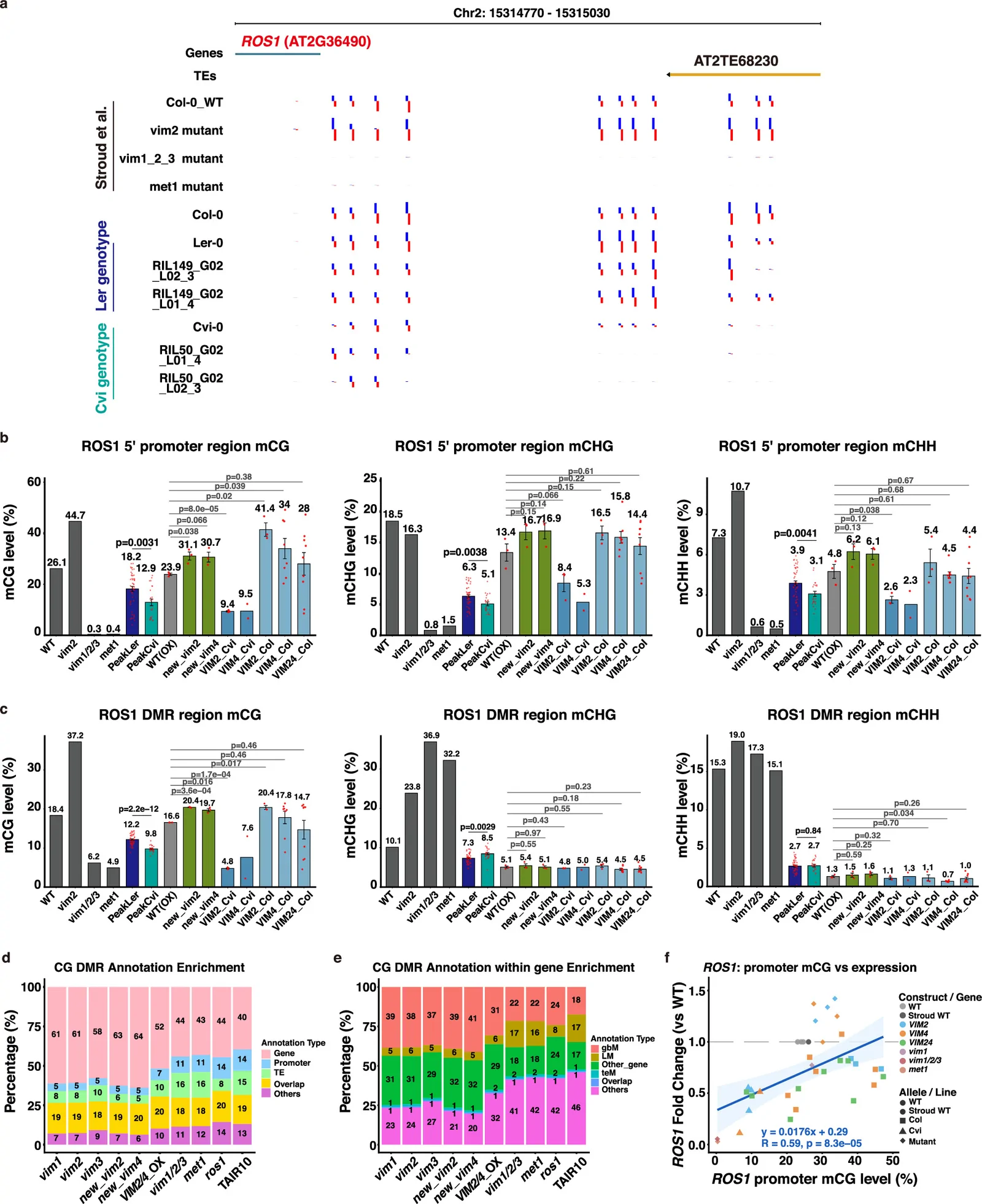
*ROS1* promoter methylation dynamics explain its expression changes under *VIM2/4* perturbation, and VIM2/4-associated DMRs are genomically distinct from ROS1 target loci. **a**, Genome browser tracks displaying DNA methylation states across the *ROS1* promoter and its adjacent Helitron element and DNA methylation monitoring sequence (MEMS). **b,c**, Methylation levels at the *ROS1* 5′ promoter (**b**) and at canonical ROS1 target DMRs defined in *ros1* mutants (**c**), for CG (left), CHG (middle) and CHH (right), across published *vim/met1* mutants, RILs stratified by chromosome 1 QTL genotype (PeakLer, PeakCvi), newly generated *vim2* and *vim4* single mutants (new_*vim2* and new_*vim4*), and *VIM2/4* overexpression lines carrying Cvi or Col alleles. Bars, mean; error bars, standard error of the mean (SEM). Dots, individual biologically independent samples: one RIL line (RIL-level mean) for PeakLer and PeakCvi, one WGBS sample from one plant otherwise. n = 46, 22, 3, 3, 3, 3, 2, 3, 8 and 9 for PeakLer, PeakCvi, WT(OX), new_*vim2*, new_*vim4*, VIM2_Cvi, VIM4_Cvi, VIM2_Col, VIM4_Col and VIM24_Col, respectively. VIM4_Cvi (n = 2) is shown descriptively and excluded from inferential statistical testing. *P* values, two-sided t-tests versus PeakLer (RILs) or versus Col-0 wild type (all other groups). **d-e**, Genomic annotation enrichment of CG DMRs across vim mutants, *VIM24* overexpression (*VIM2/4*_OX), *vim1/2/3*, *met1*, and *ros1* mutant, compared to the TAIR10 genomic background. Panel **d** shows enrichment across broad annotation categories. Panel **e** shows gene-associated annotations further separated into gene body methylated (gbM), lowly methylated (LM), TE-like methylated (teM), and other gene-associated regions. Overlap denotes regions assigned to two or more of the listed annotation categories, and “Others” denotes genomic regions not assigned to any of the above categories. **f**, Scatter plot demonstrating a positive correlation between *ROS1* promoter mCG levels and *ROS1* gene expression. Solid blue lines show the linear regression fit (predicted mean), and shaded areas represent the 95% confidence interval. Statistics are Pearson’s correlation coefficient (R) and the corresponding two-sided *P* value. Source data

Among the affected genes, *ROS1* provided the clearest mechanistic link between methylation perturbation and transcriptional response. *ROS1* was consistently downregulated in globally hypomethylated backgrounds, including high-epimutation-rate RILs, Cvi-0, *VIM2*/*4* overexpression lines, *vim1/2/3* and *met1*, whereas it was upregulated in hypermethylated *vim2*/*4* mutants^10,57^ (Fig. 5n). This bidirectional response is consistent with the known methylation-sensitive *ROS1* promoter rheostat, formed by the adjacent Helitron element and DNA methylation monitoring sequence (MEMS)^61,62^. Concordantly, reduced *ROS1* expression in globally hypomethylated backgrounds was accompanied by hypomethylation across the MEMS–Helitron sequence, while *vim2*/*4* showed the reciprocal methylation state at the same region (Extended Data Fig. 9a,b).

As *ROS1* encodes a DNA demethylase, its downregulation should increase methylation at ROS1-sensitive loci, whereas RILs carrying the Cvi deletion haplotype show predominant genome-wide CG hypomethylation, most pronounced across gbM genes. To resolve this apparent contradiction, we examined canonical ROS1 target regions defined by differentially methylated region (DMR) analysis of *ros1* mutants relative to wild type^63^ (Extended Data Fig. 9c and Methods). As expected under reduced ROS1 activity, these regions showed increased relative CHG methylation in RILs carrying the chromosome 1 Cvi haplotype. However, relative mCG at the same loci was lower in Cvi-haplotype than in Ler-haplotype RILs (Extended Data Fig. 9c). Moreover, DMR annotation analysis showed that *VIM2*/*4* overexpression and mutant DMRs are predominantly enriched in gbM regions, whereas ROS1-target DMRs represent a distinct class of loci that lie largely outside these gbM regions (Extended Data Fig. 9d,e). Thus, *ROS1* repression is a detectable downstream consequence of *VIM2*/*4* dysregulation, with methylation effects at specific ROS1-sensitive regions (Extended Data Fig. 9f) but it does not explain nor contradict the genome-wide CG maintenance defect.

Together, these findings identify the Cvi *VIM2*/*4* haplotype as a naturally segregating variant that perturbs DNA methylation-network balance, providing a framework for understanding how genetic modifiers can reshape both the steady state and the long-term dynamics of epigenome inheritance.

## Discussion

Genetic mapping studies of DNA methylation usually treat steady-state methylation differences as static end points. Here, we show that natural genetic variants can also modify the fidelity with which methylation states are transmitted across generations, thereby altering how DNA methylomes evolve over time in a fixed genetic background. Earlier efforts to identify genetic modifiers of spontaneous methylation variation relied on somatic deviations from expected methylation patterns in an F2 population^64^ and could therefore not observe transgenerational epimutation dynamics directly. By contrast, the MA-ML experimental design allowed us to link genetically induced perturbations in global mCG levels to increased CG epimutation rates and accelerated epigenetic drift over multiple generations. This coupling supports a punctuated-equilibrium-like view of mCG evolution, in which genetic perturbations of the maintenance machinery can produce abrupt shifts in methylation equilibrium while also changing the rate at which methylation states fluctuate over time. It further suggests that CG maintenance fidelity may decline when the methylation system is displaced from its normal homeostatic range, as also observed in DNA methylation-pathway mutants^18^.

**Extended Data Fig. 10:**
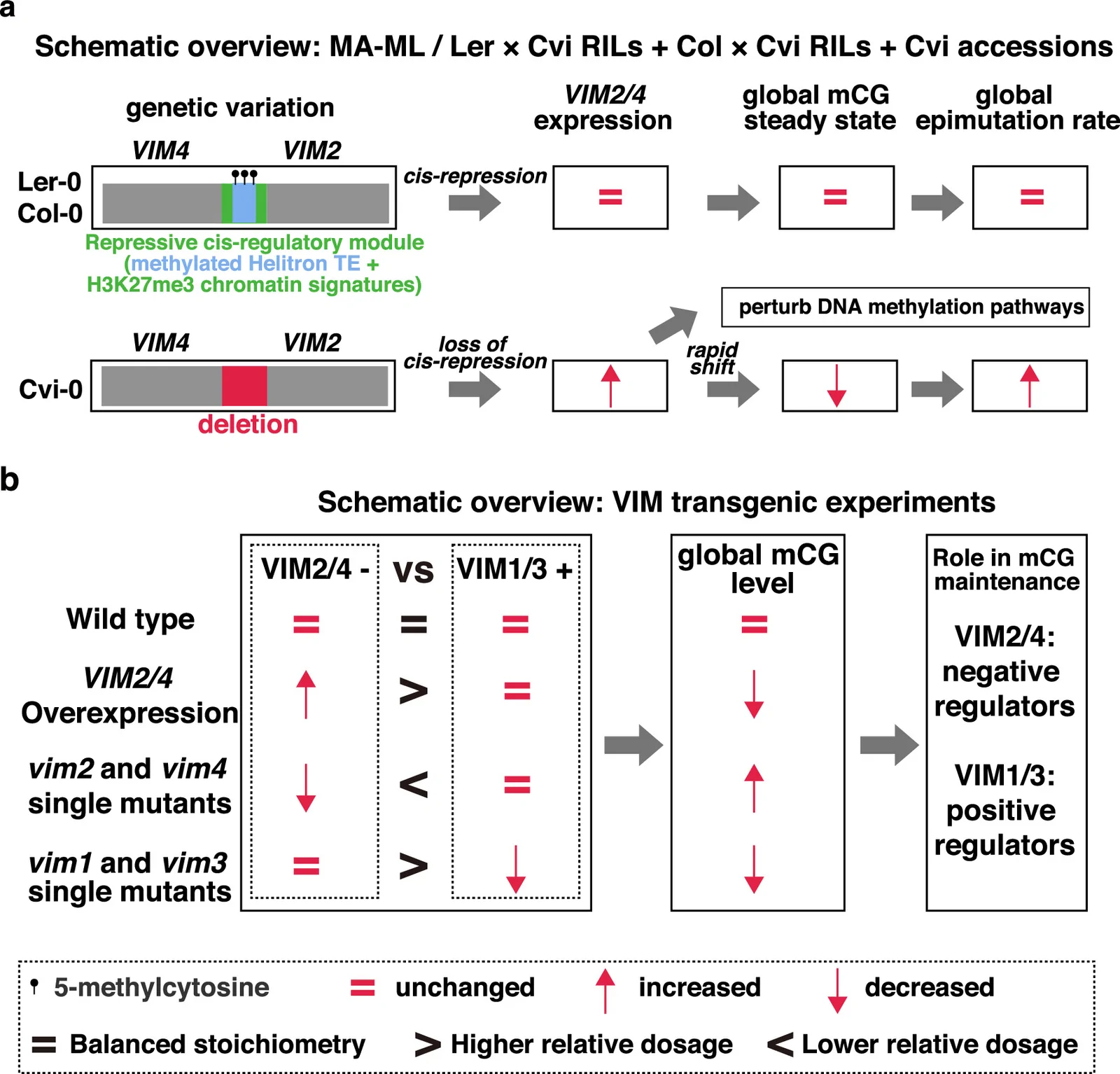
Schematic summary of the genetic and dosage effects of the VIM2/4 on DNA methylation maintenance. **a**, Schematic overview of the genetic mechanism identified through MA-ML, Ler × Cvi RILs, Col × Cvi RILs, and natural Cvi accessions. In the Ler-0 and Col-0, a repressive cis-regulatory module comprising a methylated Helitron transposable element and H3K27me3 chromatin signatures maintains basal *VIM2/4* expression, ensuring faithful CG methylation maintenance (stable steady state and basal epimutation rate). The Cvi-0-derived deletion removes this repressive module, causing a loss of cis-repression and elevating *VIM2/4* expression. This *VIM2/4* upregulation drives a rapid shift toward a lower global mCG steady state, increases global epimutation rate, and perturbs DNA methylation pathways. **b**, Summary diagram of VIM transgenic and mutant experiments establishing the functional division within the VIM family. Under wild-type conditions, balanced stoichiometry between *VIM2/4* and *VIM1/3* maintains normal global CG methylation levels. *VIM2/4* overexpression shifts the balance toward a higher relative *VIM2/4* dosage, leading to reduced global mCG levels. Conversely, *vim2* and *vim4* single mutants lower VIM2/4 dosage and cause reciprocal CG hypermethylation. These reciprocal effects establish VIM2/4 as dosage-sensitive negative regulators of mCG maintenance. In contrast, loss of VIM1 and VIM3 (*vim1* and *vim3* single mutants) reduces global mCG levels, confirming their canonical roles as positive regulators. Arrows and equal signs indicate the direction of change relative to the wild type. The symbols =, >, and < indicate a balanced, higher, or lower relative stoichiometric dosage of VIM2/4 compared to VIM1/3, respectively.

Our mapping identified a Cvi-derived deletion at a major meQTL locus flanking *VIM2* and *VIM4*, which was associated with elevated expression of both genes (Extended Data Fig. 10a). Overexpression and loss-of-function experiments confirmed that this dosage change is functionally relevant. Increased VIM2/4 dosage lowered the mCG steady state, whereas reduced *VIM2*/*4* activity caused reciprocal CG hypermethylation (Extended Data Fig. 10b). A recent study by Shin et al.^65^ attributed Cvi-associated CG hypomethylation to an RNAi-based mechanism initiated by the Cvi-specific *VIM4*–*VIM2* structural configuration. In their model, the shortened intergenic region between the oppositely oriented *VIM4* and *VIM2* genes permits extended or read-through transcription across highly homologous sequences, generating stem-loop or double-stranded RNA substrates that are processed into *VIM*-derived siRNAs. On the basis of these data in seed tissue, they proposed that these siRNAs silence *VIM1* in trans, thereby reducing *VIM1/3*–*MET1*-dependent CG maintenance and causing genome-wide hypomethylation.

Several observations argue that this RNAi-based model is not sufficient to explain the methylation phenotype observed here. First, *VIM2*/*4* overexpression was sufficient to induce hypomethylation in both Cvi and Col-0 backgrounds (Figs. 4 and 5 and Extended Data Figs. 5–8), indicating that the Cvi-specific inverted-repeat configuration is not required for the phenotype. Second, across multiple datasets carrying the Cvi deletion, we did not observe consistent downregulation of *VIM1* or *VIM3* in leaf, nor evidence of PTGS at the methylation level of these genes, arguing against reduced *VIM1*/*3* expression as the primary cause of the methylation defect in this tissue (Fig. 5, Extended Data Fig. 8, and Supplementary Figs. 7 and 8). Although leaf methylation is not itself the substrate of transgenerational inheritance, leaf *VIM2*/*4* expression levels predicted both the inherited hypomethylated state and the associated epimutation instability across generations, indicating that the leaf phenotype reflects a dosage state relevant to inherited methylome dynamics rather than an isolated terminal-tissue effect. Finally, the non-monotonic dosage response observed in some overexpression lines, where extremely high *VIM2*/*4* expression retains near-wild-type mCG levels, is difficult to reconcile with a simple RNAi-mediated loss-of-function model (Fig. 5a,b and Supplementary Fig. 4).

**Fig. 6:**
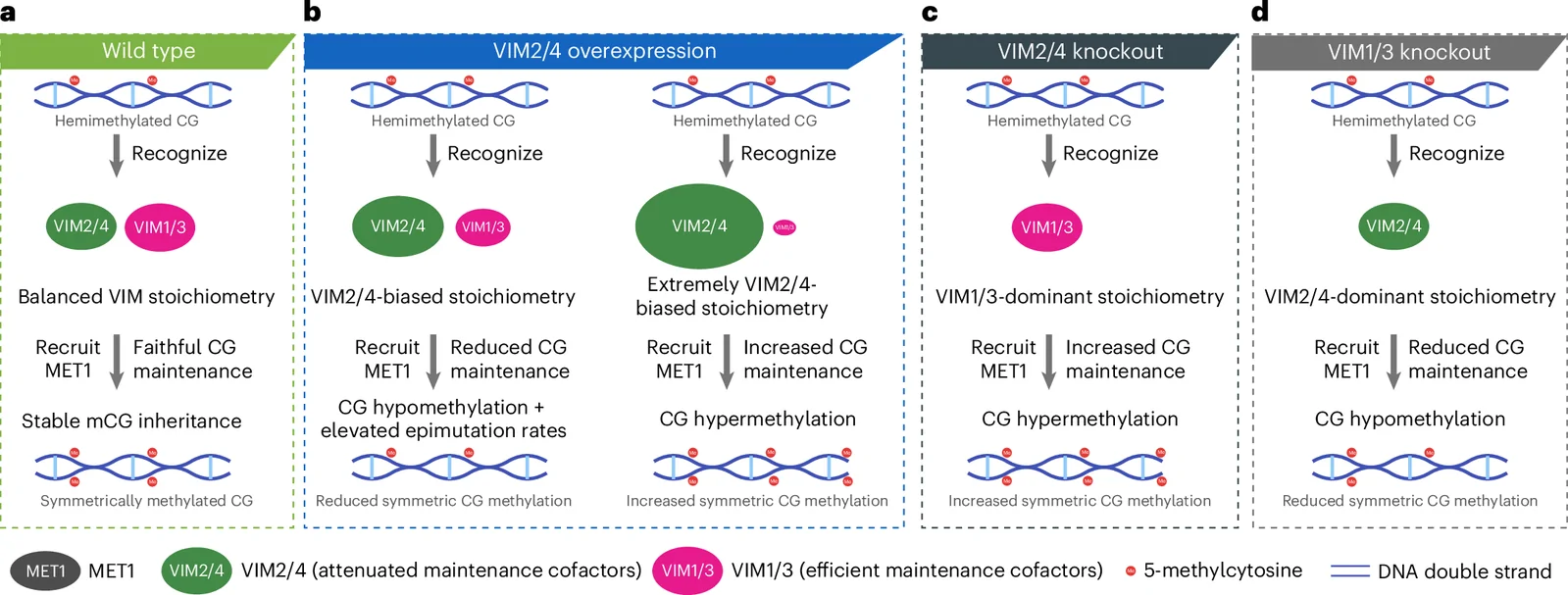
Working genetic model: dosage-sensitive competition among VIM paralogues modulates MET1-dependent CG maintenance. Schematic illustrating how the stoichiometric balance between VIM protein subgroups is proposed to modulate the efficiency of MET1-dependent mCG copying at hemimethylated CG sites after DNA replication. VIM2/4 (green) are modelled as attenuated cofactors of MET1-dependent CG maintenance that compete with VIM1/3 (pink), the more efficient cofactors, for shared components of the maintenance machinery, whether hemimethylated CG substrates, MET1 binding or both. **a**, In wild-type plants, a balanced stoichiometry between VIM1/3 and VIM2/4 ensures efficient MET1 recruitment to hemimethylated DNA, resulting in faithful mCG maintenance and stable epigenetic inheritance. **b**, Elevated VIM2/4 dosage shifts this balance. Moderate overexpression creates a VIM2/4-biased stoichiometry that, through competition, leads to reduced CG maintenance, elevated epimutation rates and global CG hypomethylation (left). Conversely, extremely VIM2/4-biased stoichiometry (>1,000-fold relative to wild type) can overcome this inefficiency through a ‘mass-action’ effect when the system is flooded with VIM2/4, driving retention of near-wild-type or even slightly hypermethylated CG levels (right), consistent with the nonlinear dosage relationship observed in overexpression lines (Fig. 5b). **c**, In *vim2*/*4* mutants, the relief of competitive binding results in a VIM1/3-dominant stoichiometry, which causes CG hypermethylation (Fig. 5c,d). **d**, In *vim1*/*3* mutants, the attenuated VIM2/4 proteins dominate the maintenance complex, causing CG hypomethylation, analogous to moderate *VIM2*/*4* overexpression (Fig. 5a,c). This schematic represents a genetic dosage model. The biochemical properties of the VIM proteins, including their associations with one another, with MET1, and with chromatin, probably contribute to MET1 recruitment and the observed mCG phenotypes through mechanisms that remain to be explored.

Together, our observations identify *VIM2*/*4* as dosage-sensitive negative regulators of mCG maintenance, functionally distinct from the canonical positive roles of *VIM1*/*3* (Extended Data Fig. 10b). These findings challenge the prevailing view that *VIM1*, *VIM2* and *VIM3* are largely redundant^7,9,57–59^. Although the underlying biochemical mechanism remains unresolved, one possible explanation is that VIM2/4 compete with VIM1/3 for hemimethylated CG templates or MET1 binding but differ in their ability to recruit, stabilize or functionally couple with MET1-dependent methylation copying (Fig. 6). In this genetic dosage model, moderate increases in VIM2/4 dosage would shift maintenance-complex stoichiometry away from a more faithful VIM1/3-dominated configuration, thereby lowering mCG. Conversely, reduced VIM2/4 activity would relieve this competition and increase maintenance efficiency. The observation that some lines with extremely high *VIM2*/*4* expression (>1,000-fold higher than wild type) retained near-wild-type mCG levels is also consistent with this framework because the absolute number of VIM2/4-bound hemimethylated templates or MET1-recruitment events may become sufficient to compensate for lower per-complex efficiency (Fig. 5a,b). This model is also consistent with the previous observation that the *vim1*/*2*/*3* triple mutant exhibits more severe global hypomethylation than the *vim1*/*3* double mutant^9^. Future work should determine whether VIM2/4 directly compete with VIM1/3 for hemimethylated templates, differ in their interaction with MET1, associate with one another through intrafamily dimerization or with chromatin, undergo concentration-dependent condensate formation or alter the stability and turnover of maintenance complexes.

This dosage-sensitive logic may extend beyond plants. Mammalian UHRF1, the homologue of plant VIM proteins^9,66–68^ (Supplementary Fig. 9 and Supplementary Table 22), recruits DNMT1, the mammalian homologue of MET1, to hemimethylated CG sites during DNA methylation maintenance^1,11–16^. *UHRF1* is frequently overexpressed in human cancers^69–71^, where elevated expression has been linked to disruption of *DNMT1*-dependent maintenance, widespread DNA hypomethylation and tumorigenesis^72–74^. Although plant VIM proteins and mammalian UHRF1 are not mechanistically equivalent, these parallels suggest that the dosage of maintenance-associated recognition factors must be tightly regulated to preserve epigenomic stability.

Our study also shows that *VIM2*/*4* perturbation is not confined to CG copying itself but propagates into the broader methylation network, most notably to the DNA demethylase ROS1 (Fig. 5f–n, Extended Data Figs. 8 and 9, and Supplementary Figs. 6 and 7). The association between the Cvi deletion, elevated *VIM2*/*4* expression and reduced *ROS1* expression points to substantial crosstalk between maintenance and demethylation pathways. However, *ROS1* repression neither explains nor contradicts the predominant methylation phenotype of the Cvi haplotype. Reduced ROS1 activity is expected to promote methylation gain at ROS1-sensitive loci, whereas the major QTL effect is genome-wide CG hypomethylation, the strongest across gbM genes. Consistently, canonical ROS1 target regions showed signatures of reduced demethylase activity but represented a distinct class of loci from those driving broad gbM-associated hypomethylation (Extended Data Fig. 9). *ROS1* is therefore best viewed as a downstream component of *VIM2*/*4* perturbation rather than as the primary cause of the CG maintenance defect.

Whether environmental perturbations engage the same *VIM2*/*4*–*ROS1* axis remains an important question. Biotic and abiotic stress can affect DNA methylation pathways but it is unclear whether such responses intersect with *VIM2*/*4*-dependent CG maintenance, *ROS1*-mediated demethylation or both^63^. Our recent observations that loss of ROS1 modifies the effect of multigenerational biotic stress on DNA methylation maintenance fidelity are consistent with such crosstalk. However, these effects occur mainly outside gbM genes, whereas *VIM2*/*4*-dependent epimutation-rate changes primarily target gbM-associated mCG. This distinction suggests that genetic and environmental perturbations may converge on shared methylation-network components while affecting different genomic compartments.

Although the Cvi-derived *VIM2*/*4* haplotype accelerates mCG drift, this increased epigenetic divergence did not translate into detectable divergence in chromatin accessibility or gene expression over the timescale examined here (Extended Data Fig. 3). This is consistent with the view that many spontaneous CG epimutations, particularly in gbM regions, behave largely neutrally or have weak immediate regulatory effects^24^. At the same time, this global neutrality does not imply that all methylation changes are functionally buffered. The ROS1 locus illustrates how specific regulatory nodes can remain highly sensitive to methylation perturbation through local feedback mechanisms, including the MEMS–Helitron rheostat. Thus, cumulative CG epimutational drift may be largely neutral at the genome-wide level while still engaging local epigenetic feedback loops at responsive loci. Over longer timescales, increased epimutational turnover may therefore influence the standing epigenetic variation on which drift, selection or genetic interactions can act^75^, although evidence for this remains controversial^76^.

Together, our findings establish spontaneous CG epimutation rate as a heritable quantitative trait and reposition *VIM2*/*4* from presumed redundant members of the *VIM* family to dosage-sensitive negative modulators of both the equilibrium and fidelity of mCG maintenance. This genetic–epigenetic interplay provides a mechanistic basis for a punctuated-equilibrium-like model of methylome evolution, in which naturally segregating modifiers of mCG homeostasis can produce abrupt shifts in methylation state and alter the rate at which heritable epigenetic variation accumulates in plant genomes.

## Methods

### Construction and omics profiling of the MA-ML

To be able to identify specific genetic loci contributing to intraspecific variation in epimutation rates, we generated selfing-derived MA-ML in the model plant *A. thaliana* (Fig. 1f). The MA-ML comprise 68 genetically distinct MA pedigrees (with eight lineages each), which are derived from 68 different founders RILs. These RILs were initially obtained from a cross between Landsberg erecta (Ler-0) and Cape Verde Islands (Cvi-0) accessions.

The MA-ML were grown in climate-controlled chambers for ten selfing generations (long-day conditions, 16-h day lengths, 18 °C). Seeds from each generation were stored. To obtain plant material for WGBS, we grew plants from G2 and G9 seeds in a common growth chamber, following the sampling scheme shown in Fig. 1f. Samples were collected from young above-ground rosette leaf tissue. Leaf tissue was flash frozen in liquid nitrogen, and DNA was extracted using a Qiagen Plant DNeasy kit (Qiagen) on the basis of the manufacturer’s instructions. MethylC-seq libraries were prepared on the basis of the protocol described by Urich et al.^77^. Libraries were sequenced at Genewiz on a NovaSeq 6000 platform (Illumina) in a paired-end 150-bp format.

ATAC-seq was performed across generations in selected pedigrees with contrasting epimutation rates. ATAC-seq samples were collected from the immediate progeny of the sibling plants used for WGBS (Extended Data Fig. 3a). The lines were grown under long-day conditions (16 h light/8 h dark) on 1/2 MS plates at 25 °C for 7–9 days. For the nuclei extraction, approximately 200 mg bulk seedlings were collected. The samples were chopped down in 1 ml prechilled lysis buffer (10 mM MES-KOH pH 5.4, 10 mM NaOH, 250 mM sucrose, 0.1 mM spermine, 0.5 mM spermidine, 1 mM dithiothreitol (DTT), 1% BSA, 0.5% TritonX-100) and filtered through a 40-µm and a 20-µm strainer, with a centrifugation step in between (500*g*, 5 min). The nuclei were finally pelleted with a gradient solution (35% Percoll in lysis buffer) in a swing centrifuge at 500*g* for 10 min and resuspended in tagmentation buffer (10 mM TAPS-NaOH pH 8.0, 5 mM MgCl_2_). Nuclei quality and concentration were checked with 4,6-diamidino-2-phenylindole (DAPI) staining at a cell counter. 50k–100k purified nuclei were incubated with 3 µl Tn5 enzyme in 40 µl tagmentation buffer (described above) at 37 °C for 30 min. The products were purified using a PCR-extraction kit (NEB Monarch PCR & DNA Clean-up, #T1030) and then amplified for 10–12 cycles using Q5 polymerase (NEBNext HiFi 2x PCR Mastermix, #M0541). PCR conditions were followed as described previously^78^. Amplified libraries were purified with AMPure beads, and concentrations were determined using Qubit and qPCR (Roche KAPA Library Quantification Kit) before sequencing. Paired-end 150-bp sequencing was performed on an Illumina NovaSeq 6000 platform.

RNA-seq was performed on siblings of the plants used for ATAC-seq following the same growth conditions. Total RNA was extracted from 7- to 9-day-old bulk seedlings following the TRIzol extraction method. Libraries were constructed using the TruSeq Stranded mRNA Library Kit (Number 20020594, Illumina) with a custom dual index workflow of iTruSeq primer set and iTruSeq adapter^79^. Paired-end 150-bp sequencing was performed on an Illumina NovaSeq 6000 platform.

### Construction and omics profiling of *VIM2*/*4* overexpression lines

For *VIM2*/*4* overexpression lines in the Col-0 background, five plasmids were constructed (*VIM2* and *VIM4* carrying either the Col-0 or Cvi-0 allele and a combined *VIM2* and *VIM4* construct carrying the Col-0 allele) using Golden Gate cloning^80^. The coding sequences of VIM genes were synthesized by GeneArt (Thermo Fisher), using the TAIR10 sequence for Col-0 and the proposed sequence from the long-assembly data for Cvi-0 (see the ‘Published datasets’ section). To induce gene overexpression, the CaMV 35S promoter and NOS terminator were used but no tags were added. Final constructs were confirmed by ONT-Whole Plasmid sequencing (Eurofins Genomics) before being transformed into *A. thaliana* Col-0 using the floral dip method and the *Agrobacterium* strain GV3101 (pSoup-P19)(CC-125-5×50, Goldbio). Detailed cloning strategy and construct information is presented in Supplementary Table 23. Transgenic lines were selected in the presence of 50 µg ml^−1^ kanamycin. T1 plants were tested for overexpression by qPCR and used for further analyses. The qPCR followed the same protocol used for the MA-ML, with a different primer pair specific to *VIM2*, *VIM3* and *VIM4* in Col and Cvi, yielding a single 206-bp amplicon with an efficiency of 92%. Detailed primer information is presented in Supplementary Table 24.

For WGBS, genomic DNA was extracted from mature rosette leaves of qPCR-confirmed T1 overexpression plants once sufficient leaf tissue was available. For each library, 200 ng genomic DNA was sheared into 350-bp fragments using a Covaris R230 ultrasonicator. Libraries were generated following the protocol of NEBNext Ultra II DNA Library Prep kit (E765S, NEB) in combination with NEBNext EM-seq Adaptor (E7165AVIAL, NEB) and NEBNext Multiplex Oligos for Enzymatic Methyl-seq (E7140L, NEB). Bisulfite conversion was performed with the EZ-96 Methylation-Gold MagPrep kit (D5042, Zymo Research) before the PCR enrichment step, where the NEB Q5 polymerase was substituted by KAPA HiFi HotStart Uracil+ReadyMix (KK2801, Roche) to enable the amplification of uracil-containing DNA. Size selection and cleanup were performed with magnetic beads (AMPure XPbeads, A63881, Beckman Colter), and the quality of the final libraries was assessed with the Qubit dsDNA HS Assay Kit (Q32851, Invitrogen). Sequencing was performed by Novogene using an Illumina NovaSeq X Plus (PE150) platform. Furthermore, SNPs extracted from the WGBS data were utilized to verify the uniform Col-0 genetic background of all *VIM2*/*4* overexpression lines, as well as the correct identity of the introduced Col or Cvi allele of the *VIM2*/*4* transgene in each line.

For RNA-seq, total RNA was extracted from mature rosette leaves of qPCR-confirmed T1 overexpression plants once sufficient leaf tissue was available, using the Monarch Total RNA Miniprep Kit (N2010, NEB). RNA concentration was quantified using the Qubit RNA BR Assay Kit (Q33223, Thermo Fisher), and samples were sent to Novogene for library preparation and paired-end 150-bp sequencing on an Illumina NovaSeq X Plus platform.

### T-DNA insertion *vim2* and *vim4* single-mutant lines

Homozygous T-DNA insertion mutant lines for *vim2* (stock number SALK_141723C; NASC ID: N664186; T-DNA insertion in *VIM2*, AT1G66050) and *vim4* (stock number SALK_073322C; NASC ID: N675753; T-DNA insertion in *VIM4*, AT1G66040) were obtained from the Nottingham *Arabidopsis* Stock Center (NASC; https://arabidopsis.info/). Both lines are in the Col-0 background and were confirmed as homozygous by the SALK Institute before distribution. The T-DNA insertion lines were independently verified by PCR-based genotyping using OneTaq DNA Polymerase (#M0480, NEB) as previously described^81^ and were confirmed by Sanger sequencing. Detailed primer information is presented in Supplementary Table 24. Three biological replicates for each line were used for WGBS and RNA-seq, following the same procedures as for *VIM2*/*4* overexpression lines.

### Published datasets

This study integrates newly generated data with previously published datasets spanning WGBS, RNA-seq, ATAC-seq, chromatin immunoprecipitation followed by sequencing (ChIP-seq) and long-read genome assemblies from multiple *Arabidopsis* genetic backgrounds, mutant systems and natural accessions. See the ‘Data availability’ section and Supplementary Table 25 for accession numbers.

#### Natural accessions

Raw WGBS data for 927 samples were downloaded^3^. SRA files were converted to FASTQ format using fastq-dump (v2.9.2). For samples with multiple FASTQ files associated with the same GSM accession, individual BAM files were first generated and then merged using ‘samtools merge -n’ (Samtools v1.11). Only samples derived from leaf tissue were retained. To account for redundant accessions, we selected the sample (GSM ID) with the highest sequencing depth when multiple samples corresponded to the same ecotype ID. This filtering resulted in a final set of 850 unique accessions (Supplementary Table 1).

#### MA accessions

We previously generated MA lines using four different *A. thaliana* natural ecotypes as founders^17^. Further details concerning the construction of these pedigrees and DNA extraction protocols are provided in the original publication^17^.

#### Cvi accessions

WGBS data and transcriptome data were obtained from Zicola et al.^41^.

#### Col × Cvi RIL population and Col-0 and Cvi-0 accessions

Published WGBS and transcriptome data were obtained from Picard and Gehring^56^.

#### H3K27me3 chromatin and mutant data

H3K27me3 ChIP-seq signal tracks for Col-0 were downloaded from the Plant Epigenome Browser (https://epigenome.genetics.uga.edu; Schmitz Lab, University of Georgia), on the basis of data generated by Lu et al.^54^. To assess the functional contribution of H3K27me3 to *VIM2*/*4* regulation, published RNA-seq data for H3K27me3-deficient *clf swn* double mutants and their corresponding wild-type controls were obtained from Mozgová et al.^55^.

#### *vim* and *met1* mutants

Published WGBS data for *vim1*, *vim2*, *vim3*, *vim1/2/3*, *met1* and their corresponding Col-0 wild-type controls were obtained from Stroud et al.^7^. Published RNA-seq data for *vim1*, *vim1/2/3* and *met1* mutants and their corresponding Col-0 wild-type controls were obtained from Shook and Richards^57^.

#### Long-read genome assemblies

Published long-read assemblies for Ler-0 and Cvi-0 were obtained from Wlodzimierz et al.^49^ and Baduel et al.^48^, and an additional Cvi-0 assembly was obtained from Lian et al.^50^, together with the Col-0 reference genome (TAIR10)^82^.

#### SNP data

Polymorphisms between Ler-0 and Cvi-0 were obtained from the 1001 *Arabidopsis* Genomes project^53^ and used to cross-reference SNPs identified from WGBS for genetic map construction (see the ‘Identification of SNP markers from WGBS for genetic mapping’ section).

#### *ros1* mutant DMR data

Canonical ROS1 target regions were defined on the basis of DMR analysis of *ros1* mutants relative to wild type from Zhang et al.^63^.

### WGBS data processing

To ensure consistent processing across multiple *Arabidopsis* WGBS datasets, including our newly generated MA-ML, *VIM2*/*4* overexpression lines and mutants, as well as previously published datasets such as natural accessions and MA accessions, we used the MethylStar pipeline^83,84^ with TAIR10^82^ as the reference genome. MethylStar is a fast and robust preprocessing pipeline for bulk WGBS data^84^. Cytosine-level methylation states were inferred using METHimpute^83^, which classifies each site into one of three states: unmethylated (‘U’), intermediate (‘I’) or methylated (‘M’), on the basis of a three-state hidden Markov model. It is capable of making accurate methylation calls even for cytosines with missing or low read counts. Another advantage of METHimpute is its ability to identify not only epihomozygous unmethylated (UU) or epihomozygous methylated sites (MM) but also epiheterozygous sites (MU). Knowledge of epiheterozygous sites is necessary when studying epimutation accumulation in clonal species/lineages, as most epimutations are of the form UU ↔ MU or MM ↔ MU. To ensure high-quality methylation state calls, only cytosines with a maximum posterior probability larger than 0.99 were retained. Sequencing metrics for each MA-ML sample, including sequencing depth, mapping efficiency and coverage, are presented in Supplementary Table 2. The same metrics for the *VIM2*/*4* overexpression lines and mutants are presented in Supplementary Table 20.

### Enrichment of annotations

Gene and TE annotation files in GFF3 format were downloaded from Ensembl Plants^85^. The gbM, teM and LM gene sets were obtained from previous work^86,87^. In this study, genes previously labelled as unmethylated (UM) were referred to as lowly methylated (LM), following Zhang et al.^63^. The ‘other_gene’ category was defined by subtracting gbM, teM and LM genes from the total gene set. Intergenic regions were defined as genomic intervals not overlapping with genes, TEs or promoter regions, using bedtools complement^18,88^. The ‘others’ category includes genomic regions not assigned to any of the above annotations. The genome coordinates and corresponding epigenetic marks for the 36 CSs were obtained from the PCSD database^42^. Clustering of these CSs into four major groups was performed according to Hazarika et al.^18^, corresponding to green, red, blue and purple states.

### Calculation of average methylation levels

Average observed methylation levels were computed using methylome data, considering only sites with posteriorMax ≥0.99 to ensure high-confidence calls. The proportions of unmethylated (U) and intermediate (I) states were calculated relative to the total number of high-confidence sites (*n*_total). The methylation level was then defined as 1 − *n*_U_/*n*_total − 0.5 × *n*_I_/*n*_total, where *n*_U_ and *n*_I_ represent the number of sites classified as unmethylated and intermediate states, respectively. Predicted methylation levels were obtained by applying the mathematical model formulated by van der Graaf et al.^22^, which relates steady-state methylation to the gain and loss rates.

### Metaplot visualization of DNA methylation profiles

DNA methylation profiles across genes and TEs were visualized using scaled metaplots. Each feature body was divided into 20 equally proportioned windows, and 1,000-bp upstream and downstream flanking regions were each divided into 10 windows of 100 bp. For each window, the mean recalibrated methylation level (derived from METHimpute posterior probabilities, posteriorMax ≥0.99) was averaged across all features within the same annotation group. Plots were generated separately for CG, CHG and CHH contexts using the R package ggplot2.

### Estimation of epimutation rates

To obtain accurate estimates of epimutation rates in the MA-ML and MA-accessions pedigrees, global and specific annotated estimates of the CG epimutation gain rate (*α*) and the loss rate (*β*) at the level of individual cytosines were acquired using the R package AlphaBeta (version 1.10.0)^19^. AlphaBeta is a generalization of our previous modelling-based approach for estimating epimutation rates in self-derived MA lines^22^. AlphaBeta takes pedigree-based multigenerational WGBS data as input and output estimates of the unknown gain (*α*) and loss rates (*β*), along with their CIs. To quantify mCG divergence between samples, we applied the divergence estimation function implemented in the AlphaBeta package, which leverages pedigree structure to model changes in methylation states. The underlying methodology and mathematical framework are detailed in the original publication^19^. For all analyses, we fitted the neutral model (‘ABneutral’). AlphaBeta estimates gain and loss rates from the observed state transitions while accounting for measurement error and finite-generation dynamics. A key advantage of this framework is that the inferred rates can be used to predict steady-state mCG levels, providing an internal consistency check that simple transition counts do not provide.

For comparison, we also applied a simpler count-based method to estimate epimutation rates on MA-ML. Our pipeline categorizes methylation states into methylated (M) and unmethylated (U), excluding intermediate (I) states. To quantify epimutation rates, we tracked methylation state transitions between early and late generations (that is, G2 and G9) within the main lineage. Methylation gain was assessed by calculating the ratio of U to M transitions, defined as the number of sites that changed from U to M relative to the total number of U sites in the early generation. Similarly, methylation loss was quantified by the ratio of M to U transitions, calculated as the number of sites transitioning from M to U relative to the total M sites in the early generation. Although the count-based approach successfully maps the major-effect QTL for methylation to the same locus on chromosome 1 as AlphaBeta, we show the critical advantages of the AlphaBeta computational framework in robustly predicting steady-state methylation levels and capturing epimutation rates in Supplementary Figs. 10 and 11.

### Identification of SNP markers from WGBS for genetic mapping

To construct a genetic map for Ler × Cvi RILs, we identified polymorphic SNPs from WGBS data of MA-ML. BAM files were first extracted from the bismark-deduplicate output generated by Methylstar^83,84^, followed by sorting and indexing with samtools (v1.17). Genome-wide SNPs were identified using bcftools (v1.17) with default settings, using TAIR10 as the reference genome while excluding indels. To retain informative parental polymorphisms, we cross-referenced SNPs identified from WGBS with polymorphisms between Ler-0 and Cvi-0 obtained from the 1001 *Arabidopsis* Genomes project^53^. Only SNPs that overlapped between these datasets were retained.

We subsequently retained homozygous biallelic SNPs, required consistent genotypes across all samples from the same RIL and restricted SNP occurrence frequencies to 30–70% across RILs to exclude rare or nearly fixed variants. To avoid ambiguity caused by bisulfite-induced C-to-T conversion, only A/G SNPs were retained.

To reduce marker redundancy and noise, SNPs were summarized using sliding windows of 200 SNPs with a 5-SNP step. Windows containing >50% Ler SNPs were classified as Ler, those containing <50% Ler SNPs as Cvi and windows with equal Ler and Cvi proportions as missing data. This yielded 12,479 window-based markers.

Further refinement was performed using the qtl R package (v1.70)^89^, where redundant markers were removed with the ‘findDupMarkers’ function, and markers with Ldiff >2 were excluded using the ‘droponemarker’ function to improve map quality. This resulted in a final set of 732 SNP markers (Supplementary Table 4), which were subsequently used to construct a high-density genetic map.

### QTL mapping approach

Using DNA methylation levels as molecular quantitative traits, we performed meQTL. Treating the genotype-specific estimates for the gain (*α*) and loss rate (*β*) as molecular quantitative traits, we performed a search for QTL (that is, *αβ*-QTL) underlying rate variation using classical linkage analysis. QTL scans were performed for genome-wide as well as annotation-specific epimutation rates. We re-analysed the molecular data and searched for specific linkage association with our *αβ*-QTL and meQTL. This approach can uncover genes and their products that act in candidate pathways or co-expression networks^90^. Owing to the high sequence similarity and close physical proximity (~2.6 kb apart) between the paralogous genes *VIM2* and *VIM4*, their transcripts are difficult to reliably distinguish by RNA-seq. As a result, the raw eQTL dataset reports expression values only for *VIM4*, which probably reflect the combined expression of both *VIM2* and *VIM4* (hereafter referred to as *VIM2*/*4*).

QTL mapping was performed using the qtl R package (v1.70) to identify loci associated with epigenetic variation^89^. The genetic map was constructed using est.map(), incorporating an optimized error probability of 1 × 10^−7^ for improved accuracy. Single-QTL genome scans were conducted using Haley–Knott regression for multiple phenotypic traits, including global and annotation-specific methylation levels and epimutation rates. Significance thresholds (*α* = 0.05) were determined via 1,000 permutations. LOD CIs were defined using a drop threshold of 2.0 via the ‘lodint()’ function, and the most significant QTL peak LOD for each trait was identified.

### Calling Cvi- and Ler-derived chromosomal segments

To define genomic segments inherited by the Ler × Cvi RILs, we classified each genomic region into Ler segments and Cvi segments on the basis of the previously mentioned 12,479 SNP markers, which exclusively comprised Cvi and Ler genotypes. For each RIL, markers assigned to Ler were grouped into contiguous segments. The start and end positions of each Ler segment were determined using the first and last marker of the segment, with the midpoint taken as the representative position. Cvi segments were identified independently using the same approach, on the basis of markers assigned to Cvi.

### DMR calling

DMRs were identified using the jDMR pipeline (https://github.com/jlab-code/jDMRgrid), adapted from the protocol described by Hazarika et al.^18^. The genome was partitioned into 200-bp sliding windows with a 50-bp step size, retaining only those bins containing a minimum of ten cytosines. For each sequence context (CG, CHG and CHH), per-window methylation states (methylated or unmethylated) were inferred using METHimpute^83^. We then constructed a comprehensive methylation call matrix to facilitate pairwise comparisons between the wild-type and treatment groups. Finally, invariant methylation patterns across samples were filtered out and contiguous differentially methylated bins were merged to generate the DMR set.

### RNA-seq data processing

RNA-seq data were newly generated for high- and low-epimutation-rate Ler × Cvi RILs, *VIM2*/*4* overexpression lines, *vim2* and *vim4* T-DNA insertion mutants and Col-0 wild-type controls as described in the respective sections above. All libraries were sequenced on an Illumina platform to generate 150-bp paired-end reads. When multiple FASTQ files existed for the same sample, they were concatenated before downstream processing. Raw reads were quality-checked with FastQC (v0.11.9) and trimmed using Trimmomatic (v0.38). Trimmed reads were aligned to the TAIR10 reference genome using STAR (v2.7.6a), and alignments were sorted and indexed using SAMtools (v1.17). Gene-level read counts were obtained using HTSeq-count (v0.13.5) with the Araport11 annotation.

In addition, published RNA-seq data for *vim1*, *vim1/2/3* and *met1* mutants from Shook and Richards^57^ were re-analysed using the same pipeline. For published datasets of H3K27me3-deficient *clf swn* double mutants from Mozgová et al.^55^, Col-0 and Cvi-0 accessions, Col × Cvi RILs from Picard et al.^56^ and natural Cvi accessions from Zicola et al.^41^, precomputed gene-level raw count matrices were obtained from the original publications and re-analysed from the count stage.

All count matrices were restricted to nuclear genes on chromosomes 1–5 and analysed separately using DESeq2 v1.30.1. Counts were regularized-log transformed with rlog for visualization. For each comparison, log_2_ fold changes and nominal two-sided Wald-test *P* values for candidate DNA methylation pathway genes were extracted from the DESeq2 output. As these genes were selected a priori on the basis of their established roles in DNA methylation maintenance and demethylation pathways, significance was assessed using nominal *P* values (*P* < 0.05), whereas genome-wide comparisons used the Benjamini–Hochberg false discovery rate (FDR <0.05).

To quantify gene expression divergence across generations of RILs using the same pedigree-based framework applied to methylation data, we converted rlog-normalized gene expression values from DESeq2 into a discrete gene-level state matrix compatible with AlphaBeta. For each RNA-seq sample, expression values were minimum–maximum scaled to an interval of (0, 1). Genes were then discretized into three ordinal states on the basis of within-sample tertile thresholds. Genes in the lowest tertile were assigned to the low-expression state U, genes in the middle tertile to the intermediate-expression state I and genes in the highest tertile to the high-expression state M. This three-state encoding mirrors the methylation state calls from METHimpute and enables direct application of AlphaBeta to each MA-ML pedigree. Subsequently, gene-level state files were generated containing chromosome, start, end, the assigned expression state and the scaled expression value. We then applied AlphaBeta independently to each MA-ML pedigree. For each RIL, the neutral accumulation model (ABneutral) was fitted and compared with the null model of no accumulation (ABnull) using the built-in *F*-test for the statistical significance of transcriptome divergence accumulation over time.

### ATAC-seq data processing

Sequencing reads were processed using a standard ATAC-seq pipeline as described below. Adapters were trimmed using Trimmomatic v0.39, and paired-end reads were aligned to the TAIR10 genome using BWA-MEM (v0.7.17). Sorted BAM files were deduplicated using Picard MarkDuplicates (v2.23.8; REMOVE_DUPLICATES=true). Peaks were called with MACS2 (v2.2.9.1) using BEDPE input mode and *Arabidopsis* genome size (1.35 × 10^8^), with parameters keep-dup all, SPMR and -q 0.05. All downstream analyses were restricted to chromosomes 1–5.

To quantify chromatin accessibility divergence across generations, we converted ATAC-seq peak calls into a genome-wide binary presence/absence matrix. The genome was divided into non-overlapping 100-bp bins. Each bin was scored as ‘M’ (accessible) in a sample if at least 50% of its length overlapped with a called ATAC-seq peak and as ‘U’ (inaccessible) otherwise. This binarization approach mirrors the construction of the methylation matrix from WGBS data. All bins were retained regardless of accessibility to ensure full genome coverage. Chromatin accessibility divergence between samples across generations was inferred using AlphaBeta^19^, with model fitting and significance testing as described for the RNA-seq data.

### Genotyping and qPCR of MA-ML

Detailed genotyping and qPCR procedures, including primer information and expression quantification, are presented in Supplementary Table 24 (ref. ^91^).

### Reporting summary

Further information on research design is available in the Nature Portfolio Reporting Summary linked to this article.

## Supplementary information

Supplementary Information Supplementary Figs. 1–11.

Reporting Summary

Supplementary Tables Supplementary Tables 1–25.

## Source data

Source Data Fig. 1 Graph data.

Source Data Fig. 2 Graph data.

Source Data Fig. 4 Unprocessed gels and statistical source data.

Source Data Fig. 5 Graph data.

Source Data Extended Data Fig./Table 5 Unprocessed gels and statistical source data.

Source Data Extended Data Fig./Table 6 Graph data.

Source Data Extended Data Fig./Table 9 Graph data.

## Data Availability

All sequencing data newly generated in this study have been deposited in the National Center for Biotechnology Information (NCBI) Gene Expression Omnibus (GEO). The whole-genome bisulfite sequencing (WGBS) data, including 371 mutation accumulation mapping lines (MA-ML) samples for 68 Ler × Cvi RILs and 2 parental MA-lines (Ler-0 and Cvi-0), have been deposited in the NCBI GEO under accession no. GSE296957. WGBS data for Col-0 wild-type controls; *vim2* and *vim4* single mutants; and *VIM2*, *VIM4* and *VIM24* overexpression lines (Col-0 and Cvi-0 alleles) in the Col-0 background have been deposited under accession no. GSE333205. RNA-seq data for Col-0 wild-type controls; *vim2* and *vim4* single mutants; *VIM2*, *VIM4* and *VIM24* overexpression lines; and six Ler × Cvi RILs selected for high or low epimutation rates have been deposited under accession no. GSE333206. ATAC-seq data for two selected RILs have been deposited under accession no. GSE297077. Previously published datasets used in this study were obtained from the following sources. MA-accessions methylome data are available under GSE223810^17^. Cvi accessions data were available in NCBI Sequence Read Archive (SRA) under BioProject no. PRJNA612437^41^. Previously published WGBS data for natural accessions were downloaded from GEO accession no. GSE43857^3^, and corresponding transcriptome data were obtained from GSE80744^3^. eQTL in the Ler × Cvi RILs population were obtained from previously published datasets^34,35^. WGBS and transcriptome data for the Col × Cvi RILs population and Col-0 and Cvi-0 accessions were obtained from GEO accession no. GSE52814^56^. RNA-seq data for H3K27me3-deficient mutants and corresponding wild-type controls were obtained from GEO accession no. GSE81166^55^. Long-read genome assemblies for Ler-0 and Cvi-0 were obtained from European Nucleotide Archive (ENA) projects PRJEB77668^48^ and PRJEB55353^49^, with an additional Cvi-0 assembly retrieved from NCBI BioProject PRJNA1033522^50^. WGBS data for *vim1*, *vim2*, *vim3*, *vim1/2/3*, *met1* and corresponding Col-0 wild-type controls were obtained from GEO accession no. GSE39901^7^. RNA-seq data for *vim1*, *vim1/2/3* and *met1* mutants and corresponding Col-0 wild-type controls were obtained from NCBI BioProject PRJNA238327^57^. An overview of all datasets used in this study and their sources is presented in Supplementary Table 25. Source data are provided with this paper.

## Code availability

The codes for this study are available via GitHub at https://github.com/zhilinzhang340/Mapping-genetic-modifiers-of-epimutation-rates-codes-Zhilin_Zhang and via Zenodo at https://doi.org/10.5281/zenodo.20387093 (ref. ^92^).

## References

[1] Law, J. A. & Jacobsen, S. E. Establishing, maintaining and modifying DNA methylation patterns in plants and animals. *Nat. Rev. Genet.* **11**, 204–220 (2010). 10.1038/nrg2719 PMC3034103 20142834

[2] Schmitz, R. J. et al. Patterns of population epigenomic diversity. *Nature* **495**, 193–198 (2013). 10.1038/nature11968 PMC3798000 23467092

[3] Kawakatsu, T. et al. Epigenomic diversity in a global collection of *Arabidopsis thaliana* accessions. *Cell* **166**, 492–505 (2016). 10.1016/j.cell.2016.06.044 PMC5172462 27419873

[4] Zhao, T. et al. Population-wide DNA methylation polymorphisms at single-nucleotide resolution in 207 cotton accessions reveal epigenomic contributions to complex traits. *Cell Res.* **34**, 859–872 (2024). 10.1038/s41422-024-01027-x PMC11615300 39420233

[5] Cortijo, S. et al. Mapping the epigenetic basis of complex traits. *Science* **343**, 1145–1148 (2014). 10.1126/science.1248127 24505129

[6] Song, X. et al. Inheritance of acquired adaptive cold tolerance in rice through DNA methylation. *Cell* **188**, 4213–4224 (2025). 10.1016/j.cell.2025.04.036 40409269

[7] Stroud, H., Greenberg, M. V. C., Feng, S., Bernatavichute, Y. V. & Jacobsen, S. E. Comprehensive analysis of silencing mutants reveals complex regulation of the *Arabidopsis* methylome. *Cell* **152**, 352–364 (2013). 10.1016/j.cell.2012.10.054 PMC3597350 23313553

[8] Woo, H. R., Pontes, O., Pikaard, C. S. & Richards, E. J. VIM1, a methylcytosine-binding protein required for centromeric heterochromatinization. *Genes Dev.* **21**, 267–277 (2007). 10.1101/gad.1512007 PMC1785122 17242155

[9] Woo, H. R., Dittmer, T. A. & Richards, E. J. Three SRA-domain methylcytosine-binding proteins cooperate to maintain global CpG methylation and epigenetic silencing in *Arabidopsis*. *PLoS Genet.* **4**, e1000156 (2008). 10.1371/journal.pgen.1000156 PMC2491724 18704160

[10] Kim, J., Kim, J. H., Richards, E. J., Chung, K. M. & Woo, H. R. *Arabidopsis* VIM proteins regulate epigenetic silencing by modulating DNA methylation and histone modification in cooperation with MET1. *Mol. Plant* **7**, 1470–1485 (2014). 10.1093/mp/ssu079 PMC4207863 25009302

[11] Feng, S. et al. Conservation and divergence of methylation patterning in plants and animals. *Proc. Natl Acad. Sci. USA* **107**, 8689–8694 (2010). 10.1073/pnas.1002720107 PMC2889301 20395551

[12] Bostick, M. et al. UHRF1 plays a role in maintaining DNA methylation in mammalian cells. *Science* **317**, 1760–1764 (2007). 10.1126/science.1147939 17673620

[13] Hashimoto, H. et al. The SRA domain of UHRF1 flips 5-methylcytosine out of the DNA helix. *Nature* **455**, 826–829 (2008). 10.1038/nature07280 PMC2602803 18772888

[14] Arita, K., Ariyoshi, M., Tochio, H., Nakamura, Y. & Shirakawa, M. Recognition of hemi-methylated DNA by the SRA protein UHRF1 by a base-flipping mechanism. *Nature* **455**, 818–821 (2008). 10.1038/nature07249 18772891

[15] Sharif, J. et al. The SRA protein Np95 mediates epigenetic inheritance by recruiting Dnmt1 to methylated DNA. *Nature* **450**, 908–912 (2007). 10.1038/nature06397 17994007

[16] Avvakumov, G. V. et al. Structural basis for recognition of hemi-methylated DNA by the SRA domain of human UHRF1. *Nature* **455**, 822–825 (2008). 10.1038/nature07273 18772889

[17] Yao, N. et al. An evolutionary epigenetic clock in plants. *Science* **381**, 1440–1445 (2023). 10.1126/science.adh9443 37769069

[18] Hazarika, R. R. et al. Molecular properties of epimutation hotspots. *Nat. Plants* **8**, 146–156 (2022). 10.1038/s41477-021-01086-7 PMC8866225 35087209

[19] Shahryary, Y. et al. AlphaBeta: computational inference of epimutation rates and spectra from high-throughput DNA methylation data in plants. *Genome Biol.* **21**, 260 (2020). 10.1186/s13059-020-02161-6 PMC7539454 33023650

[20] Becker, C. et al. Spontaneous epigenetic variation in the *Arabidopsis* thaliana methylome. *Nature* **480**, 245–249 (2011). 10.1038/nature10555 22057020

[21] Schmitz, R. J. et al. Transgenerational epigenetic instability is a source of novel methylation variants. *Science* **334**, 369–373 (2011). 10.1126/science.1212959 PMC3210014 21921155

[22] van der Graaf, A. et al. Rate, spectrum, and evolutionary dynamics of spontaneous epimutations. *Proc. Natl. Acad. Sci. USA* **112**, 6676–6681 (2015). 10.1073/pnas.1424254112 PMC4450394 25964364

[23] Briffa, A. et al. Millennia-long epigenetic fluctuations generate intragenic DNA methylation variance in *Arabidopsis* populations. *Cell Syst.* **14**, 953–967 (2023). 10.1016/j.cels.2023.10.007 37944515

[24] Johannes, F. & Schmitz, R. J. Spontaneous epimutations in plants. *New Phytol.* **221**, 1253–1259 (2019). 10.1111/nph.15434 30216456

[25] Yao, N., Schmitz, R. J. & Johannes, F. Epimutations define a fast-ticking molecular clock in plants. *Trends Genet.* **37**, 699–710 (2021). 10.1016/j.tig.2021.04.010 PMC8282728 34016450

[26] Denkena, J., Johannes, F. & Colomé-Tatché, M. Region-level epimutation rates in *Arabidopsis thaliana*. *Heredity* **127**, 190–202 (2021). 10.1038/s41437-021-00441-w PMC8322157 33966050

[27] Goeldel, C. & Johannes, F. Stochasticity in gene body methylation. *Curr. Opin. Plant Biol.* **75**, 102436 (2023). 10.1016/j.pbi.2023.102436 37597469

[28] Taudt, A., Colomé-Tatché, M. & Johannes, F. Genetic sources of population epigenomic variation. *Nat. Rev. Genet.* **17**, 319–332 (2016). 10.1038/nrg.2016.45 27156976

[29] Dubin, M. J. et al. DNA methylation in *Arabidopsis* has a genetic basis and shows evidence of local adaptation. *eLife* **4**, e05255 (2015). 10.7554/eLife.05255 PMC4413256 25939354

[30] Sasaki, E., Gunis, J., Reichardt-Gomez, I., Nizhynska, V. & Nordborg, M. Conditional GWAS of non-CG transposon methylation in *Arabidopsis thaliana* reveals major polymorphisms in five genes. *PLoS Genet.* **18**, e1010345 (2022). 10.1371/journal.pgen.1010345 PMC9491579 36084135

[31] Lindroth, A. M. et al. Requirement of CHROMOMETHYLASE3 for maintenance of CpXpG methylation. *Science* **292**, 2077–2080 (2001). 10.1126/science.1059745 11349138

[32] Wendte, J. M. et al. Epimutations are associated with CHROMOMETHYLASE 3-induced de novo DNA methylation. *eLife* **8**, e47891 (2019). 10.7554/eLife.47891 PMC6663294 31356150

[33] Alonso-Blanco, C. et al. Development of an AFLP based linkage map of Ler, Col and Cvi *Arabidopsis thaliana* ecotypes and construction of a Ler/Cvi recombinant inbred line population. *Plant J.* **14**, 259–271 (1998). 10.1046/j.1365-313x.1998.00115.x 9628021

[34] Fu, J. et al. System-wide molecular evidence for phenotypic buffering in *Arabidopsis*. *Nat. Genet.* **41**, 166–167 (2009). 10.1038/ng.308 19169256

[35] Keurentjes, J. J. B. et al. Regulatory network construction in *Arabidopsis* by using genome-wide gene expression quantitative trait loci. *Proc. Natl Acad. Sci. USA* **104**, 1708–1713 (2007). 10.1073/pnas.0610429104 PMC1785256 17237218

[36] Mackay, I. J., Cockram, J., Howell, P. & Powell, W. Understanding the classics: the unifying concepts of transgressive segregation, inbreeding depression and heterosis and their central relevance for crop breeding. *Plant Biotechnol. J.* **19**, 26–34 (2021). 10.1111/pbi.13481 PMC7769232 32996672

[37] Reinders, J. et al. Compromised stability of DNA methylation and transposon immobilization in mosaic *Arabidopsis* epigenomes. *Genes Dev.* **23**, 939 (2009). 10.1101/gad.524609 PMC2675864 19390088

[38] Kakutani, T., Jeddeloh, J. A., Flowers, S. K., Munakata, K. & Richards, E. J. Developmental abnormalities and epimutations associated with DNA hypomethylation mutations. *Proc. Natl Acad. Sci. USA* **93**, 12406–12411 (1996). 10.1073/pnas.93.22.12406 PMC38004 8901594

[39] Kankel, M. W. et al. *Arabidopsis* MET1 cytosine methyltransferase mutants. *Genetics* **163**, 1109–1122 (2003). 10.1093/genetics/163.3.1109 PMC1462485 12663548

[40] Zhong, Z. et al. DNA methylation-linked chromatin accessibility affects genomic architecture in *Arabidopsis*. *Proc. Natl Acad. Sci. USA* **118**, e2023347118 (2021). 10.1073/pnas.2023347118 PMC7865151 33495321

[41] Zicola, J. et al. A VIM2/4 deletion, and premature truncations of *CMT2* and *FBX5*, drive DNA methylation changes after colonization of a novel habitat. *Nat. Plants* **12**, 1469–1484 (2026). 10.1038/s41477-026-02352-2 PMC13481252 42547832

[42] Liu, Y. et al. PCSD: a plant chromatin state database. *Nucleic Acids Res.* **46**, D1157–D1167 (2018). 10.1093/nar/gkx919 PMC5753246 29040761

[43] Vo, B. T. et al. Somatic DNA methylation heterogeneity predicts extreme transgenerational epimutation hotspots in *Arabidopsis*. Preprint at *bioRxiv* 10.64898/2026.04.04.716457 (2026).

[44] Muyle, A., Ross-Ibarra, J., Seymour, D. K. & Gaut, B. S. Gene body methylation is under selection in *Arabidopsis thaliana*. *Genetics* **218**, iyab061 (2021). 10.1093/genetics/iyab061 PMC8225343 33871638

[45] Bewick, A. J., Zhang, Y., Wendte, J. M., Zhang, X. & Schmitz, R. J. Evolutionary and experimental loss of gene body methylation and its consequence to gene expression. *G3* **9**, 2441–2445 (2019). 10.1534/g3.119.400365 PMC6686912 31147389

[46] Waese, J. et al. EPlant: visualizing and exploring multiple levels of data for hypothesis generation in plant biology. *Plant Cell* **29**, 1806–1821 (2017). 10.1105/tpc.17.00073 PMC5590499 28808136

[47] Klepikova, A. V., Kasianov, A. S., Gerasimov, E. S., Logacheva, M. D. & Penin, A. A. A high resolution map of the *Arabidopsis thaliana* developmental transcriptome based on RNA-seq profiling. *Plant J.* **88**, 1058–1070 (2016). 10.1111/tpj.13312 27549386

[48] Baduel, P. et al. Transposable elements are vectors of recurrent transgenerational epigenetic inheritance. *Science* **390**, eady3475 (2025). 10.1126/science.ady3475 40966307

[49] Wlodzimierz, P. et al. Cycles of satellite and transposon evolution in *Arabidopsis* centromeres. *Nature* **618**, 557–565 (2023). 10.1038/s41586-023-06062-z 37198485

[50] Lian, Q. et al. A pan-genome of 69 *Arabidopsis thaliana* accessions reveals a conserved genome structure throughout the global species range. *Nat. Genet.* **56**, 982–991 (2024). 10.1038/s41588-024-01715-9 PMC11096106 38605175

[51] Kang, M. et al. The pan-genome and local adaptation of *Arabidopsis thaliana*. *Nat. Commun.* **14**, 6259 (2023). 10.1038/s41467-023-42029-4 PMC10558531 37802986

[52] Igolkina, A. A. et al. A comparison of 27 *Arabidopsis thaliana* genomes and the path toward an unbiased characterization of genetic polymorphism. *Nat. Genet.* **57**, 2289–2301 (2025). 10.1038/s41588-025-02293-0 PMC12425826 40830656

[53] 1001 Genomes Consortium. 1,135 genomes reveal the global pattern of polymorphism in *Arabidopsis thaliana*. *Cell* **166**, 481–491 (2016). 10.1016/j.cell.2016.05.063 PMC4949382 27293186

[54] Lu, Z. et al. The prevalence, evolution and chromatin signatures of plant regulatory elements. *Nat. Plants* **5**, 1250–1259 (2019). 10.1038/s41477-019-0548-z 31740772

[55] Mozgová, I., Muñoz-Viana, R. & Hennig, L. PRC2 represses hormone-induced somatic embryogenesis in vegetative tissue of *Arabidopsis thaliana*. *PLoS Genet.* **13**, e1006562 (2017). 10.1371/journal.pgen.1006562 PMC5283764 28095419

[56] Picard, C. L. & Gehring, M. Proximal methylation features associated with nonrandom changes in gene body methylation. *Genome Biol.* **18**, 73 (2017). 10.1186/s13059-017-1206-2 PMC5406939 28446217

[57] Shook, M. S. & Richards, E. J. VIM proteins regulate transcription exclusively through the MET1 cytosine methylation pathway. *Epigenetics* **9**, 980–986 (2014). 10.4161/epi.28906 PMC4143413 24762702

[58] Hsieh, T.-F. et al. Regulation of imprinted gene expression in *Arabidopsis* endosperm. *Proc. Natl Acad. Sci. USA* **108**, 1755–1762 (2011). 10.1073/pnas.1019273108 PMC3033266 21257907

[59] Gehring, M., Missirian, V. & Henikoff, S. Genomic analysis of parent-of-origin allelic expression in *Arabidopsis thaliana* seeds. *PLoS ONE* **6**, e23687 (2011). 10.1371/journal.pone.0023687 PMC3157454 21858209

[60] Szklarczyk, D. et al. The STRING database in 2023: protein–protein association networks and functional enrichment analyses for any sequenced genome of interest. *Nucleic Acids Res.* **51**, D638–D646 (2023). 10.1093/nar/gkac1000 PMC9825434 36370105

[61] Williams, B. P., Pignatta, D., Henikoff, S. & Gehring, M. Methylation-sensitive expression of a DNA demethylase gene serves as an epigenetic rheostat. *PLoS Genet.* **11**, e1005142 (2015). 10.1371/journal.pgen.1005142 PMC4380477 25826366

[62] Lei, M. et al. Regulatory link between DNA methylation and active demethylation in *Arabidopsis*. *Proc. Natl Acad. Sci. USA* **112**, 3553–3557 (2015). 10.1073/pnas.1502279112 PMC4371987 25733903

[63] Zhang, Z. et al. Multi-generational biotic stress increases the rate of spontaneous epimutations in a ROS1-dependent manner. Preprint at *bioRxiv* 10.1101/2024.09.19.613880 (2024).

[64] Pisupati, R., Nizhynska, V., Mollá Morales, A. & Nordborg, M. On the causes of gene-body methylation variation in *Arabidopsis thaliana*. *PLoS Genet.* **19**, e1010728 (2023). 10.1371/journal.pgen.1010728 PMC10187938 37141384

[65] Shin, S.-Y. et al. Genome-wide CG hypomethylation of the *Arabidopsis* ecotype Cvi linked to structural variation and RNAi at the VIM4–VIM2 locus. *Proc. Natl Acad. Sci. USA* **123**, e2603682123 (2026). 10.1073/pnas.2603682123 PMC13213937 42154559

[66] Kraft, E., Bostick, M., Jacobsen, S. E. & Callis, J. ORTH/VIM proteins that regulate DNA methylation are functional ubiquitin E3 ligases. *Plant J.* **56**, 704–715 (2008). 10.1111/j.1365-313X.2008.03631.x PMC2973330 18643997

[67] Zhang, P. et al. PhyloGenes: an online phylogenetics and functional genomics resource for plant gene function inference. *Plant Direct* **4**, e00293 (2020). 10.1002/pld3.293 PMC7773024 33392435

[68] Thomas, P. D. et al. PANTHER: making genome-scale phylogenetics accessible to all. *Protein Sci.* **31**, 8–22 (2022). 10.1002/pro.4218 PMC8740835 34717010

[69] Jia, Y. et al. Negative regulation of DNMT3A de novo DNA methylation by frequently overexpressed UHRF family proteins as a mechanism for widespread DNA hypomethylation in cancer. *Cell Discov.* **2**, 16007 (2016). 10.1038/celldisc.2016.7 PMC4849474 27462454

[70] Ashraf, W. et al. The epigenetic integrator UHRF1: on the road to become a universal biomarker for cancer. *Oncotarget* **8**, 51946–51962 (2017). 10.18632/oncotarget.17393 PMC5584303 28881702

[71] Sidhu, H. & Capalash, N. UHRF1: the key regulator of epigenetics and molecular target for cancer therapeutics. *Tumour Biol.* **39**, 1010428317692205 (2017). 10.1177/1010428317692205 28218043

[72] Mudbhary, R. et al. UHRF1 overexpression drives DNA hypomethylation and hepatocellular carcinoma. *Cancer Cell* **25**, 196–209 (2014). 10.1016/j.ccr.2014.01.003 PMC3951208 24486181

[73] Du, Z. et al. DNMT1 stability is regulated by proteins coordinating deubiquitination and acetylation-driven ubiquitination. *Sci. Signal* **3**, ra80 (2010). 10.1126/scisignal.2001462 PMC3116231 21045206

[74] Kong, X. et al. Defining UHRF1 domains that support maintenance of human colon cancer DNA methylation and oncogenic properties. *Cancer Cell* **35**, 633–648 (2019). 10.1016/j.ccell.2019.03.003 PMC6521721 30956060

[75] Shahzad, Z. et al. Gene body methylation regulates gene expression and mediates phenotypic diversity in natural *Arabidopsis* populations. *Nat. Plants* **11**, 2084–2099 (2025). 10.1038/s41477-025-02108-4 PMC12537493 40940427

[76] Muyle, A. M., Seymour, D. K., Lv, Y., Huettel, B. & Gaut, B. S. Gene body methylation in plants: mechanisms, functions, and important implications for understanding evolutionary processes. *Genome Biol. Evol.* **14**, evac038 (2022). 10.1093/gbe/evac038 PMC8995044 35298639

[77] Urich, M. A., Nery, J. R., Lister, R., Schmitz, R. J. & Ecker, J. R. MethylC-seq library preparation for base-resolution whole-genome bisulfite sequencing. *Nat. Protoc.* **10**, 475–483 (2015). 10.1038/nprot.2014.114 PMC4465251 25692984

[78] Buenrostro, J. D., Giresi, P. G., Zaba, L. C., Chang, H. Y. & Greenleaf, W. J. Transposition of native chromatin for multimodal regulatory analysis and personal epigenomics. *Nat. Methods* **10**, 1213 (2013). 10.1038/nmeth.2688 PMC3959825 24097267

[79] Glenn, T. C. et al. Adapterama I: universal stubs and primers for 384 unique dual-indexed or 147,456 combinatorially-indexed Illumina libraries (iTru & iNext). *PeerJ* **7**, e7755 (2019). 10.7717/peerj.7755 PMC6791352 31616586

[80] Binder, A. et al. A modular plasmid assembly kit for multigene expression, gene silencing and silencing rescue in plants. *PLoS ONE* **9**, e88218 (2014). 10.1371/journal.pone.0088218 PMC3923767 24551083

[81] O’Malley, R. C., Barragan, C. C. & Ecker, J. R. A user’s guide to the *Arabidopsis* T-DNA insertion mutant collections. *Methods Mol. Biol.* **1284**, 323–342 (2015). 10.1007/978-1-4939-2444-8_16 PMC5215775 25757780

[82] Lamesch, P. et al. The Arabidopsis Information Resource (TAIR): improved gene annotation and new tools. *Nucleic Acids Res.* **40**, D1202–D1210 (2012). 10.1093/nar/gkr1090 PMC3245047 22140109

[83] Taudt, A. et al. METHimpute: imputation-guided construction of complete methylomes from WGBS data. *BMC Genomics* **19**, 444 (2018). 10.1186/s12864-018-4641-x PMC5992726 29879918

[84] Shahryary, Y., Hazarika, R. R. & Johannes, F. MethylStar: a fast and robust pre-processing pipeline for bulk or single-cell whole-genome bisulfite sequencing data. *BMC Genomics* **21**, 479 (2020). 10.1186/s12864-020-06886-3 PMC7359584 32660416

[85] FTP Download. *EnsemblPlants* http://plants.ensembl.org/info/data/ftp (2026).

[86] Zhang, Y. et al. Heterochromatin is a quantitative trait associated with spontaneous epiallele formation. *Nat. Commun.* **12**, 6958 (2021). 10.1038/s41467-021-27320-6 PMC8630088 34845222

[87] Zhang, Y., Wendte, J. M., Ji, L. & Schmitz, R. J. Natural variation in DNA methylation homeostasis and the emergence of epialleles. *Proc. Natl Acad. Sci. USA* **117**, 4874–4884 (2020). 10.1073/pnas.1918172117 PMC7060739 32071208

[88] Quinlan, A. R. & Hall, I. M. BEDTools: a flexible suite of utilities for comparing genomic features. *Bioinformatics* **26**, 841–842 (2010). 10.1093/bioinformatics/btq033 PMC2832824 20110278

[89] Broman, K. W., Wu, H., Sen, S. & Churchill, G. A. R/qtl: QTL mapping in experimental crosses. *Bioinformatics* **19**, 889–890 (2003). 10.1093/bioinformatics/btg112 12724300

[90] Jansen, R. C. & Nap, J. P. Genetical genomics: the added value from segregation. *Trends Genet.* **17**, 388–391 (2001). 10.1016/s0168-9525(01)02310-1 11418218

[91] Schmittgen, T. D. & Livak, K. J. Analyzing real-time PCR data by the comparative C(T) method. *Nat. Protoc.* **3**, 1101–1108 (2008). 10.1038/nprot.2008.73 18546601

[92] Zhang, Z. zhilinzhang340/Mapping-genetic-modifiers-of-epimutation-rates-codes-Zhilin_Zhang: code and workflow for Zhang et al. ‘Mapping genetic modifiers of epimutation rates’. *Zenodo*10.5281/zenodo.20387093 (2026).

