# Supplementary material for: Mapping genetic modifiers of epimutation rates identifies VIM2/4 as dosage-sensitive negative regulators of CG methylation maintenance
Source: Nat Plants. 2026 Sep 3;12(10):1976–94. doi: 10.1038/s41477-026-02396-4 (PMC13680270; doi:10.1038/s41477-026-02396-4)
Supplement: Supplementary file 1 [file 41477_2026_2396_MOESM1_ESM.pdf]

nature plants

Supplementary information https://doi.org/10.1038/s41477-026-02396-4

Mapping genetic modifiers of epimutation rates identifies *VIM2/4* as dosage-sensitive negative regulators of CG methylation maintenance

In the format provided by the authors and unedited

Table of Contents

Supplementary Information

Supplementary Figures

Supplementary Fig. 1 | Comparative visualization of genotype segments along chromosomes 1–5 using SNP-based and physical markers in the Ler × Cvi RILs population

Supplementary Fig. 2 | Tissue- and developmental-stage-specific expression of *VIM2/4* in *Arabidopsis thaliana*

Supplementary Fig. 3 | Expression levels of DNA methylation pathway genes in an independent Col × Cvi RIL population and parental accessions

Supplementary Fig. 4 | Non-linear dosage relationship between *VIM2/4* expression and global CG methylation levels

Supplementary Fig. 5 | Functional association network of VIM2 in *Arabidopsis thaliana*

Supplementary Fig. 6 | Per-gene expression changes of additional DNA methylation regulators across multiple experimental contexts

Supplementary Fig. 7 | Per-sample expression changes of DNA methylation regulators in individual *VIM2/4* overexpression lines and loss-of-function mutants

Supplementary Fig. 8 | DNA methylation profiles at the *VIM1* and *VIM3* genes across wild-type, *VIM2/4* overexpression lines, and loss-of-function mutants

Supplementary Fig. 9 | Gene tree of the UHRF/VIM family

Supplementary Fig. 10 | Comparison of QTL mapping results using CG epimutation rates estimated by AlphaBeta and count-based methods

Supplementary Fig. 11 | Comparison of observed and predicted global mCG levels using epimutation rates from AlphaBeta and count-based models

Supplementary Tables

Supplementary Tables 1–25 are provided separately as an Excel file.

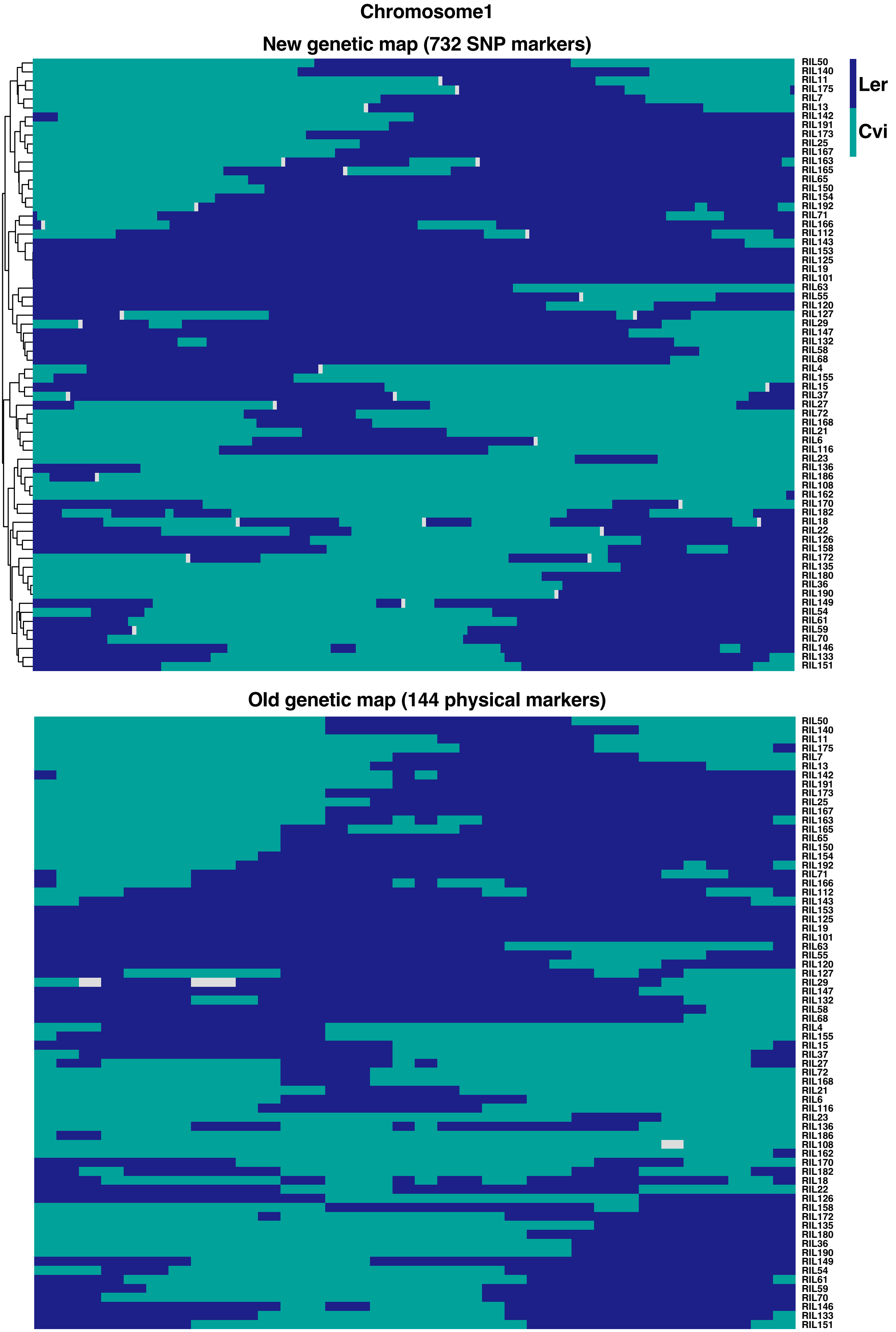

Supplementary Fig. 1

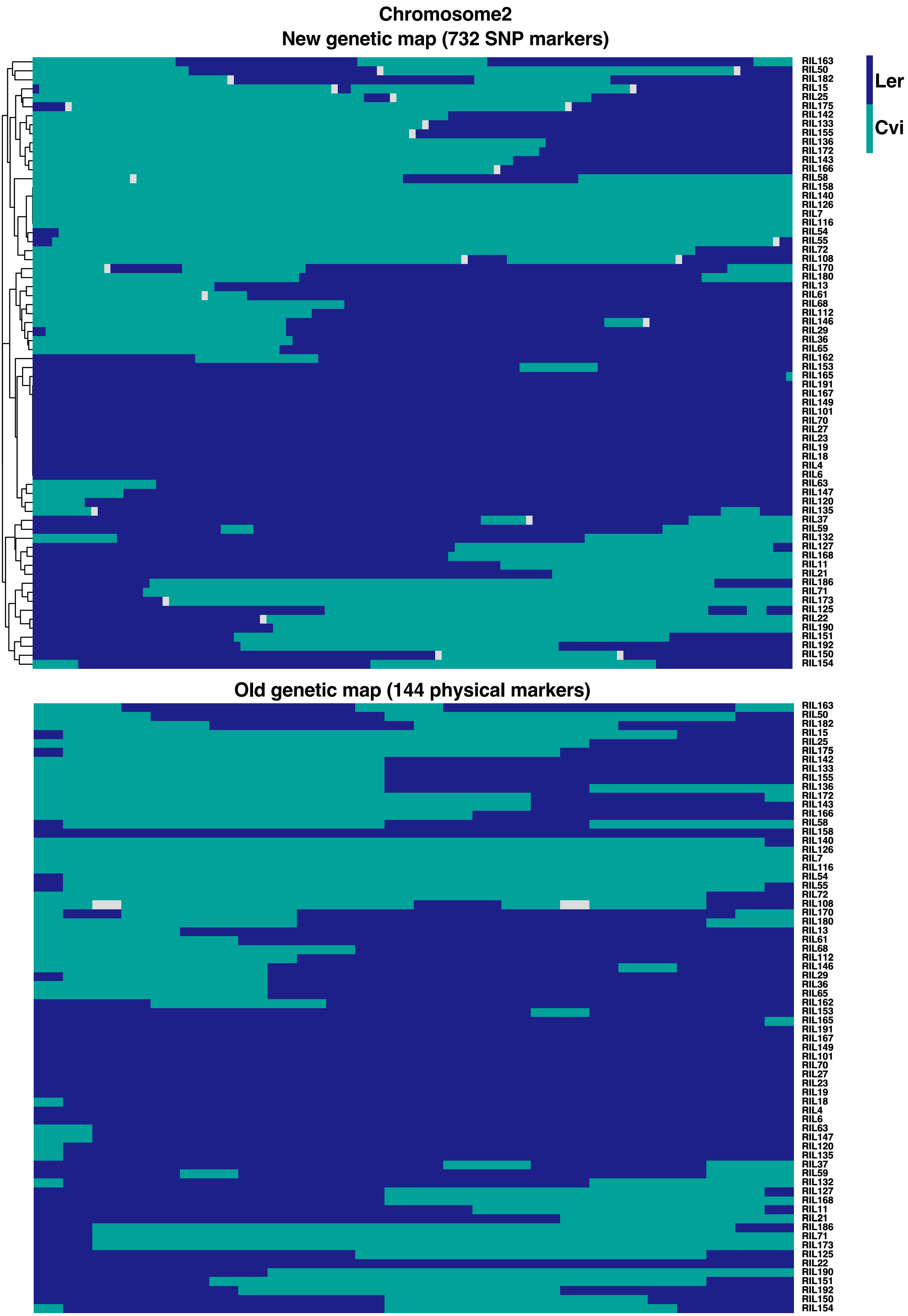

Supplementary Fig. 1

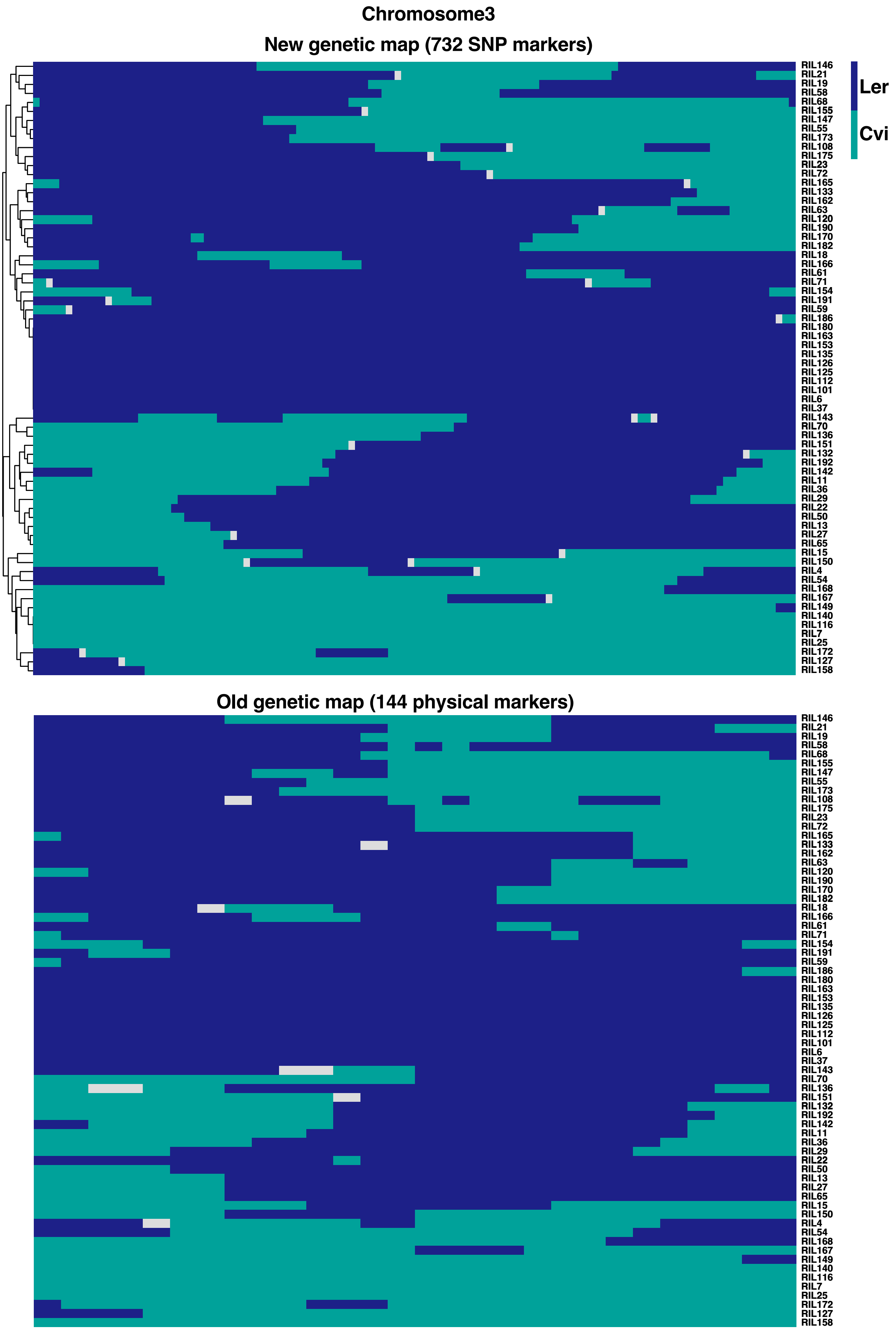

Supplementary Fig. 1

Chromosome4

New genetic map (732 SNP markers)

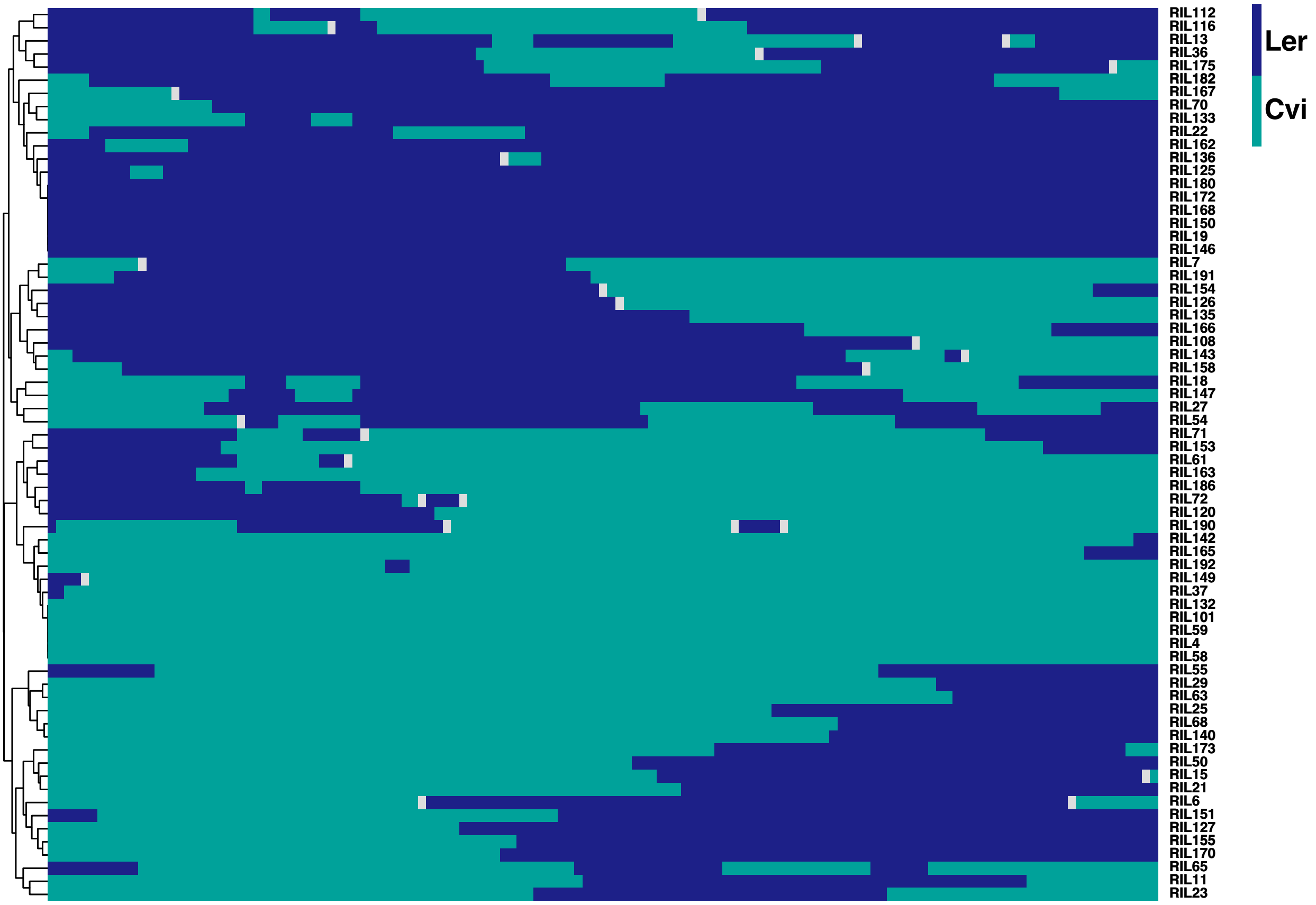

Old genetic map (144 physical markers)

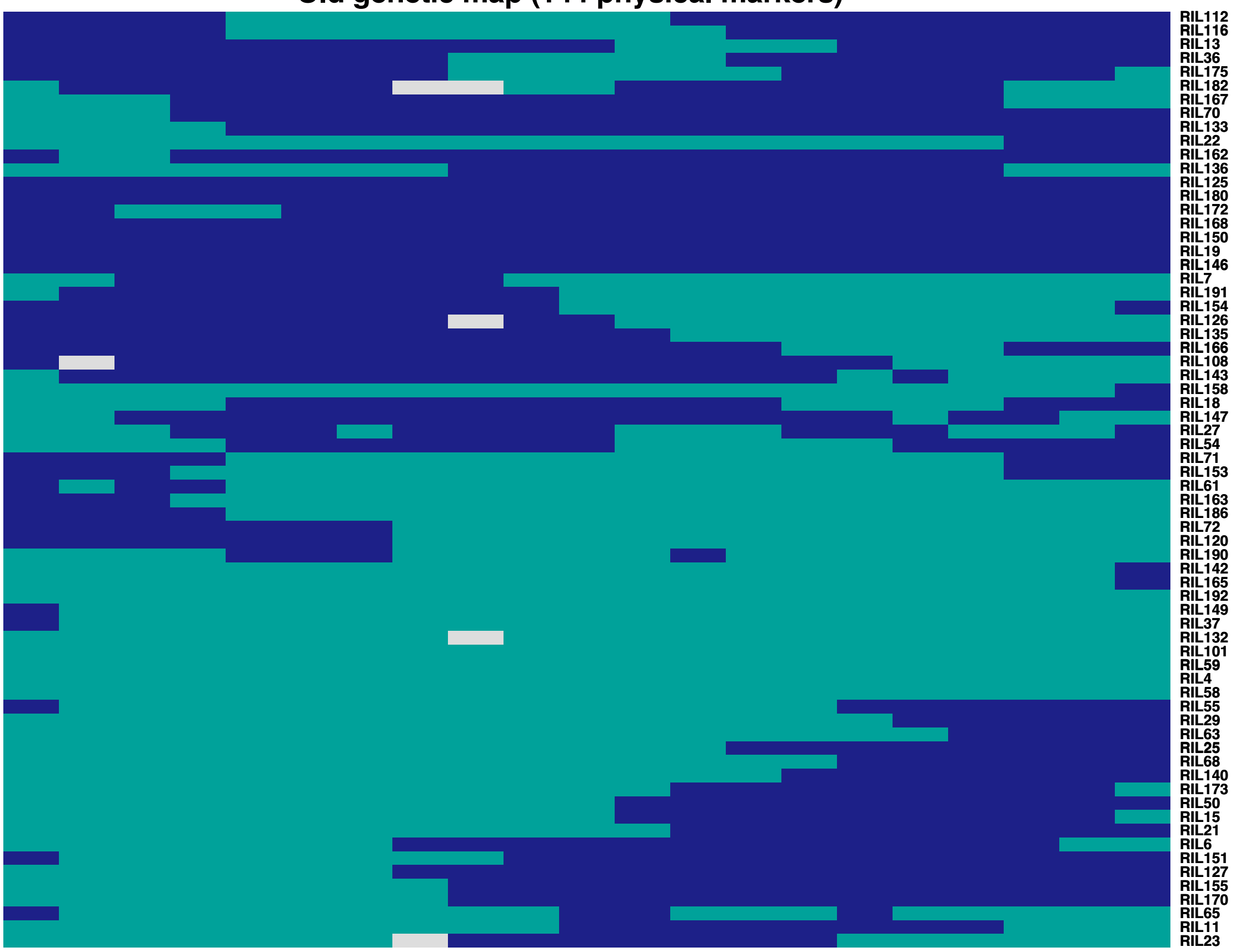

Supplementary Fig. 1

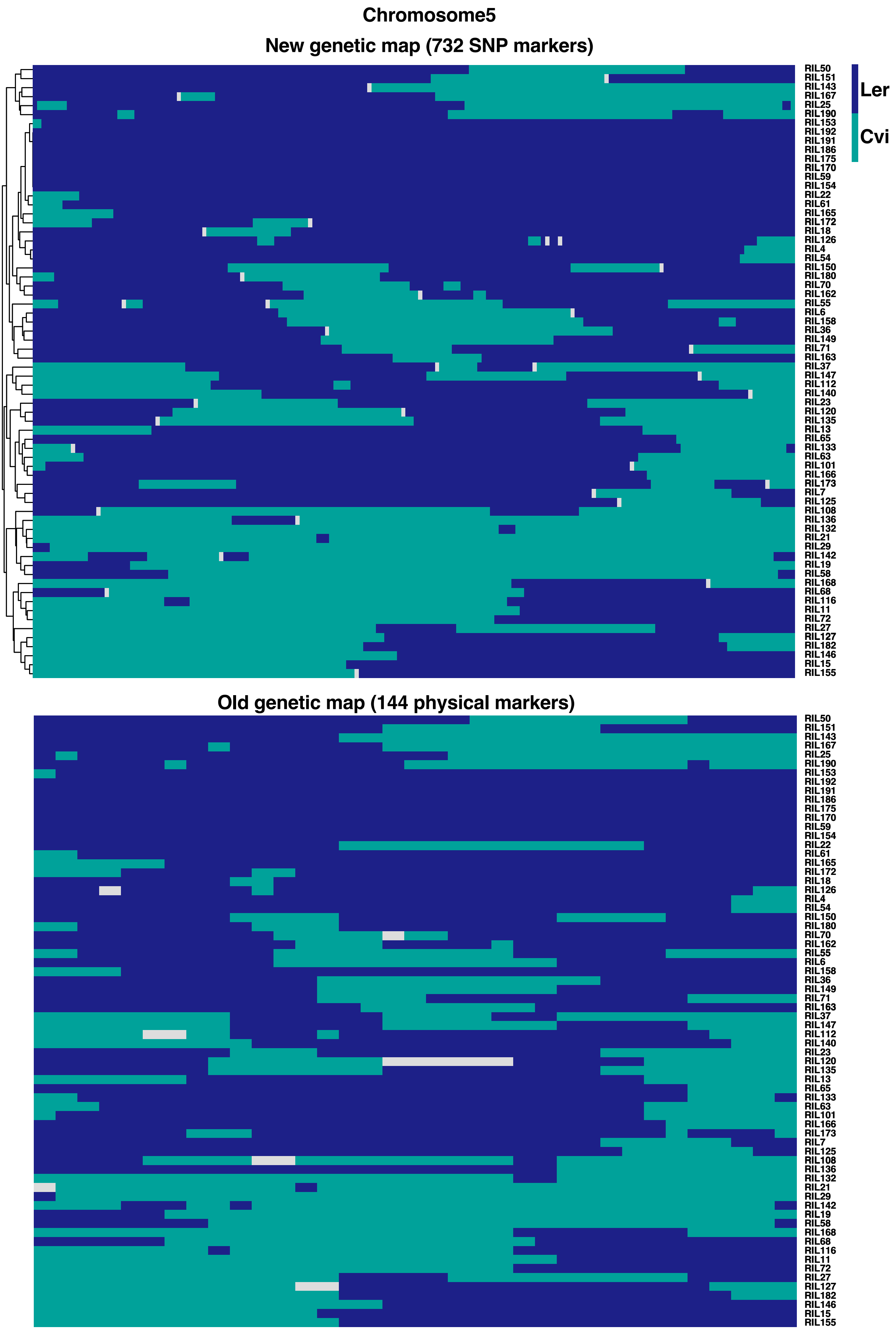

Supplementary Fig. 1

Supplementary Fig. 1 | Comparative visualization of genotype segments along chromosomes 1–5 using SNP-based and physical markers in the Ler × Cvi RILs population. Heatmaps depict the distribution of Ler, Cvi, and missing (white) genotype segments across recombinant inbred lines (RILs) along entire chromosomes. Each page corresponds to one chromosome (Chr1–5), comparing the high-resolution SNP-based genotype map (top) with the low-resolution physical marker map (bottom). Columns represent marker positions ordered by their genomic positions. Rows represent RILs and are consistently ordered across both maps for each chromosome to facilitate comparison, sorted by hierarchical clustering results based on SNP markers. In the top SNP-based panel, missing genotype segments at the edges of some SNP markers result from windows with an equal proportion of Ler and Cvi variants, which were classified as missing data during the sliding-window SNP assignment. The high-density SNP map recapitulates the overall genotype distribution patterns captured by the physical marker map, while substantially improving the accuracy and resolution of genotype inference by reducing missing data and revealing finer-scale recombination breakpoints. This refined genetic map enabled higher-precision QTL mapping and more accurate localization of candidate genomic regions.

VIM2/ORTH5 (AT1G66050) and *VIM4/ORTH4* (AT1G66040)

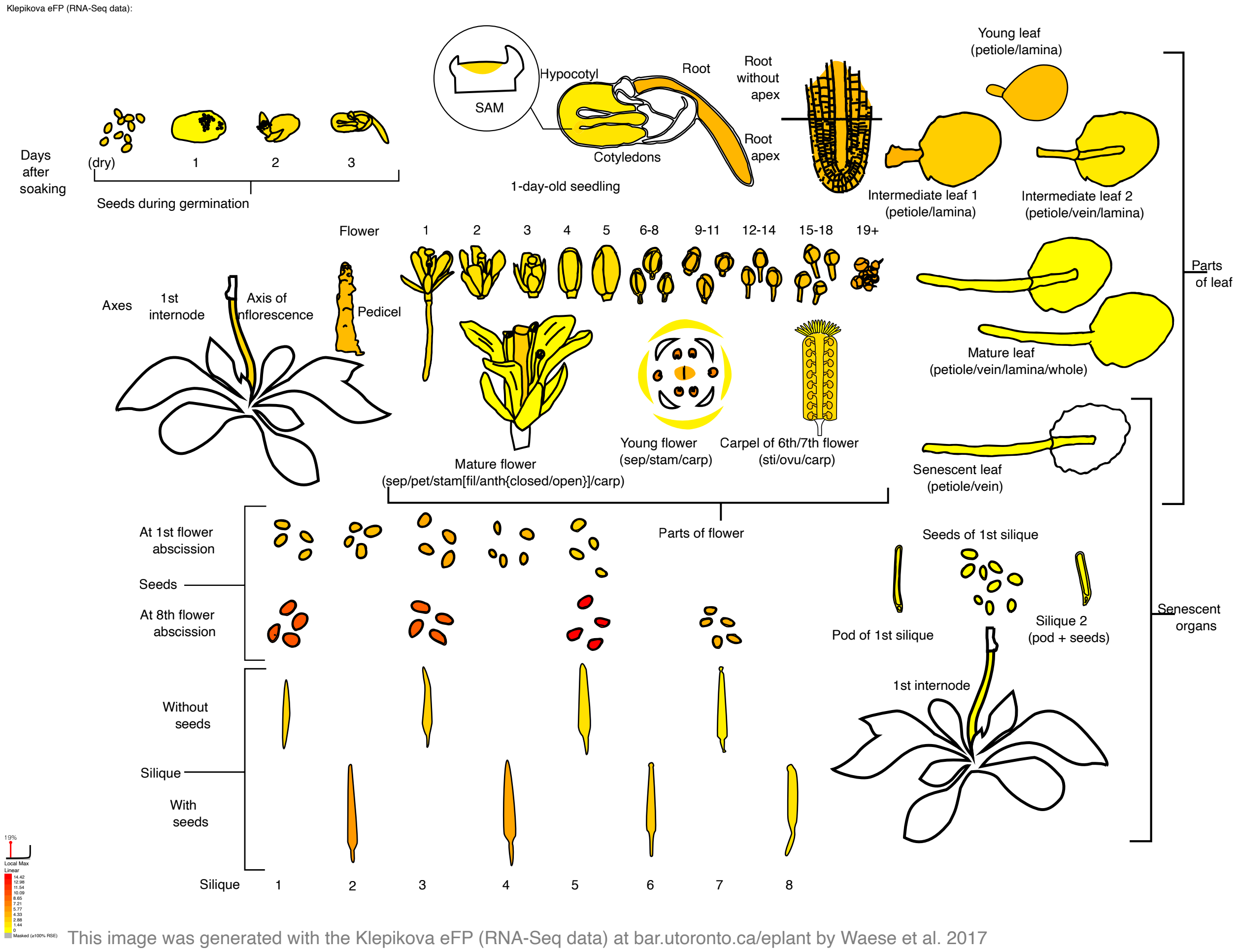

Supplementary Fig. 2. | Tissue- and developmental stage-specific expression of *VIM2/4* in *Arabidopsis thaliana*. Expression profiles for *VIM2* (AT1G66050) and *VIM4* (AT1G66040) across *Arabidopsis* tissues and developmental stages were retrieved from the *Arabidopsis* ePlant tool at bar.utoronto.ca/eplant (Waese et al., 2017 [46]) using the Klepikova RNA-seq dataset (Klepikova et al., 2016 [47]). Both queries returned identical expression profiles, consistent with the >99% coding sequence identity between *VIM2* and *VIM4*, which may prevent reliable distinction of the two paralogs using standard RNA-seq-based expression resources. The displayed expression values are therefore interpreted as the combined expression of *VIM2* and *VIM4* (i.e., *VIM2/4*). The profile indicates that *VIM2/4* expression is broadly detected across major tissues and developmental stages. Color intensity reflects the expression level, as indicated by the scale bar. The image was obtained directly from ePlant without reprocessing of the underlying RNA-seq data, and is used under a Creative Commons Attribution 4.0 International License (CC BY 4.0; https://creativecommons.org/licenses/by/4.0/). The image was cropped and resized, and its heading was modified to indicate the identical profiles; no other changes were made.

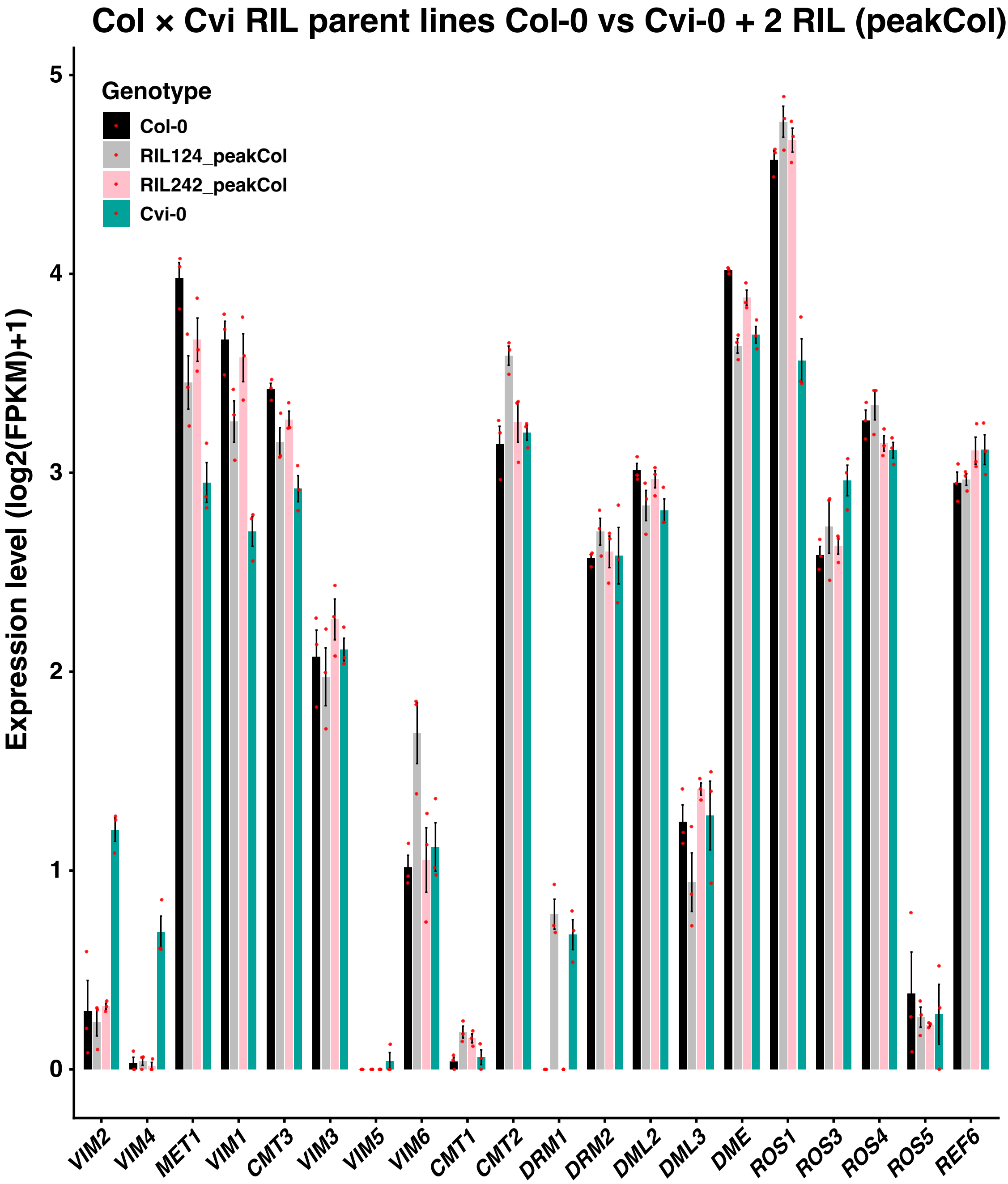

Supplementary Fig. 3 | Expression levels of DNA methylation pathway genes in an independent Col × Cvi RIL population and parental accessions. Bar plot showing expression levels of core DNA methylation maintenance and demethylation pathway genes in the Col-0 and Cvi-0 parental accessions, as well as in two Col × Cvi RILs (RIL124 and RIL242) carrying the Col genotype at the chromosome 1 QTL peak (peakCol). Expression values are shown as log2(fragments per kilobase of transcript per million mapped fragments (FPKM) + 1). These two RILs are the only lines with available transcriptome data in this dataset; transcriptome data for RILs carrying the Cvi genotype at the QTL peak are unavailable. Consistent with findings in the Ler × Cvi RIL population, elevated *VIM2* and *VIM4* expression in Cvi-0 is accompanied by downregulation of core methylation maintenance and demethylation genes, notably *MET1*, *VIM1*, *CMT3*, and *ROS1*, relative to Col-0. By contrast, the two peakCol RILs display expression patterns more similar to Col-0 at these loci. Transcriptome data were obtained from a published Col × Cvi RIL dataset (GEO accession GSE52814). Bars represent mean expression values; error bars indicate the standard error of the mean; and individual samples are shown as red dots.

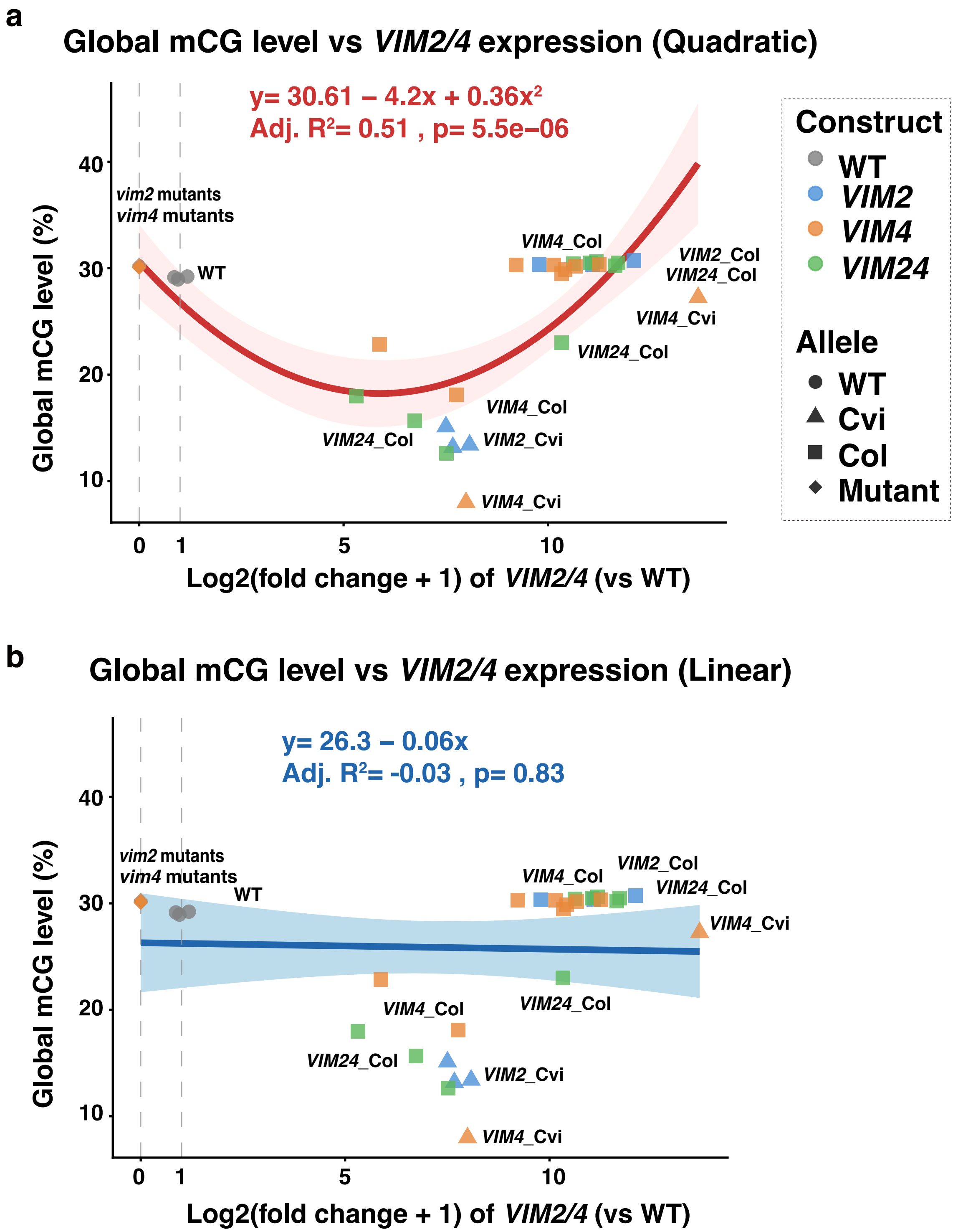

Supplementary Fig. 4 | Non-linear dosage relationship between *VIM2/4* expression and global CG methylation levels. Scatter plots showing the relationship between global mCG levels (%) and *VIM2/4* expression across Col-0 wild type, *VIM2*, *VIM4*, and *VIM24* overexpression lines, and *vim2* and *vim4* loss-of-function mutants. *VIM2/4* expression is shown as log2(fold change + 1) relative to wild type. Each point represents one independent transgenic line, mutant line or wild-type control (the unit of study; $n = 34$ in total), colored by construct type and shaped by allele or mutant background. Solid lines represent fitted regression curves, and shaded areas indicate 95% confidence intervals. Regression equations, adjusted R^2 values, and P values are shown in each panel. Grey vertical dashed lines indicate the mutant and wild-type reference values at $x = 0$ and $x = 1$, respectively. No adjustment for multiple comparisons was applied because only one overall regression test was performed. **a**, Quadratic regression of global mCG levels (%) against *VIM2/4* expression. The quadratic model captures a significant non-linear dosage relationship: moderate *VIM2/4* overexpression produces the strongest CG hypomethylation, whereas lines with extremely high expression (>1000-fold relative to wild type) retain near-wild-type mCG levels. The solid line shows the fitted quadratic regression, and the shaded band represents its 95% confidence interval. Statistical significance of the regression was assessed by a two-sided F-test of overall model significance ($F(2, 31) = 18.37$, $P = 5.47\text{e-}06$); the effect size is given by the adjusted $R^2 = 0.51$. **b**, Linear regression applied to the same data yields a poor fit (adjusted $R^2 = -0.03$, two-sided F-test of overall model significance, $P = 0.83$), confirming that the dosage effect of *VIM2/4* on CG methylation is non-monotonic and cannot be described by a simple linear model.

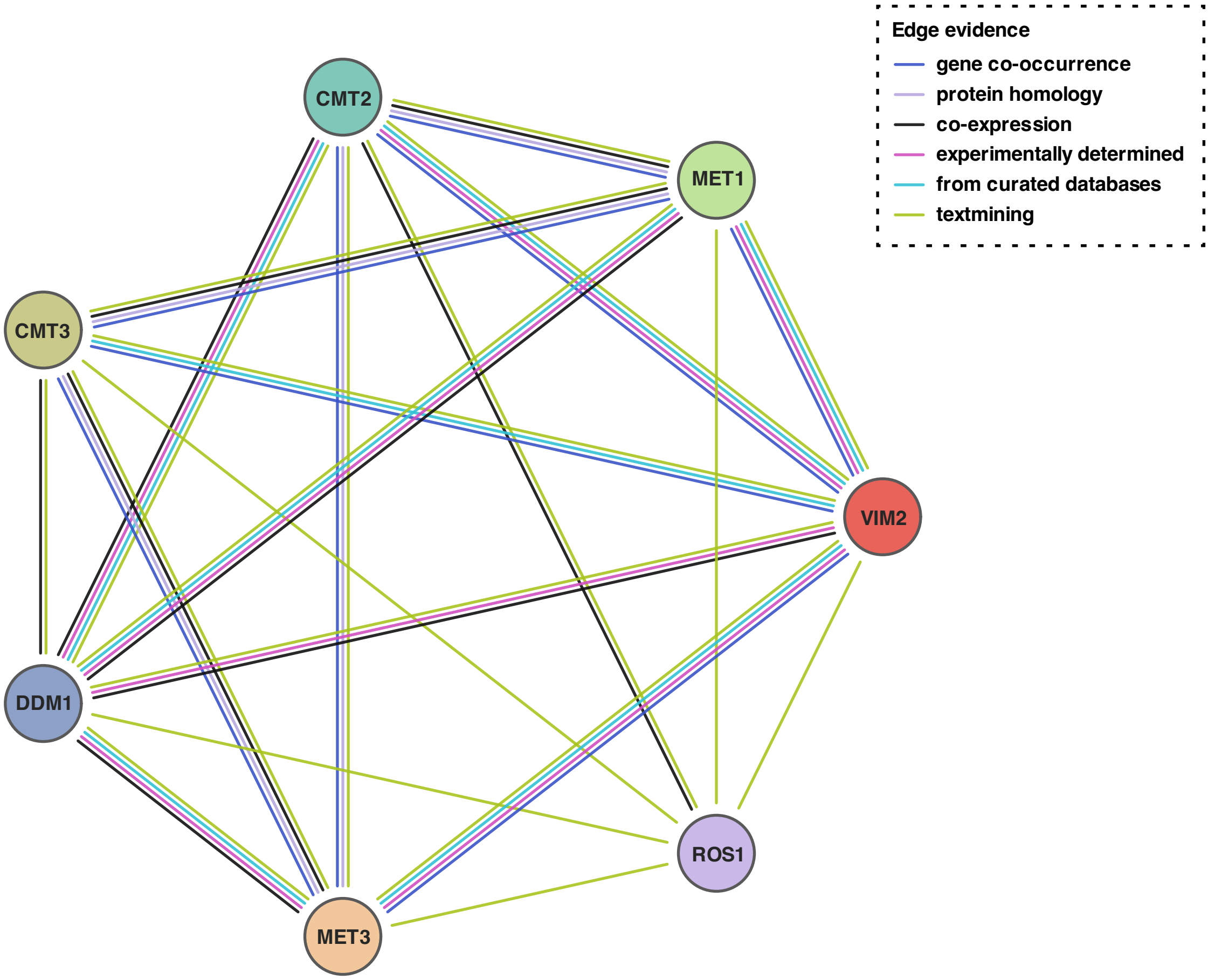

Supplementary Fig. 5 | Functional association network of VIM2 in *Arabidopsis thaliana*. The functional protein association network for VIM2/ORTH5 (AT1G66050) was independently visualized using interaction data retrieved with default settings from the STRING database (version 12.0; https://string-db.org; Szklarczyk et al., 2023 [60]). For clarity, the displayed network was redrawn with a restricted set of nodes, namely VIM2 and the key methylation pathway components MET1 (AT5G49160), MET3 (AT4G13610), CMT2 (AT4G19020), CMT3 (AT1G69770), DDM1 (AT5G66750), and ROS1 (AT2G36490). VIM2 shows strong functional associations with these components, consistent with its role in CG methylation maintenance. Nodes represent individual proteins, and edges indicate known and predicted protein-protein associations. Edge colors indicate the type and source of evidence supporting each association, as detailed in the inset legend. The underlying interaction data are available under a Creative Commons Attribution 4.0 International License (CC BY 4.0).

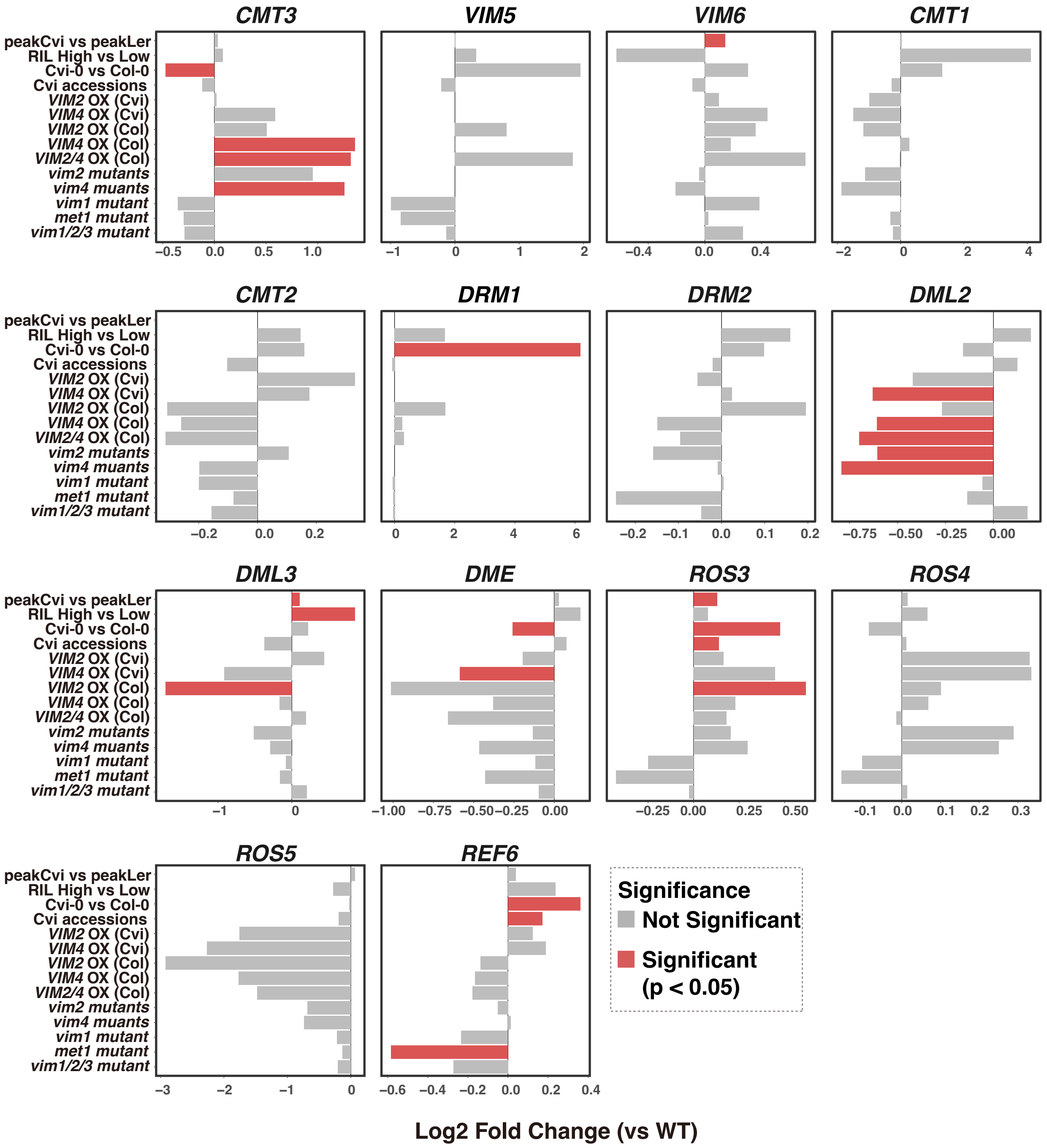

Supplementary Fig. 6 | Per-gene expression changes of additional DNA methylation regulators across multiple experimental contexts. Bar plots show per-gene expression changes of 14 additional DNA methylation maintenance and demethylation pathway genes across multiple experimental contexts, complementing the six key regulators shown in Fig. 5i-n. Genes shown include *CMT3* (AT1G69770), *VIM5* (AT1G57800), *VIM6* (AT4G08590), *CMT1* (AT1G80740), *CMT2* (AT4G19020), *DRM1* (AT5G15380), *DRM2* (AT5G14620), *DML2* (AT3G10010), *DML3* (AT4G34060), *DME* (AT5G04560), *ROS3* (AT5G58130), *ROS4* (AT3G14980), *ROS5* (AT1G54840), and *REF6* (AT3G48430). Bars show log2 fold changes relative to wild type (for overexpression and mutant lines) or between indicated contrasts (for RIL and accession comparisons). Datasets include Ler × Cvi RILs (PeakCvi versus PeakLer), RILs with high versus low epimutation rates, Cvi-0 versus Col-0, natural Cvi accessions stratified by presence versus absence of the Cvi-specific deletion, *VIM2/4* overexpression (OX) lines (Cvi and Col alleles), and *vim2*, *vim4*, *vim1*, *met1*, and *vim1/2/3* loss-of-function mutants. Significant differences ($P < 0.05$, two-sided Wald tests via DESeq2) are highlighted in red.

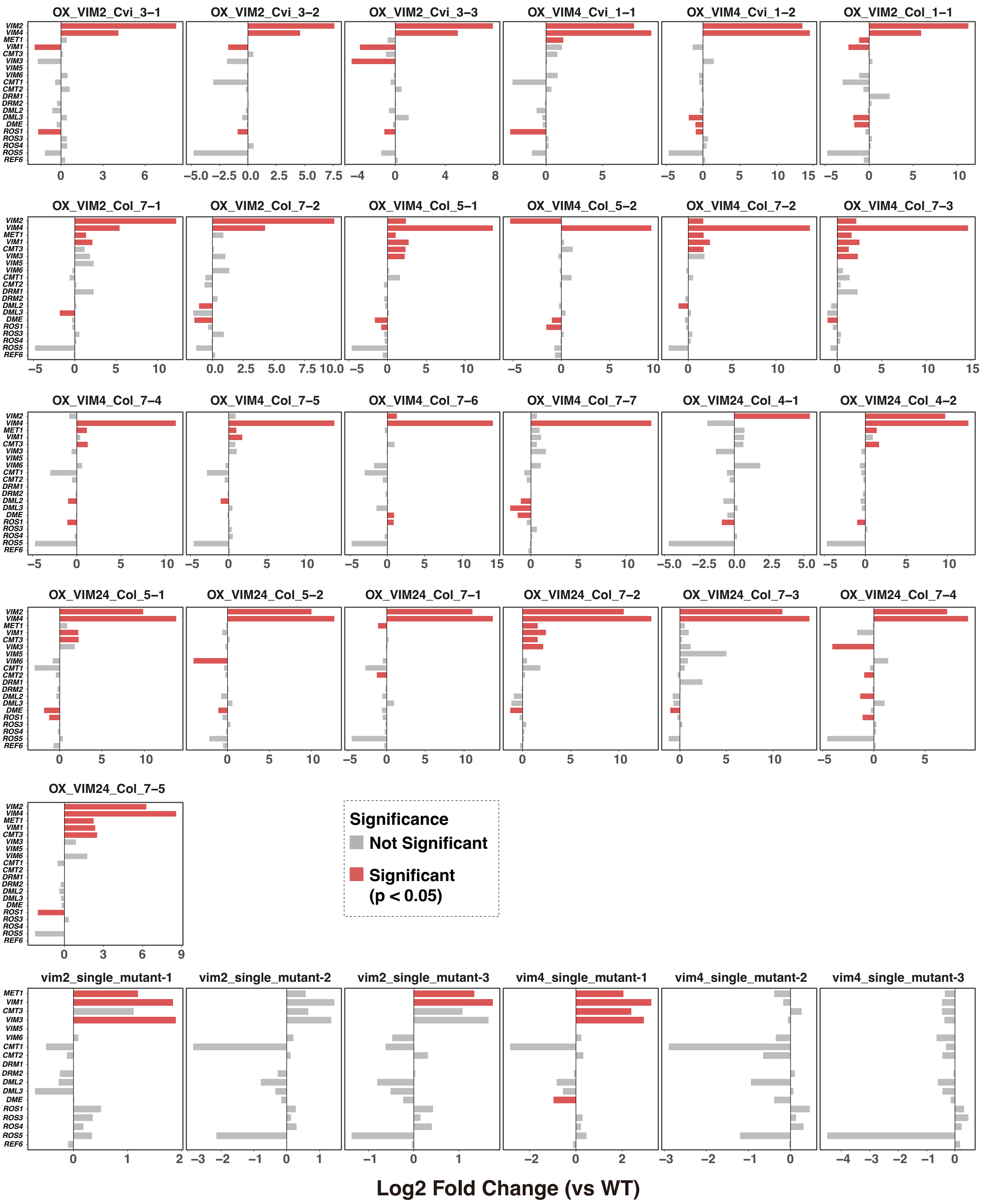

Supplementary Fig. 7 | Per-sample expression changes of DNA methylation regulators in individual *VIM2/4* overexpression lines and loss-of-function mutants. Per-sample expression changes of 20 DNA methylation and demethylation pathway genes in individual *VIM2*, *VIM4* and *VIM24* overexpression (OX) lines and individual biological replicates of *vim2* and *vim4* single mutants. Each panel represents one transgenic line or mutant replicate. Bars show log2 fold changes relative to the wild-type Col-0 controls. Positive values indicate higher expression relative to wild type. Samples include independent lines overexpressing *VIM2* with the Cvi allele (OX_VIM2_Cvi), *VIM4* with the Cvi allele (OX_VIM4_Cvi), *VIM2* with the Col allele (OX_VIM2_Col), *VIM4* with the Col allele (OX_VIM4_Col), and both *VIM2* and *VIM4* simultaneously carrying the Col allele (OX_VIM24_Col), as well as three biological replicates each for *vim2* and *vim4* single mutants. Significant differences ($P < 0.05$, two-sided Wald tests via DESeq2) are highlighted in red. This sample-level resolution complements the aggregated comparisons shown in Fig. 5i-n and Extended Data Fig. 8b-m, and demonstrates that individual overexpression lines vary in the magnitude and direction of secondary expression changes in genes such as *VIM1* and *VIM3*, consistent with the conclusion that CG hypomethylation in these lines cannot be explained by transgene-induced post-transcriptional gene silencing of *VIM1/3*.

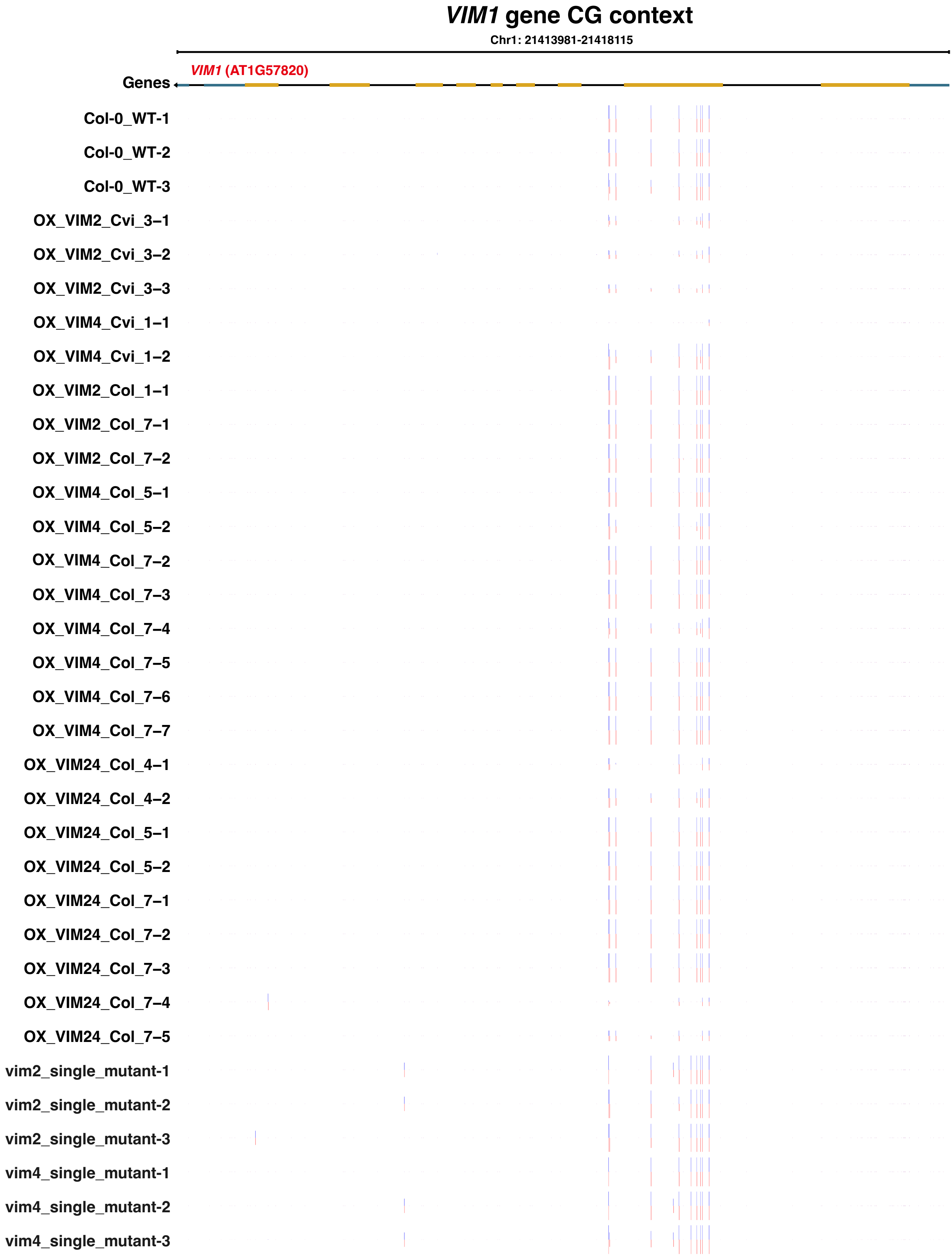

Supplementary Fig. 8

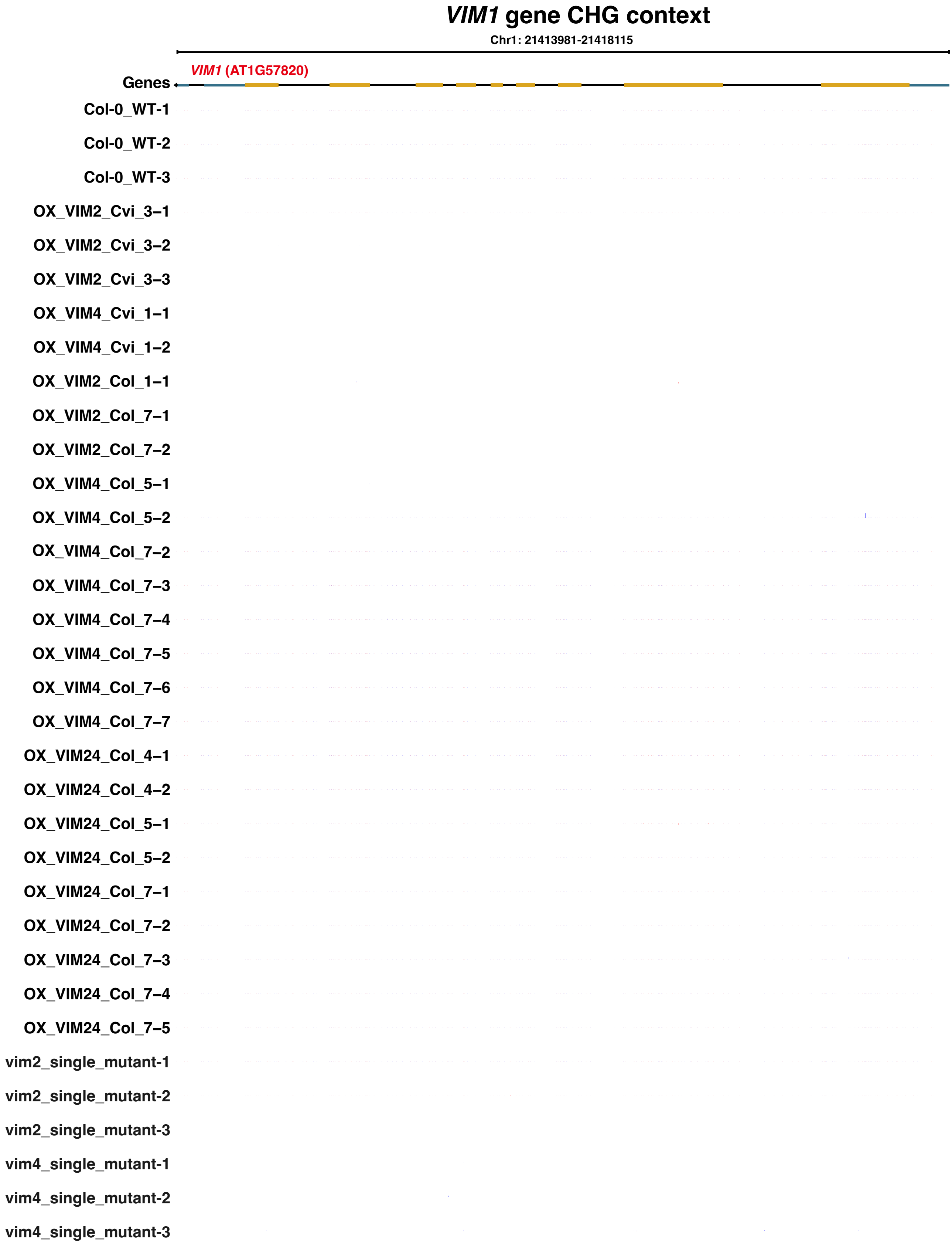

Supplementary Fig. 8

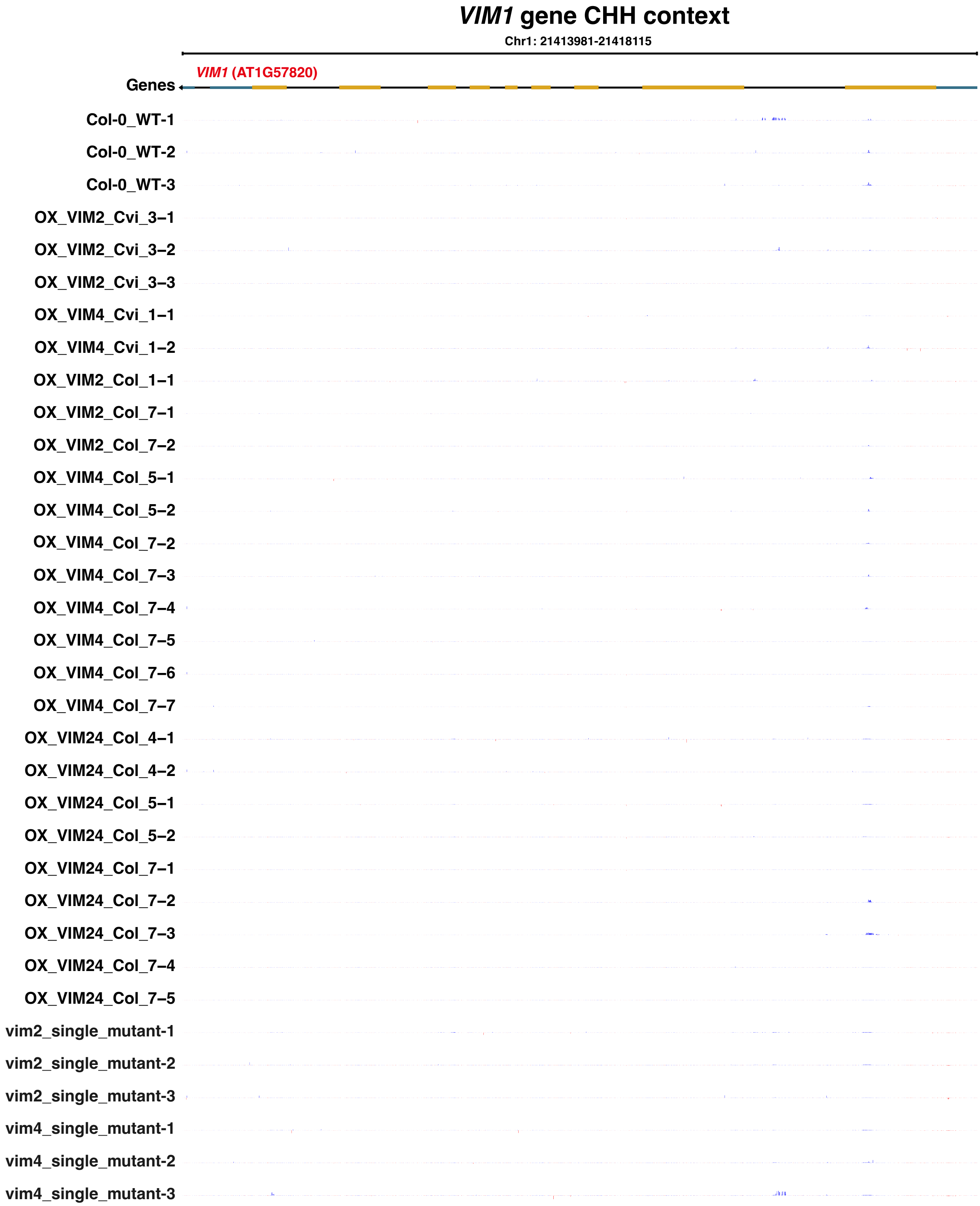

Supplementary Fig. 8

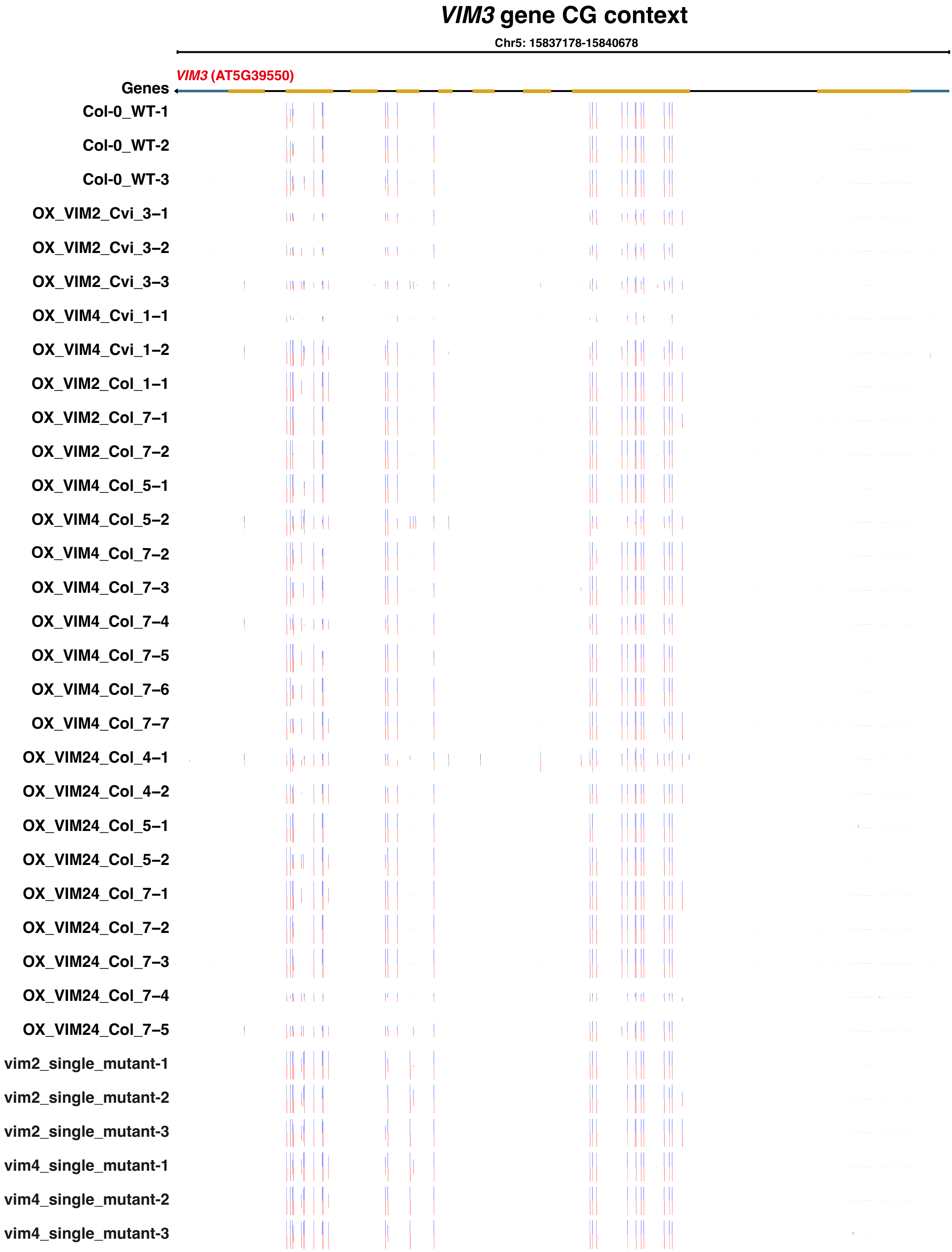

Supplementary Fig. 8

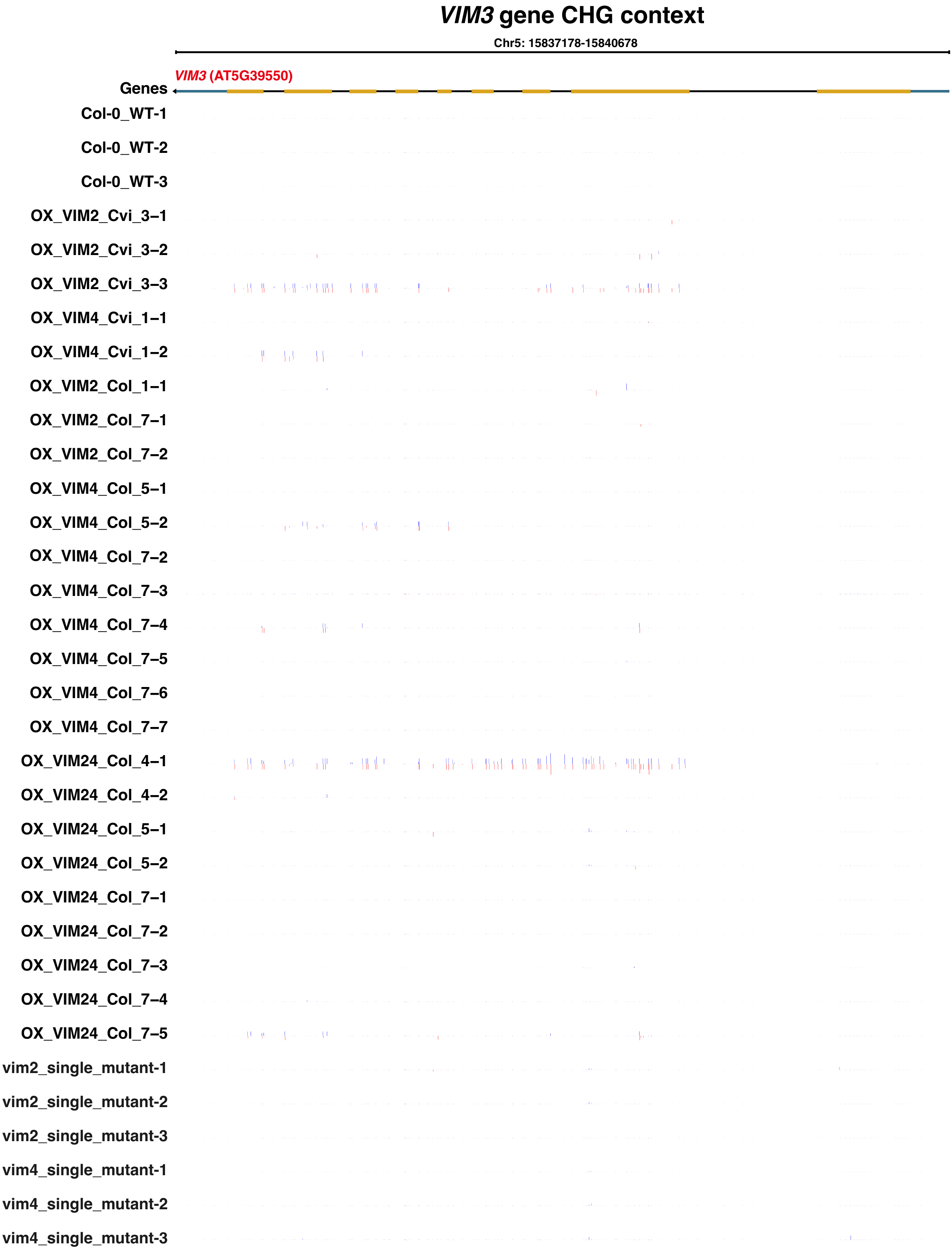

Supplementary Fig. 8

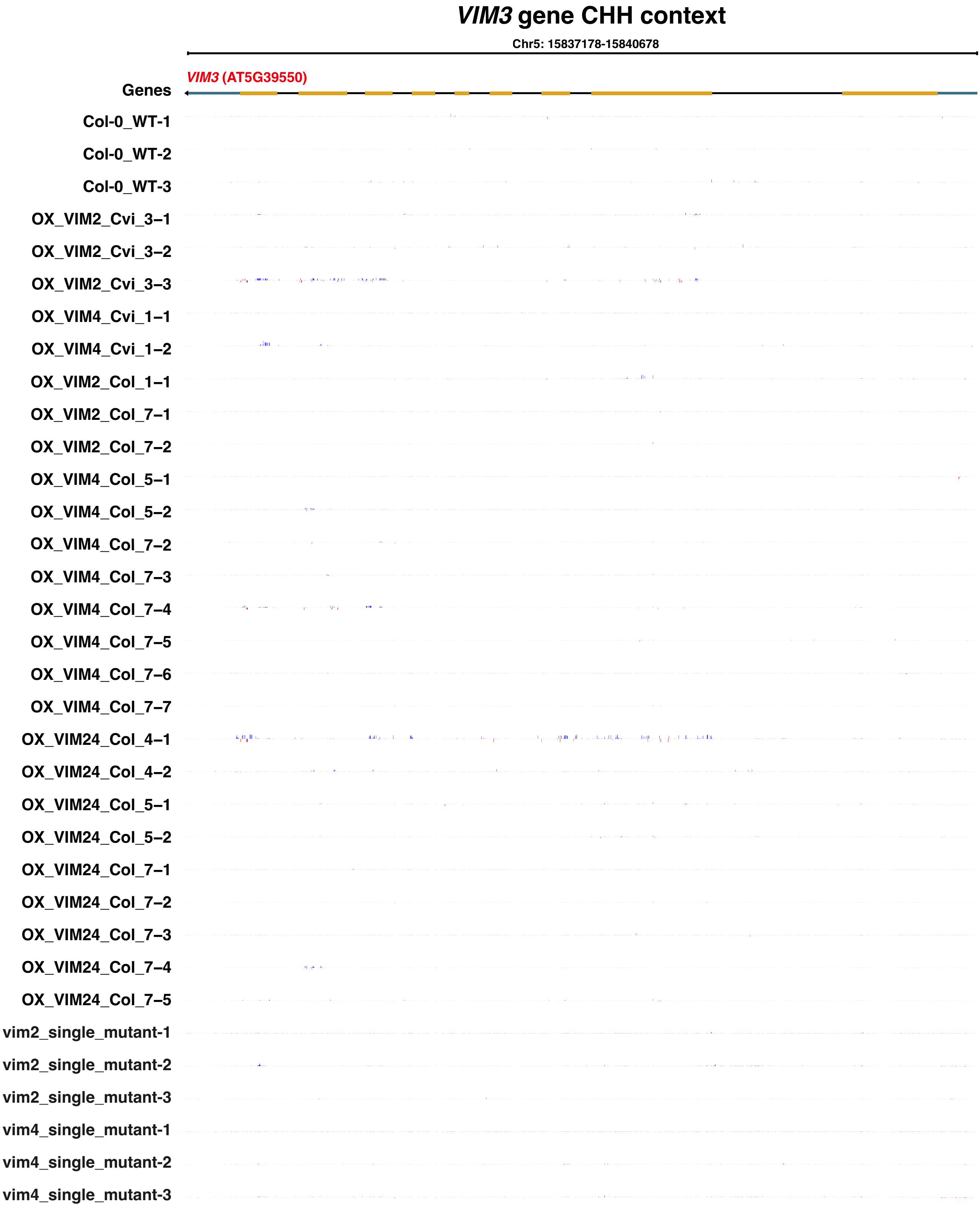

Supplementary Fig. 8 | DNA methylation profiles at the *VIM1* and *VIM3* genes across wild-type, *VIM2/4* overexpression lines, and loss-of-function mutants. Genome browser tracks display per-sample DNA methylation levels at the *VIM1* (AT1G57820) and *VIM3* (AT5G39550) loci in CG, CHG, and CHH sequence contexts. The figure consists of six pages: pages 1–3 show *VIM1* in the CG, CHG, and CHH contexts, respectively, and pages 4–6 show *VIM3* in the CG, CHG, and CHH contexts, respectively. Tracks are shown for Col-0 wild-type controls (three biological replicates), individual *VIM2*, *VIM4*, and *VIM24* overexpression (OX) lines carrying either the Cvi or Col allele, and three individual biological replicates each of *vim2* and *vim4* single mutants. Gene annotations are shown at the top of each page. The absence of consistent local increases in CG, CHG or CHH methylation across *VIM1* or *VIM3* in *VIM2/4* overexpression lines argues against transgene-induced post-transcriptional gene silencing (PTGS) of *VIM1/3* as the primary explanation for the CG hypomethylation observed in these lines.

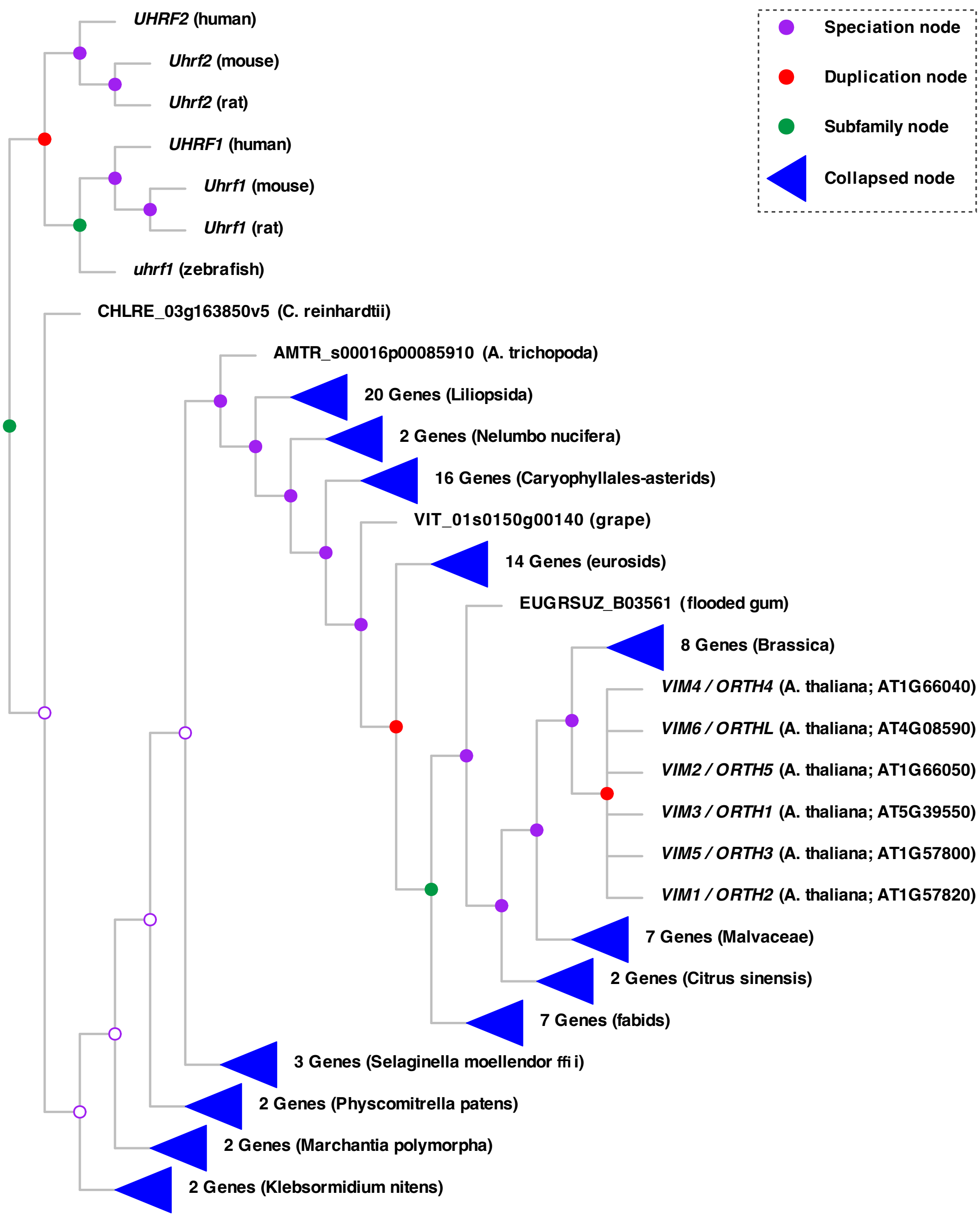

Supplementary Fig. 9 | Gene tree of the UHRF/VIM family. Gene-family tree of the UHRF/VIM family (E3 ubiquitin-protein ligase UHRF-related, PANTHER family PTHR14140) retrieved from the PhyloGenes database (release 5.0; https://phylogenes.arabidopsis.org; Zhang et al., 2020 [67]), which is built on the PANTHER 19.0 gene family release (Thomas et al., 2022 [68]). The family comprises 102 genes from 44 eukaryotic organisms. The tree shows that the six *Arabidopsis* VIM family members (VIM1 to VIM6) are homologs of the mammalian UHRF1 and UHRF2, supporting a shared evolutionary origin. An ancestral duplication event separating the UHRF1 and UHRF2 clades is evident in the sampled animal lineage, whereas additional lineage-specific duplications within the plant lineage, including the *Brassicaceae/Arabidopsis* clade, are consistent with the expansion of the VIM family in *Arabidopsis thaliana*. Purple circles denote speciation nodes, red circles denote duplication nodes, green circles denote subfamily nodes, and blue triangles represent collapsed clades. For clarity, the tree was redrawn from the source PhyloXML file for this study, with gene labels and the inset legend adjusted for readability. The clades outside the lineage of interest were collapsed, and *Arabidopsis* genes labeled by both their VIM and ORTH synonyms, together with their AGI locus identifiers. The underlying tree data are available under a Creative Commons Attribution 4.0 International License (CC BY 4.0).

a

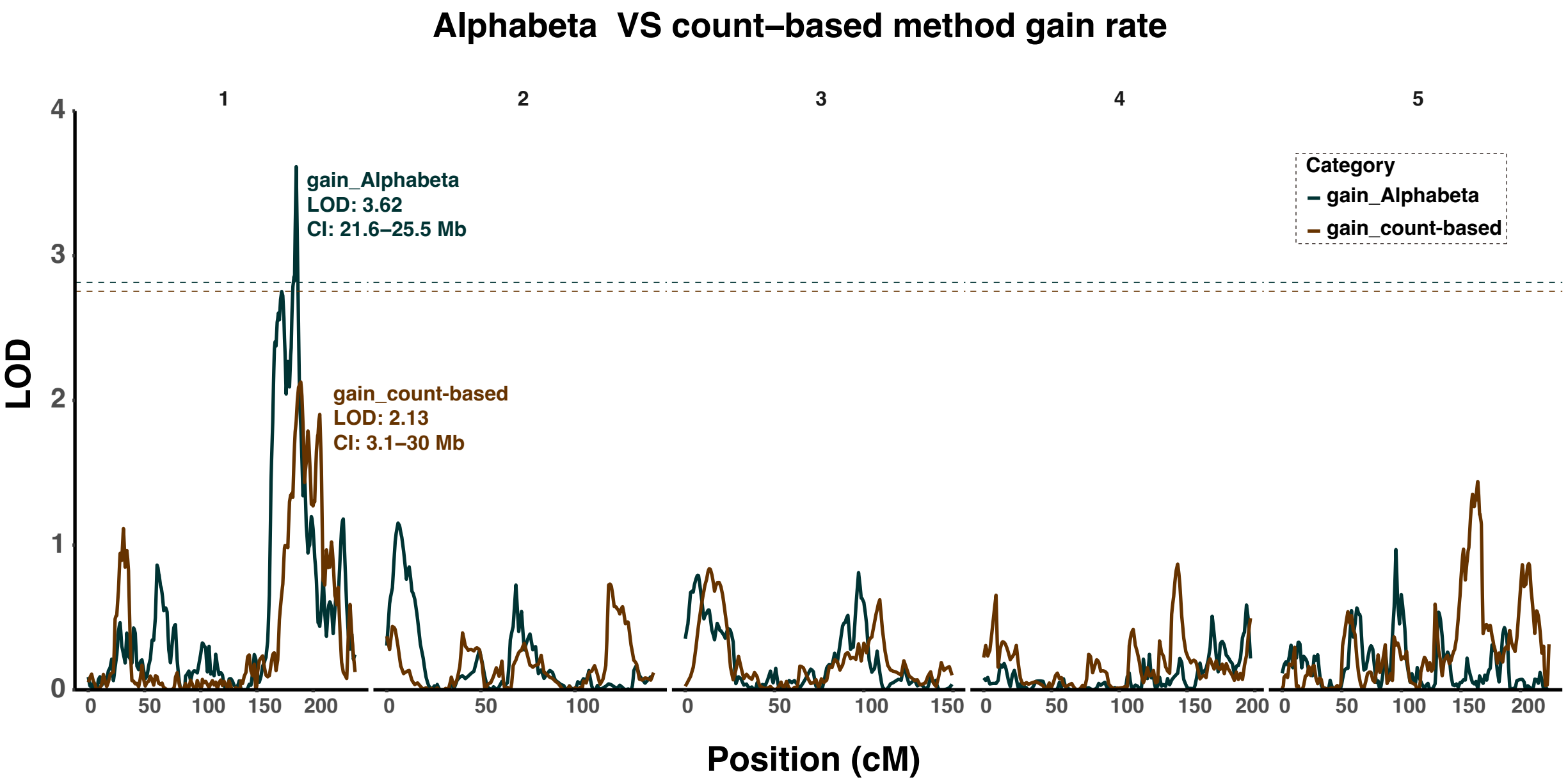

b

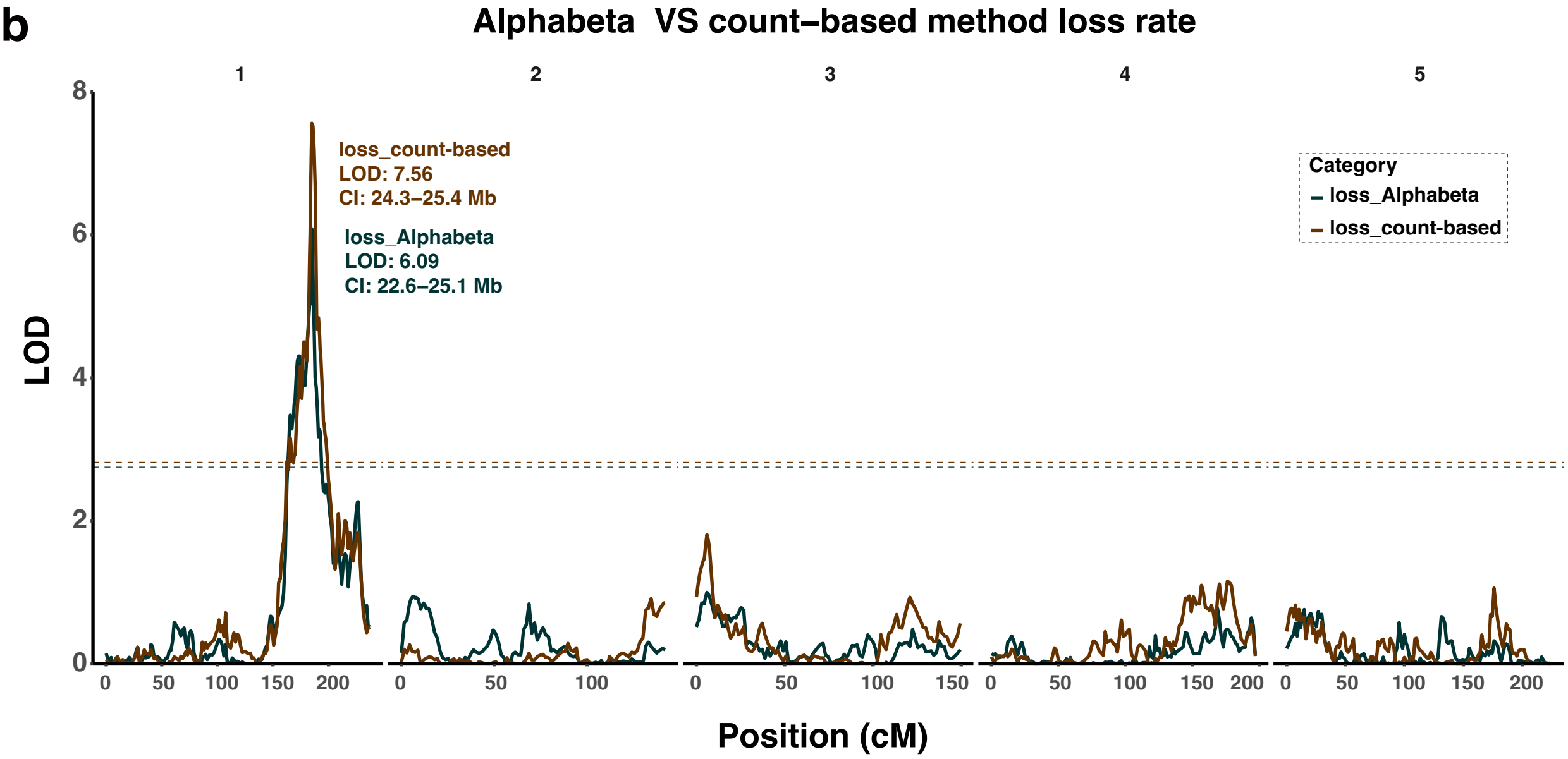

Supplementary Fig. 10 | Comparison of QTL mapping results using CG epimutation rates estimated by AlphaBeta and count-based methods. QTL scans for global CG methylation gain (a) and loss (b) rates compare rate estimates obtained using the AlphaBeta framework and the count-based method. **a,** For gain rates, the AlphaBeta framework identifies a significant QTL on chromosome 1 (LOD = 3.62; CI: 21.6–25.5 Mb), whereas the count-based method yields a sub-threshold peak at a similar chromosomal position (LOD = 2.13) with a broad interval spanning much of the chromosome (CI: 3.1–30.0 Mb), indicating poor mapping resolution. **b,** For loss rates, both methods detect a significant chromosome 1 QTL with overlapping intervals around the VIM2/4 locus (AlphaBeta: LOD = 6.09, CI: 22.6–25.1 Mb; count-based: LOD = 7.56, CI: 24.3–25.4 Mb). Peak logarithm of the odds (LOD) scores ("LOD") and Confidence Intervals ("CI") are indicated. Horizontal dashed lines represent significance thresholds determined by permutation testing ($\alpha = 0.05$, 1000 permutations). Position is given in centiMorgans (cM).

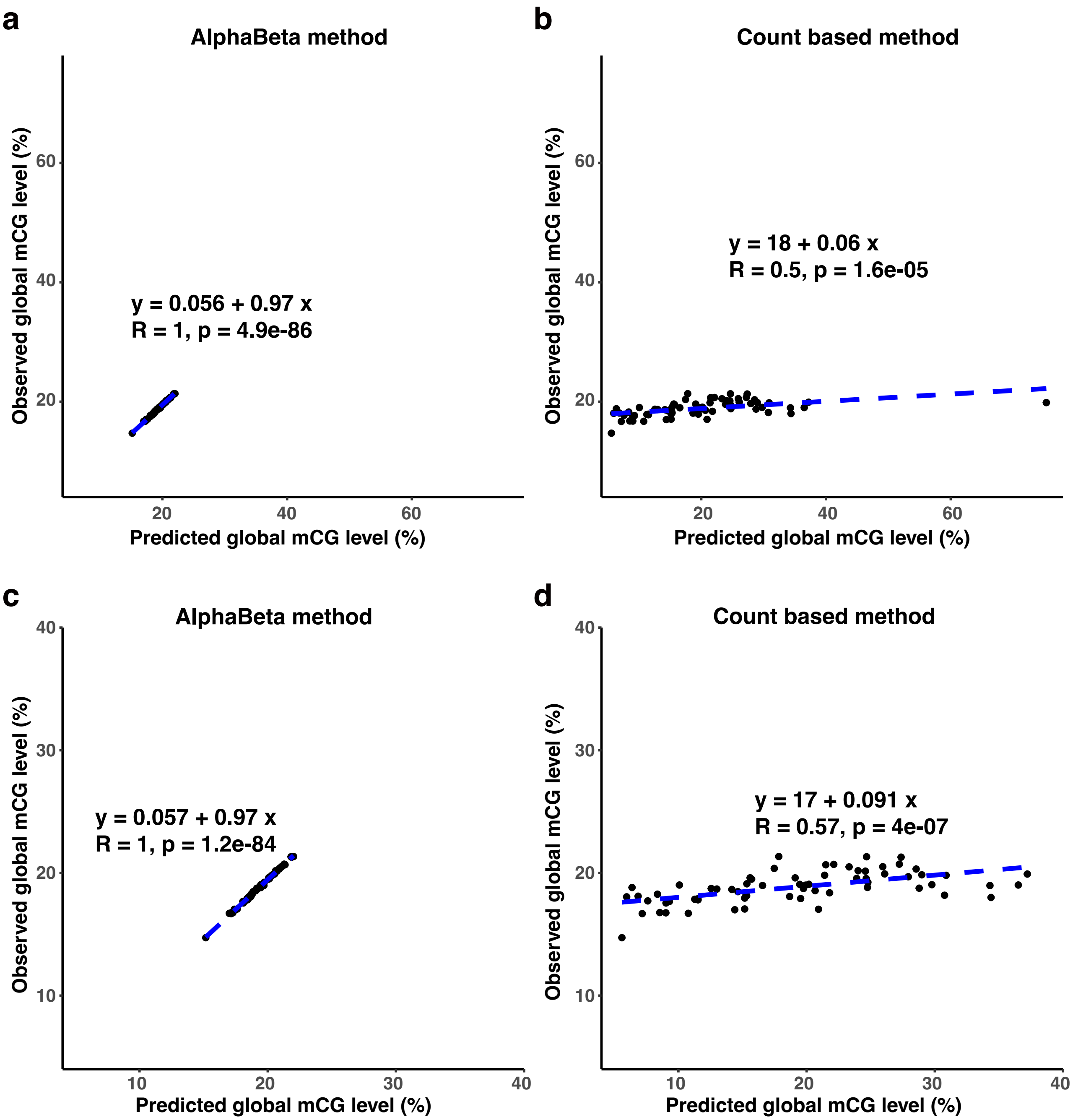

Supplementary Fig. 11 | Comparison of observed and predicted global mCG levels using epimutation rates from AlphaBeta and count-based models. Predicted global mCG levels were computed from epimutation rate estimates using the mathematical framework described by van der Graaf et al. (2015), which relates steady-state methylation to gain and loss rates. In all panels, each point represents one biologically independent RIL genotype and the dashed blue line indicates the linear regression fit. **a**, Correlation between observed global mCG levels and predicted levels calculated using epimutation rates derived from the AlphaBeta computational framework. The AlphaBeta model finds estimates of the gain and loss rates that are consistent with steady-state mCG methylation levels, which is reflected in the near-perfect correlation between predicted and observed mCG levels across RILs (n = 68 independent RILs; Pearson's $R = 1$, $p = 4.9\text{e-}86$). **b**, Correlation between observed global mCG levels and predicted levels using epimutation rates obtained from the count-based method. Predictions from the count-based method correlate more weakly with observed methylation (n = 68 independent RILs; Pearson's $R = 0.5$, $p = 1.6\text{e-}05$), highlighting that this naive estimator of gain and loss rates cannot ensure consistency with steady-state mCG levels. Notably, the count-based predictions span a substantially wider range than the observed values, with one RIL yielding a predicted mCG level of >70%, far exceeding the observed range. **c, d**, Same as in **a** and **b** after excluding this outlier RIL (n = 67 independent RILs). The AlphaBeta framework (**c**) retains near-perfect consistency (Pearson's $R = 1$, $p = 1.2\text{e-}84$) between predicted and observed global mCG levels, whereas the count-based method (**d**) remains more weakly correlated (Pearson's $R = 0.57$, $p = 4\text{e-}07$), with predicted values still spanning a broader range than the observed values. Statistical significance was assessed using two-sided Pearson's correlation tests; exact p-values are indicated for each panel. Together with Supplementary Fig. 10, these results demonstrate that the AlphaBeta framework provides more robust and reliable epimutation rate estimates for quantitative genetic analyses.
