# Supplementary material for: Mapping genetic modifiers of epimutation rates identifies VIM2/4 as dosage-sensitive negative regulators of CG methylation maintenance
Source: Nat Plants. 2026 Sep 3;12(10):1976–94. doi: 10.1038/s41477-026-02396-4 (PMC13680270; doi:10.1038/s41477-026-02396-4)
Supplement: Supplementary file 2 [file 41477_2026_2396_MOESM2_ESM.pdf]

nature portfolio

Corresponding author(s): Robert J. Schmitz, Frank Johannes

Last updated by author(s): Jul 21, 2026

Reporting Summary

Nature Portfolio wishes to improve the reproducibility of the work that we publish. This form provides structure for consistency and transparency in reporting. For further information on Nature Portfolio policies, see our Editorial Policies and the Editorial Policy Checklist.

Statistics

For all statistical analyses, confirm that the following items are present in the figure legend, table legend, main text, or Methods section.

n/a	Confirmed	
☐	☒	The exact sample size (*n*) for each experimental group/condition, given as a discrete number and unit of measurement
☐	☒	A statement on whether measurements were taken from distinct samples or whether the same sample was measured repeatedly
☐	☒	The statistical test(s) used AND whether they are one- or two-sided<br>*Only common tests should be described solely by name; describe more complex techniques in the Methods section.*
☒	☐	A description of all covariates tested
☐	☒	A description of any assumptions or corrections, such as tests of normality and adjustment for multiple comparisons
☐	☒	A full description of the statistical parameters including central tendency (e.g. means) or other basic estimates (e.g. regression coefficient) AND variation (e.g. standard deviation) or associated estimates of uncertainty (e.g. confidence intervals)
☐	☒	For null hypothesis testing, the test statistic (e.g. *F*, *t*, *r*) with confidence intervals, effect sizes, degrees of freedom and *P* value noted<br>*Give P values as exact values whenever suitable.*
☒	☐	For Bayesian analysis, information on the choice of priors and Markov chain Monte Carlo settings
☐	☒	For hierarchical and complex designs, identification of the appropriate level for tests and full reporting of outcomes
☐	☒	Estimates of effect sizes (e.g. Cohen's *d*, Pearson's *r*), indicating how they were calculated

Our web collection on statistics for biologists contains articles on many of the points above.

Software and code

Policy information about availability of computer code

Data collection	Publicly available FASTQ files analyzed in this study were downloaded using prefetch from SRA toolkit (version 2.9.2).
Data analysis	WGBS data, including our newly generated MA-ML, VIM2/4 Overexpression lines and mutants, as well as previously published datasets such as natural accessions and MA-accessions, were processed using the MethylStar pipeline (https://github.com/jlab-code/MethylStar). Cytosine-level methylation states were inferred using METHimpute. SNPs were identified from WGBS data using samtools (version 1.17) and bcftools (version 1.17), based on BAM files generated by MethylStar. Bedtools (version 2.30.0) was used for sub-setting the genome based on annotation files. Differentially methylated regions (DMRs) were identified using the jDMR pipeline (https://github.com/jlab-code/jDMRgrid). Epimutation rates were estimated using R package AlphaBeta (version 1.10.0; https://github.com/jlab-code/AlphaBeta). QTL mapping was performed using R package qtl (version 1.70).<br><br>For RNA-seq, raw reads were quality-checked with FastQC (version 0.11.9) and trimmed using Trimmomatic (version 0.38). Trimmed reads were aligned to the TAIR10 reference genome using STAR (version 2.7.6a), and alignments were sorted and indexed using SAMtools (version 1.17). Gene-level read counts were obtained using HTSeq-count (version 0.13.5) with the Araport11 annotation. Differential expression analysis was performed separately for each dataset using DESeq2 (version 1.30.1).<br><br>ATAC-seq reads were trimmed using Trimmomatic (version 0.39), aligned to the TAIR10 reference genome using BWA-MEM (version 0.7.17), deduplicated using Picard MarkDuplicates (version 2.23.8), and used for peak calling with MACS2 (version 2.2.9.1).<br><br>No ChIP-seq experiments were performed in this study. Previously published processed H3K27me3 ChIP-seq signal tracks for Col-0 were downloaded from the Plant Epigenome Browser (https://epigenome.genetics.uga.edu; Schmitz Lab, University of Georgia), based on data generated by Lu et al., and were used only to visualize the chromatin environment of the VIM2/4 intergenic region in Fig. 4a. No raw ChIP-seq

reads were reprocessed, and no new peak calling or antibody-based experiments were performed.

We now provide the R scripts (in scripts/), required input data (in data/), and output PDF figures (in figures/) for each applicable figure panel in the preprint. These files are included in both the public GitHub repository and the versioned Zenodo archive. A README file is included that maps each preprint figure panel to its corresponding script and input data files. We have also added workflow documentation in the workflow directory of our repository for WGBS, ATAC-seq, and RNA-seq. An R Markdown notebook documenting the entire analytical process from raw read processing to rate estimation and QTL scans is provided in workflow/WGBS/. See GitHub link (https://github.com/zhilinzhang340/Mapping-genetic-modifiers-of-epimutation-rates-codes-Zhilin_Zhang) and Zenodo link (https://doi.org/10.5281/zenodo.20387093)

For manuscripts utilizing custom algorithms or software that are central to the research but not yet described in published literature, software must be made available to editors and reviewers. We strongly encourage code deposition in a community repository (e.g. GitHub). See the Nature Portfolio guidelines for submitting code & software for further information.

Data

Policy information about availability of data

All manuscripts must include a data availability statement. This statement should provide the following information, where applicable:

- Accession codes, unique identifiers, or web links for publicly available datasets
- A description of any restrictions on data availability
- For clinical datasets or third party data, please ensure that the statement adheres to our policy

All sequencing data newly generated in this study have been deposited in the NCBI Gene Expression Omnibus (GEO). The WGBS data, including 371 MA-ML samples for 68 Ler × Cvi RILs and two parental MA-lines (Ler-0 and Cvi-0), have been deposited in the NCBI GEO under accession number GSE296957. WGBS data for Col-0 wild-type controls, vim2 and vim4 single mutants, and VIM2, VIM4, and VIM24 overexpression lines (Col-0 and Cvi-0 alleles) in the Col-0 background have been deposited under accession number GSE333205. RNA-seq data for Col-0 wild-type controls, vim2 and vim4 single mutants, VIM2, VIM4, and VIM24 overexpression lines, and six Ler × Cvi RILs selected for high or low epimutation rates have been deposited under accession number GSE333206. ATAC-seq data for two selected RILs have been deposited under accession number GSE297077.

Previously published datasets used in this study were obtained from the following sources. MA-accessions methylome data are available under GSE223810 17. Cvi accessions data were available in NCBI Sequence Read Archive(SRA) under BioProject numbers PRJNA612437 41. Previously published WGBS data for natural accessions were downloaded from GEO accession GSE43857 3, and corresponding transcriptome data were obtained from GSE80744 3. eQTL in the Ler × Cvi RILs population were obtained from previously published datasets 34,35. WGBS and transcriptome data for the Col × Cvi RILs population and Col-0 and Cvi-0 accessions were obtained from GEO accession GSE52814 56. RNA-seq data for H3K27me3-deficient mutants and corresponding wild type controls were obtained from GEO accession GSE81166 55. Long-read genome assemblies for Ler-0 and Cvi-0 were obtained from European Nucleotide Archive (ENA) projects PRJEB77668 48, and PRJEB55353 49, with an additional Cvi-0 assembly retrieved from NCBI BioProject PRJNA1033522 50. WGBS data for vim1, vim2, vim3, vim1/2/3, met1, and corresponding Col-0 wild type controls were obtained from GEO accession GSE39901 7. RNA-seq data for vim1, vim1/2/3, and met1 mutants and corresponding Col-0 wild type controls were obtained from NCBI BioProject PRJNA238327 57.

An overview of all datasets used in this study and their sources is provided in Supplementary Table 25.

Research involving human participants, their data, or biological material

Policy information about studies with human participants or human data. See also policy information about sex, gender (identity/presentation), and sexual orientation and race, ethnicity and racism.

Reporting on sex and gender	Not applicable. This study did not involve human participants.
Reporting on race, ethnicity, or other socially relevant groupings	Not applicable. This study did not involve human participants.
Population characteristics	Not applicable. This study did not involve human participants.
Recruitment	Not applicable. This study did not involve human participants.
Ethics oversight	Not applicable. This study did not involve human participants.

Note that full information on the approval of the study protocol must also be provided in the manuscript.

Field-specific reporting

Please select the one below that is the best fit for your research. If you are not sure, read the appropriate sections before making your selection.

☒ Life sciences ☐ Behavioural & social sciences ☐ Ecological, evolutionary & environmental sciences

For a reference copy of the document with all sections, see nature.com/documents/nr-reporting-summary-flat.pdf

Life sciences study design

All studies must disclose on these points even when the disclosure is negative.

Sample size

The MAML dataset consisted of 371 WGBS samples, including 68 RILs from a pre-established Ler × Cvi RILs and the parental accessions Ler-0 and Cvi-0. WGBS was performed on early- and late-generation samples as described in the Methods and Supplementary Table 2. Across MA-ML generations, RNA-seq was performed on 29 samples from six RILs with contrasting epimutation rates. ATAC-seq was performed on eight samples from two selected RILs, with four samples per RIL. Sample information is provided in Supplementary Tables 12 and 13.

For validation of VIM2/4 dosage effects, WGBS and RNA-seq were each performed on 34 samples, comprising 25 independent VIM2/4 overexpression lines, three Col-0 wild-type controls, and three biological replicates each for vim2 and vim4 mutants. Sample information is provided in Supplementary Tables 20 and 21.

For published datasets. For MA-accessions, 38 WGBS samples were generated from 4 natural ecotypes selected to represent the broad range of genome-wide CG methylation levels reported in A. thaliana.

For MAML data and MA-accessions, the sample sizes used in epimutation rate estimations are 1/2×N×(N−1), where N is the number of sequenced samples in each MA-pedigree. The chosen sample sizes are sufficient for epimutation rate estimations, as our in-house simulations showed that these sample sizes are sufficient to estimate the epimutation rates.

For natural accessions, raw WGBS data for 927 samples were downloaded from the 1001 Arabidopsis Methylomes project. After filtering for leaf tissue and removing redundant accessions (keeping the sample with the highest sequencing depth per ecotype), 850 unique accessions were retained. The corresponding transcriptome dataset comprised 728 samples from the same resource.

For natural Cvi accessions, 83 WGBS samples were used, comprising 40 accessions with the VIM2-VIM4 deletion and 43 accessions without the deletion (Supplementary Table 19).

For independent validation in the Col × Cvi RIL population, published data from Picard and Gehring (GEO accession GSE52814) were used: WGBS for the Col-0 and Cvi-0 parental accessions and 10 Col × Cvi RILs (6 carrying the Col and 4 carrying the Cvi haplotype at the VIM2/4 locus), together with transcriptome data for the two parental accessions and two of these RILs (RIL124 and RIL242, both carrying the Col haplotype), each profiled in 3 biological replicates.

Published mutant datasets included one WGBS methylome per genotype for vim1, vim2, vim3, vim1/2/3, met1 and the corresponding Col-0 wild-type controls, as well as RNA-seq data with three biological replicates per genotype for vim1, vim1/2/3, met1 and the corresponding Col-0 wild-type controls.

For the published H3K27me3-pathway mutant RNA-seq dataset, eight wild-type control samples and nine H3K27me3-pathway mutant samples were reanalyzed from the count stage using DESeq2.

An overview of all datasets used in this study and their sources is provided in Method and Supplementary Table 25.

Data exclusions

No data were excluded.

Replication

Findings were supported by multiple independent experimental and analytical layers. The chromosome 1 QTL was detected using 68 Ler × Cvi RIL-derived MA pedigrees and was validated across early and late generations. The Cvi-specific deletion was confirmed by PCR genotyping in selected RILs and shown to segregate with genotype. Its regulatory effect on VIM2/4 expression was independently validated by qPCR, showing consistent upregulation in deletion-containing lines. The association between the deletion and reduced CG methylation was further tested in natural Cvi accessions and in an independent Col × Cvi RIL population. The functional effect of VIM2/4 dosage was validated using independent VIM2/4 overexpression lines and reciprocal vim2 and vim4 loss-of-function mutants, with biological replicates for mutant analyses.

For the newly generated experiments, vim2 and vim4 mutants were each profiled in three biological replicates, the overexpression set comprised multiple independent transgenic lines per construct, and MA-ML sampled early and late generations across lineages. Key findings were reproduced across multiple independent systems, including Ler × Cvi RILs, an independent Col × Cvi RIL population, natural Cvi accessions, transgenic overexpression lines, and loss-of-function mutants.

Randomization

Samples were not allocated randomly into experimental groups, as the study design was based on existing natural and experimental populations. All plants were grown under the same environmental conditions and propagated for multiple generations. The recombinant inbred lines randomly segregate Ler and Cvi alleles, providing a natural randomization of genotype with respect to genetic background for the QTL analyses.

Blinding

Blinding is not relevant to our study, as there was no group allocation during data collection. Potential bias was minimized by using standardized sequencing workflows and automated computational pipelines for methylation, expression, chromatin-accessibility and QTL analyses.

Reporting for specific materials, systems and methods

We require information from authors about some types of materials, experimental systems and methods used in many studies. Here, indicate whether each material, system or method listed is relevant to your study. If you are not sure if a list item applies to your research, read the appropriate section before selecting a response.

Materials & experimental systems

n/a	Involved in the study
☒	☐ Antibodies
☒	☐ Eukaryotic cell lines
☒	☐ Palaeontology and archaeology
☒	☐ Animals and other organisms
☒	☐ Clinical data
☒	☐ Dual use research of concern
☐	☒ Plants

Methods

n/a	Involved in the study
☒	☐ ChIP-seq
☒	☐ Flow cytometry
☒	☐ MRI-based neuroimaging

Plants

Seed stocks

Seeds of the Ler × Cvi recombinant inbred line population were kindly provided by Joost J. B. Keurentjes. Homozygous T-DNA insertion mutant lines for vim2 (Stock Number: SALK_141723C; NASC ID: N664186) and vim4 (Stock Number: SALK_073322C; NASC ID: N675753) were obtained from the Nottingham Arabidopsis Stock Centre (NASC).

Novel plant genotypes

Novel transgenic Arabidopsis thaliana genotypes were generated for VIM2/4 overexpression analyses. Five VIM2/4 overexpression constructs were produced in the Col-0 background, carrying Col-0 or Cvi-0 alleles of VIM2 and/or VIM4. All constructs used the CaMV 35S promoter and NOS terminator and contained no protein tags. Plasmids were validated by Oxford Nanopore whole-plasmid sequencing, and T1 transgenic plants were confirmed by qPCR before WGBS and RNA-seq analyses. Details are provided in the Methods and Supplementary Table 23.

Authentication

The identity and genotype of plant materials were verified through independent approaches. WGBS-based SNP calls from all analyzed lines showed high concordance with the physical genetic map of the Ler × Cvi RIL population, confirming correct sample identity.

The naturally occurring Cvi-specific deletion was authenticated by PCR genotyping in selected Ler × Cvi RILs, confirming its genotype-specific segregation. Its regulatory impact on VIM2/4 expression was independently validated by qPCR, showing consistent upregulation in deletion-containing lines.

For the overexpression lines, constructs were verified by whole-plasmid sequencing, and overexpression was confirmed by qPCR. Single-nucleotide polymorphisms (SNPs) extracted from the WGBS data were utilized to verify the uniform Col-0 genetic background of all VIM2/4 overexpression lines, as well as the correct identity of the introduced Col or Cvi allele of the VIM2/4 transgene in each line. T-DNA insertion vim2 and vim4 single mutant lines are in the Col-0 background and were confirmed as homozygous by the SALK Institute prior to distribution. The T-DNA insertion lines were independently verified by PCR-based genotyping using OneTaq DNA Polymerase (#M0480, NEB) as previously described 76, and were confirmed by Sanger sequencing.
